# Genetic history of East-Central Europe in the first millennium CE

**DOI:** 10.1186/s13059-023-03013-9

**Published:** 2023-07-24

**Authors:** Ireneusz Stolarek, Michal Zenczak, Luiza Handschuh, Anna Juras, Malgorzata Marcinkowska-Swojak, Anna Spinek, Artur Dębski, Marzena Matla, Hanna Kóčka-Krenz, Janusz Piontek, Polish Archaeogenomics Consortium Team, Marek Figlerowicz

**Affiliations:** 1 Institute of Bioorganic Chemistry, Polish Academy of Sciences, Noskowskiego 12/14, 61-704 Poznan, Poland; 2 Institute of Human Biology & Evolution, Faculty of Biology, Adam Mickiewicz University, Poznan, Poland; 3 Institute of Immunology and Experimental Therapy, Polish Academy of Sciences, Wroclaw, Poland; 4 Department of Archaeology, Collegium Historicum, Adam Mickiewicz University, Poznan, Poland; 5 Department of History, Collegium Historicum, Adam Mickiewicz University, Poznan, Poland

## Abstract

**Background:**

The appearance of Slavs in East-Central Europe has been the subject of an over 200-year debate driven by two conflicting hypotheses. The first assumes that Slavs came to the territory of contemporary Poland no earlier than the sixth century CE; the second postulates that they already inhabited this region in the Iron Age (IA). Testing either hypothesis is not trivial given that cremation of the dead was the prevailing custom in Central Europe from the late Bronze Age until the Middle Ages (MA).

**Results:**

To address this problem, we determined the genetic makeup of representatives of the IA Wielbark- and MA Slav-associated cultures from the territory of present-day Poland. The study involved 474 individuals buried in 27 cemeteries. For 197 of them, genome-wide data were obtained. We found close genetic affinities between the IA Wielbark culture-associated individuals and contemporary to them and older northern European populations. Further, we observed that the IA individuals had genetic components which were indispensable to model the MA population.

**Conclusions:**

The collected data suggest that the Wielbark culture-associated IA population was formed by immigrants from the north who entered the region of contemporary Poland most likely at the beginning of the first millennium CE and mixed with autochthons. The presented results are in line with the hypothesis that assumes the genetic continuation between IA and MA periods in East-Central Europe.

**Supplementary Information:**

The online version contains supplementary material available at 10.1186/s13059-023-03013-9.

## Introduction

The decline of the Western Roman Empire under the pressure of barbarian tribes led to the emergence of new political and ethnic structures [1–3]. While the historical events and processes underlying the transformation from Antiquity to Christianity in the territories of the Roman Empire are relatively well recognized, the processes that occurred in parallel outside the Empire, especially in the regions that did not belong to the newly formed Christian community [4, 5], are poorly understood, including the appearance of Slavs in Central Europe [6–17]. Two conflicting hypotheses have been formulated [18, 19] to explain the appearance of Slavs. The allochthonous hypothesis states that Slavs migrated to this region of Europe no earlier than the sixth century CE [20, 21], whereas the autochthonous hypothesis posits that Slavs inhabited the region between the Oder and Vistula Rivers long before the Migration Period, traditionally dated to between 375 CE (invasion of Europe by the Huns) and 568 (conquest of Italy by the Longobards) [22–24].

Data collected thus far indicate that at the end of the Late Neolithic period, 3700–1800 BCE, the genetic structures of the populations occupying Central Europe were stabilized and remained to a large extent unchanged until the end of the Bronze Age, 1800–700 BCE [25, 26]. Three main genetic components constituted the genomes of people living in this region at that time. The first component was linked to the Mesolithic western hunter-gatherers (WHGs), who came to Europe approximately 14 thousand years ago [27]. The second was associated with the Neolithic Anatolian farmers (NAFs), who migrated to Europe 7–8 thousand years ago [28]. The third was related to the Yamnaya steppe herders (YAMs) [26], who spread in East-Central Europe 4–5 thousand years ago. How the genetic makeup of Central Europe was shaped during the Iron Age (IA), 700 BCE–650 CE, remains an open question, given the lack of representative biological material for archaeogenomic studies, due to the prevalence of cremation among populations living in this region from the Bronze Age until the Middle Ages (MA) [29, 30].

In the studies presented here, we took advantage of the fact that for a limited period of time, inhumation became the dominant funeral practice in the region of contemporary Poland within the population associated with the Wielbark culture [31–33]. This population existed in the basin of the Vistula River between the first and fifth centuries and then disappeared. Some theories link the emergence of the Wielbark culture with the migration of people commonly referred to as Goths [32]. Archaeological findings indicate that until the fifth century CE, they lived alongside people associated with the preceding Przeworsk culture, who still practised cremation [34, 35]. The final stage of coexistence of the Wielbark and Przeworsk cultures in the present territory of Poland overlapped with the Migration Period [4]. After its end, material cultures in this region became more homogenous, and archaeologists commonly link them with the Slavs [8, 36] who practised cremation of the dead [29] until the first Polish ruling dynasty was baptized (in 966 CE).

Our recent analyses of mitochondrial genomes of IA groups associated with the Wielbark culture [37, 38] showed that from the matrilineal perspective, male individuals were genetically most similar to the IA southern Scandinavians, and their genetic history was different from that observed for females, who were associated most closely with the Middle Neolithic groups from Central Europe. This strongly suggests that peoples associated with the Wielbark culture were newcomers who mixed with the local population living in the territory of present-day Poland at the beginning of the CE. Thus, by identifying the genetic admixture present in the genomes of individuals from the Wielbark culture and comparing this admixture with the genomes of people living in the same region in MA, one can verify both the allochthonous and autochthonous hypotheses. Considering this information, we performed a whole-genome analysis of representatives of the IA Wielbark- and MA Slav-associated cultures.

## Results

### Studied group description

**Fig. 1:**
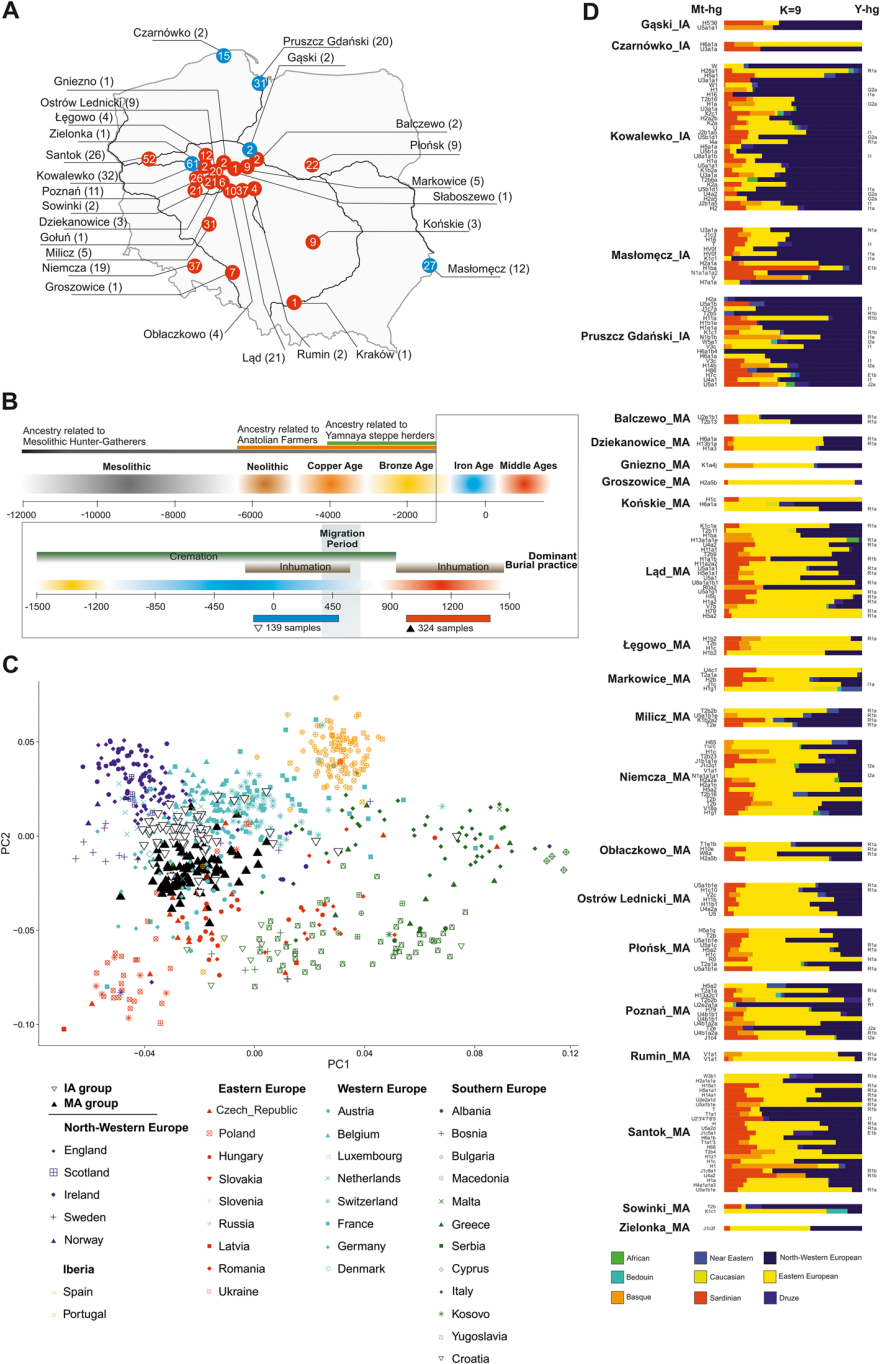
Spatiotemporal distribution of samples and their genetic affinities to present-day populations. **a** A map of present-day Poland showing the locations of skeletal remains sampled for this study. Colours denote the time periods of ancient individuals: red—Middle Ages (MA), blue—Iron Age (IA). Numbers inside coloured circles denote number of samples recovered from given site. Numbers in parentheses denote samples included in the analyses. **b** Representation of the context of the studied ancient individuals. **c** Ancient individuals projected onto the first two eigenvectors of a PCA based on contemporary European genetic diversity. **d** Ancestry proportions for ancient IA and MA individuals estimated using ADMIXTURE in unsupervised mode (*K* = 9)

To reconstruct the biological history of the population living in East-Central Europe before and after the Migration Period, we analysed DNA samples taken from 474 individuals buried in 27 cemeteries in contemporary Poland [34, 39–62] and radiocarbon dated [63, 64] to the IA (second century BCE to fourth century CE, 5 Wielbark culture cemeteries) and the MA (tenth to thirteenth century CE, 22 Slav-associated cemeteries) (Fig. 1a, b and Additional file 1: Figs. S1-S13). DNA was isolated and whole-genome sequencing was performed as previously described [28, 38]. For all samples, DNA showed patterns of post-mortem damage (PMD) characteristic of ancient DNA (Additional file 2: Table S1). In most samples, we found low levels of both X-chromosome- and mtDNA-based contamination with other human DNA (avg. of 1.3% and 2.2%, respectively). High X-chromosome-based and mtDNA-based contamination was detected in 2 and 15 samples, respectively (Additional file 2: Table S1), which were excluded from further analysis. The study set thus included 457 samples divided into two groups, the IA group (134 samples) and the MA group (323 samples). Genome-wide data suitable for further analyses (at least 10.000 SNPs for an individual for the PCA, ADMIXTURE and qpADM analyses) were obtained for 197 individuals, 68 and 129 from the IA and MA groups, respectively. The sex of 334 individuals was determined [65], for 97 from the IA (45% male) and for 237 from the MA (58% male) groups. Mitochondrial DNA haplogroups (mt-hgs) were assigned for 343 individuals (105 and 238 for the IA and MA groups, respectively), and Y-chromosome haplogroups (Y-hgs) were determined for 155 individuals (45 and 110 for the IA and MA groups, respectively) (Additional file 2: Table S3-4).

### IA and MA group affinities

To compare the genetic structures of the studied ancient populations and contemporary populations living in Europe, genome-wide data obtained for the individuals from the IA and MA groups were compared with the corresponding datasets describing the genetic variation of present-day West Eurasians (HO dataset) (Additional file 3: Fig. S14) and Europeans (POPRES dataset) (Fig. 1c). Principal component analysis (PCA, Fig. 1c) showed that both the IA and MA individuals overlapped with the present-day European populations. IA individuals were shifted towards peoples inhabiting Northwestern Europe, whereas MA individuals overlapped more with those living in Central and Eastern Europe.

To learn more about the genetic structure of the IA and MA populations, we performed unsupervised ADMIXTURE analyses separately for each studied individual, using present-day west Eurasians and Africans as the reference dataset (HO dataset) (Fig. 1d). For every studied person, we assessed his or her relation to 9 putative ancestral populations (*K* = 9). We found that the major genetic component present in the DNA of IA individuals was the one maximized in the present-day Northwestern European genomes, whereas the DNA of MA individuals had, on average, the highest contribution of the genetic component maximized in the genomes of contemporary East-Central Europeans (Additional file 7: Fig. S18, Additional file 8: Fig. S19). The PCA and ADMIXTURE results were further supported by the Fst coefficients, which showed smaller genetic distances between present-day Northwestern Europeans and IA individuals than between the former and MA individuals (Additional file 4: Fig. S15). Our analysis also revealed that numerous individuals from the IA group had genetic structure similar to individuals from the MA group, characterized by a high proportion (> 20%) of the genetic component observed in the highest frequencies in present-day East-Central European populations. In six cases, this fraction was substantial (> 40%) (Fig. 1d). We did not observe a similar Eastern European genetic component in ancient Northwestern populations (from the IA or MA periods) and detected this component at very low levels in present-day Northern European populations (Additional files 5–8: Figs. S16-S19). A genetic make-up similar to that of the IA group members was found in a few samples from a sixth century Longobard migration era-associated population living in Szolad, Hungary (Hungary_Szolad_Migration_Period) [1] and in an individual associated with the fourth century CE Chernyakhov culture from Ukraine [66] (Additional file 5: Fig. S16, Additional file 9: Fig. S20A).

**Fig. 2:**
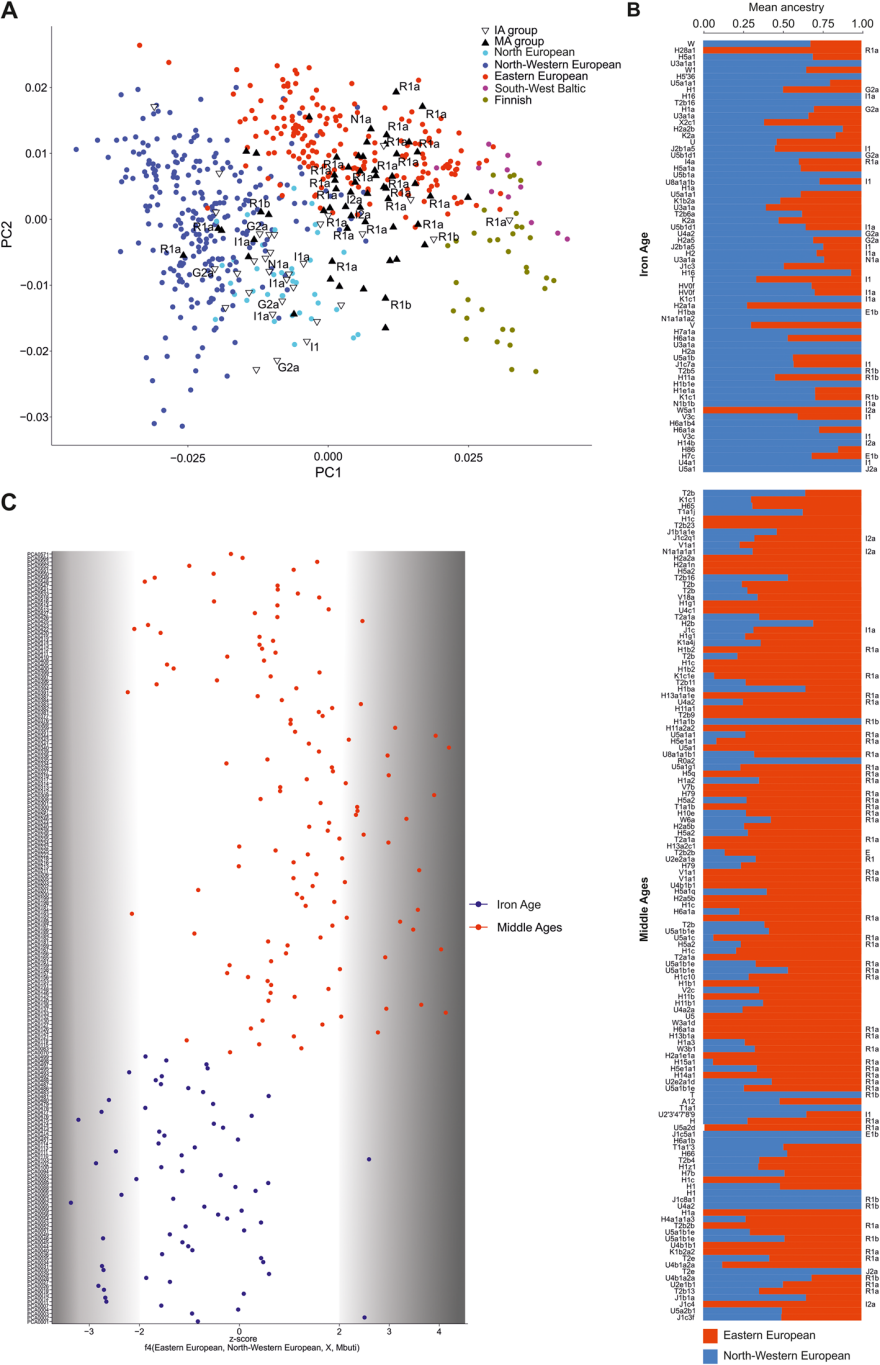
Population structure with respect to the present-day Central and Northern European genomes. **a** Ancient individuals projected onto the first two eigenvectors of the PCA restricted to the present-day Northwestern and East-Central Europeans. Northern European (Norway, Sweden), North-western European (England, Scotland, Orkney, Denmark), Eastern-European (Poland, Slovakia, Czech, Belarus, Ukraine), South-West Baltic (Lithuania, Latvia), Finnish (Finland). **b** f4 statistics reflecting the differential affinity of ancient individuals (*X*) to Northwestern and East-Central European reference populations, with the Mbuti as an outgroup. Colours denote the respective time periods of the ancient individuals: red—MA, blue—IA. **c** Northwestern (blue) and East-Central (red) European ancestry proportions estimated for ancient individuals using ADMIXTURE in supervised mode

To increase the resolution of the PCA, the genomic data obtained from the IA and MA individuals were compared with analogous data generated for the following 5 present-day East-Central European and Scandinavian populations: Eastern European, Northwestern European, Baltic, Finnish, and Norse (ancestry-restricted dataset) (Fig. 2a). The results also showed clear separation of populations from the IA and MA. The majority of IA individuals occupied the same PCA space as the Northwestern European and Norse populations, whereas the bulk of the MA individuals were placed together with the present-day East-Central Europeans, with some individuals located either close to the Norse populations or in the section of the PCA plot where Germanic and Slavic populations abutted each other.

Given that the ancestry profiles of most of the studied individuals were similar to that observed for the present-day Northwestern or East-Central Europeans, we used these contemporary populations as references in a supervised ADMIXTURE analysis of each IA and MA individual (*K* = 2) (Fig. 2b). IA individuals had the highest proportion of Northwestern European ancestry, and MA individuals had the highest proportion of East-Central European ancestry. The PCA and ADMIXTURE results were further supported by the f4 statistics in the form of f4(East-Central Europeans, Northwestern Europeans, X, Mbuti), where X denotes the studied ancient individuals from the IA and MA groups (Fig. 2c) (Additional file 2: Table S6). This test showed that the individuals from the IA and MA groups shared statistically significantly more alleles with present-day Northwestern and East-Central Europeans, respectively.

Although the exact phenotypes of the studied individuals are unknown, the estimated population-wide allele frequencies at loci associated with hair, skin, and eye colours [67] indicated that, on average, individuals in both groups were blond haired, light skinned, and blue eyed (Additional file 2: Table S2).

We performed analogous PCA and supervised ADMIXTURE analyses to determine the relationships between the studied populations and other ancient Europeans (for details, see Additional file 9: Fig. S20). The results demonstrated that the genetic ancestries of the IA individuals and MA individuals were similar and typical for post-Bronze Age Europe—three main genetic components common to all Europeans (WHGs, NAFs, and YAMs) contributed equally to the genomes of individuals from both studied groups (Additional file 9: Fig. S20B). The above findings indicate that the observed genetic differences between IA and MA populations resulted from local demographic processes that most likely took place during the IA.

### Genetic history of the IA group

The earlier analyses focusing on the IA and MA groups and their relations to present-day populations could not provide a clear answer which ancient populations contributed to the genetic makeup of the IA group. To explore this issue, we tried to identify within the IA group, the outliers that would represent the autochthonous IA population. Such individuals could be used to model the population that mixed with incomers associated with the Wielbark culture. Unfortunately, none of the methods we applied allowed us to identify the outliers.

To get deeper insight into the history of the IA group, we divided it into three subsets corresponding to the individual sites: IA_Pruszcz_Gdanski, IA_Kowalewko, IA_Maslomecz (Additional file 1: Supplementary Materials, Additional file 2: Table S1). We excluded from the analyses one site IA_Gaski. It was represented by two individuals described as associated with the Przeworsk culture. However, both were not buried in accordance with the customs of the Przeworsk culture and for both we obtained low-coverage sequencing data.

Based on the archaeological and historical findings, we expected the Wielbark culture-associated IA individuals to be the northern European IA immigrant population with the admixture of the autochthonous IA population.

To learn more on the origin of the immigrants, we calculated f4 statistics of the form f4(IA_X, MA, test population, Yoruba), where IA_X is IA_Pruszcz_Gdanski, IA_Kowalewko, or IA_Maslomecz, MA is the studied MA group, and test population is one of the 459 earlier identified ancient populations preceding or contemporary to the MA group (Additional file 1: Supplementary Materials, Additional file 2: Table S8). According to our expectations, IA_X, compared with MA, shared significantly more alleles with the ancient north (Denmark_IA, Norway_IA, Sweden_IA), north-western (England_Saxon, Ireland_Viking) Europe populations and with Hungary_Szolad_Migration_Period population (*z*-score > 3) (Additional file 2: Table S7).

We also used qpAdm to test if any ancient populations preceding or contemporary to the IA group could be a single ancestry source of the IA_X populations. In agreement with previous results, we found that for each IA_X population, valid one-way models (*p*-value > 0.05) can be generated with Denmark_IA, Sweden_LN or Scotland_Orkney_IAs as a source (Additional file 2: Table S8). Valid one-way models for at least two of the IA_X populations were also produced with Norway_IA, Sweden_BA or Scotland_Orkney_MBA. All three IA_X populations had also valid one-way models with Ukraine_Scythians as a source. This observation supports earlier reports that Ukraine_Scythians had close genetic affinities with the ancient northern populations (Sweden_IA, Sweden_LN, Denmark_LN) [68]. However, a model competition test (described in [69, 70]) showed that Denmark_IA is a better source of the ancestry for the IA_X populations than Ukraine_Scythians (Supplementary Information) which is consistent with archaeological data [32].

In addition, we applied qpAdm to test two-way quantitative models for the ancestral sources of each IA_X population. In accordance with the initial assumption, we considered models where the IA_X populations are mixtures of the north IA immigrants and the autochthonous IA population. As a source of the Norse ancestry, we used the earlier identified Denmark_IA or Norway_IA populations, and as a source of the local ancestry, we used one of the following populations: Poland_EBA_Unetice, Czech_EBA_Unetice, Lithuania_BA, Latvia_BA, or Estonia_IA (Additional file 2: Table S9). These populations are the closest approximations of the local IA genetic ancestry since no samples from the period from the BA until the IA are available for this region. We obtained multiple valid two-way models showing that IA_X populations are mixtures of Norse populations (~ 90–95%) and local proxies (5–10%). The models with Denmark_IA and Czech_EBA_Unetice were plausible for all three IA_X populations: IA_Pruszcz_Gdanski, IA_Kowalewko, and IA_Maslomecz (Additional file 2: Table S9). Several valid models were also produced for Denmark_IA + Poland_EBA_Unetice, Lithuania_BA, or Estonia_IA.

Both f4 and qpAdm models supported close genetic affinities between the IA_X populations and the contemporary to them and older northern European populations.

### Genetic continuation between the IA and MA

As we have already mentioned, the IA group was most likely composed of the northern European immigrant and the autochthonous IA population. The latter could contain the ancestors of the studied MA group (genetic continuation hypothesis) or could be genetically not related with the local medieval population (genetic discontinuation hypothesis). To verify these hypotheses of genetic continuity or discontinuity within the territory of contemporary Poland from the IA to medieval times, we examined whether and how the individual populations forming the MA group (MA_X populations; for details see Additional file 2: Table S11) are genetically shifted, compared with the studied IA group. To this end, we calculated the f4 statistics of the form f4(MA_X, IA, test population, Yoruba), where the test population was one of the earlier mentioned 459 ancient populations. The results showed that majority of the MA_X populations shared less alleles with ancient north-western populations (England_Saxons, Germany_Medieval, Norway_IA or Denmark_IA) than the IA group. At the same time, the MA_X populations shared more alleles with Latvia_BA, Estonia_BA and Lithuania_BA, compared with IA (Additional file 2: Table S13). This analysis revealed also that neither the MA nor the IA group was statistically significantly more shifted towards Poland EBA Unetice or Czech EBA Unetice, which are the best available proxies for the autochthonous IA population.

Further, we used qpAdm to test if the MA_X populations form valid (*p* > 0.05) one-way models with the IA_X populations. As shown in Additional file 2: Table S10, such models were formed by all MA_X populations with at least one IA_X population. All MA_X populations (12) formed valid one-way models with IA_Maslomecz, 10 with IA_Kowalewko and 9 with IA_Pruszcz_Gdanski. These analyses showed that the IA_X and MA_X populations were closely related and consequently supported a genetic continuation from the IA to MA.

Analogous one-way qpAdm modelling of the MA_X populations was also performed with 327 ancient populations preceding or contemporary to the MA group (Additional file 1: Supplementary Materials, Additional file 2: Table S10). It demonstrated that Denmark_IA, Norway_IA, England_Norfolk_Anglo Saxons, Ireland_Vikings, and Poland_Vikings also formed several valid models of the MA_X populations. These results indicated that the north European genetic components had strong presence in the studied MA group.

To further verify the hypothesis of genetic continuity, we used qpAdm to test two-way models of the MA_X population origin from the IA_X populations, Denmark_IA, or Norway_IA and the populations being proxies for the putative autochthons: Poland_EBA_Unetice, Czech_EBA_Unetice, or Lithuania_BA (Additional file 2: Table S12). These demonstrated that IA_Maslomecz + Poland_EBA_Unetice or Czech_EBA_Unetice resulted in valid models (*p* > 0.05) for all tested MA_X populations (8) with ~ 78–92% of the ancestry attributed to the IA_Maslomecz. IA_Kowalewko and IA_Pruszcz_Gdanski also produced valid two-way models but not for all MA_X populations (Additional file 2: Table S12). Most often IA_Kowalewko fitted with Poland_EBA_Unetice (3 out of 8) whereas IA_Pruszcz_Gdanski with Lithuania_BA (6 out of 8).

Two-way qpAdm models provided further evidence of a close genetic relationship between the IA and MA groups. This relatedness could be caused by the continuation of both the northern ancestry and the autochthon IA ancestry. However, none of the earlier analyses directly tested the autochthon IA continuation. If there was a genetic continuation of the autochthon IA population, then one should be able to model the IA_X populations as mixture of the MA X populations and Hungary_Szolad_Migration_Period who were shown to be incomers migrating from north through the region of contemporary Poland to south Europe. Indeed, we found that all IA_X populations could be modelled as mixtures of Hungary_Szolad_Migration_Period with the MA_X populations (Additional file 2: Table S14).

### Uniparental marker analysis

**Fig. 3:**
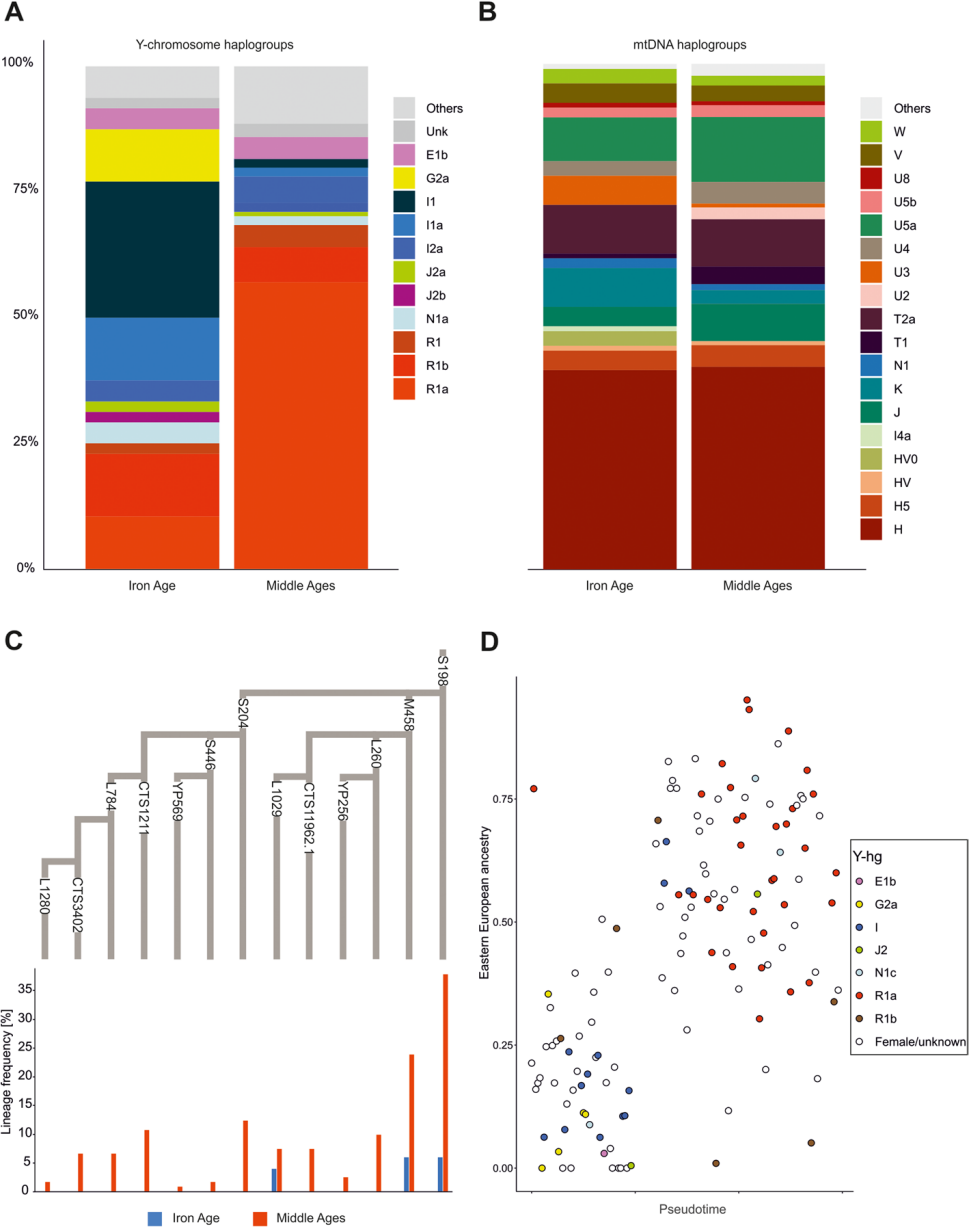
Genetic differentiation with respect to uniparental lineages. **a** Y-hg frequencies of the ancient individuals. **b** mtDNA frequencies of the ancient individuals. **c** A phylogenetic tree of the R1a-S198 lineage according to the ISOGG database v. 14.111, showing the frequencies of the respective SNP markers. Frequencies were based on the direct genotyping of the SNP markers, and the missing SNP markers were inferred using a phylogenetic context. **d** The proportion of East-Central European ancestry in ancient individuals. Colours indicate the Y-chromosome haplogroup for each male

To gain insight into the genetic structure of the studied populations from the perspective of male and female lineages, we determined uniparental haplogroups and compared their occurrence patterns in groups from both periods. The detected Y-hgs (155 in total) belonged to the following major branches of the Y-chromosome phylogenetic tree: E, F, G2a, I1, I2a, J2a, J2, N1a, R1a, and R1b (Fig. 3a, Additional file 2: Table S3). The Y-hg frequencies in the IA and MA groups were significantly different (Fisher’s exact test, *p* < 0.001). In the IA group, the Y-hg I1/I1a was the most frequent, whereas in the MA group, R1a predominated (Fig. 3c). The frequencies of both haplogroups underwent major changes between the two periods: the I1/I1a frequency decreased from 41.3% in the IA to 3.5% in the MA, whereas the R1a frequency increased from 8.6% in the IA to 57.5% in the MA. In the IA I1 individuals, the highest frequency was observed for the L118 mutation (41.3%), which is common in present-day Scandinavia and Finland. Among the four R1a individuals identified in the IA group, two were carriers of S198 and its downstream M458 mutations. In the MA group, 70.8% of R1a males bore the S198 mutation, and 44.6% bore the M458 mutation. The R1a-S198 lineage and its downstream clades, especially R1a-M458, are also common in present-day Eastern and Central European Slavic populations. In the MA group, we also observed high frequencies of R1a individuals carrying the S204 mutation (23.1%), which we did not detect in the IA group. The second most common Y-hg in the MA group was R1b with frequencies similar to that observed in the IA group. The R1b Y-hg was detected for individuals excavated from two elite-associated tenth century CE burials located in Poznan (at that time the city was one of the royal seats) and for one individual from Ląd, whose grave was dated to the thirteenth century and linked with the migration of peoples from Western Europe [71]. The Y-hg I1, most prevalent in the IA group, in the MA group was detected at the lower level (3.6%).

The genomes of IA individuals with the Y-hg R1a showed variable levels of the genetic component characteristic of contemporary East-Central Europeans (from 0 to 75%; Fig. 3d), whereas this component always exceeded 25% in the genomes of MA males with the Y-hg R1a. The genomes of IA individuals with the Y-hg I1 were similar to those observed in contemporary Northwestern Europeans, whereas the genomes of MA males with the Y-hg I1 were dominated by contemporary East-Central European components.

In contrast to the results of the Y-hg frequency analysis, we did not observe corresponding changes in the composition of matrilineal markers (mt-hg) between the IA and MA groups. The frequency of the dominant matrilineal lineage, mt-hg H, remained nearly constant in populations from both the IA and MA periods (43.2% and 44.4%, respectively) (Fig. 3b, Additional file 2: Table S4). Nonetheless, minor changes in the mt-hg distribution were observed, with frequencies of the mt-hgs U3a and K2a being slightly decreased in the MA group compared to the IA group. In addition, HV0, X, and I4a found in IA individuals were absent in MA individuals. However, all these mt-hgs were rare in both studied groups. Some changes were also observed within the mt-hg H distribution. The H5a2 and H5e1a lineages were found only in the MA group, and in this group, the frequencies of several H1c lineages were increased in comparison to those in the IA group. The same phenomenon was noted for several other mt-hg lineages, e.g., J, T1, T2a, U2e, U4a2, and W3. The largest increase in mt-hg frequency in the MA group compared to the IA group was observed for the U5a1b1e lineage. Moreover, in the IA group, the frequency of the mt-hg H in males with the Y-hg I1/I1a lineage reached 21% and was lower than the overall mt-hg H prevalence in the remaining individuals (44%).

In general, the observations concerning uniparental markers were consistent with the results of our whole-genome analysis and showed that the IA group was dominated by Y-hgs most frequently observed in ancient Northern European populations. Y-hgs dominating in present-day East-Central European populations were significantly less frequent but still appeared in this group. In contrast, the MA group was dominated by Y-hgs most frequent in present-day East-Central European populations. In addition, uniparental marker analyses indicated that the genetic changes induced in the region of contemporary Poland in the first centuries CE were based on male migration.

## Discussion

There are only a few reports on the genetic makeup of the populations inhabiting East-Central Europe in the first millennium CE. The majority of them are based on mitochondrial DNA [37, 38, 72, 73]. Whole-genome analyses of individuals living in this region in the IA and the early MA are scarce [1, 66, 74] and, for the region of contemporary Poland, very limited.

Presented here whole-genome analyses of the individuals from the IA group together with our previous observations [37, 38] and numerous archaeological findings consistently support earlier hypotheses assuming that the Wielbark culture was associated with immigrants from Northern Europe who spread within the region of present-day Poland and mixed with the autochthonous IA population. Most of the data collected for the IA and MA groups are in line with the hypothesis assuming genetic continuity from the IA to the early MA in East-Central Europe and suggest that the migration from east in the sixth CE was not necessary to form the genetic pool of the MA group. However, based on these data, one cannot exclude additional migrations from the Eastern Europe, either during the Migration Period or later.

Most of the collected data including f4 statistics and qpAdm models showed that the genetic makeup of the Wielbark culture-associated people was related with the ancient northern European populations including Denmark_IA and Norway_IA. This result is consistent with our earlier observation of matrilineal connections between IA_Kowalewko and the IA population from Jutland Peninsula [37]. Here, we further showed that the Wielbark culture-associated IA populations could not be derived from the preceding the EBA_Unetice populations alone. It means that the migration from northern Europe was necessary to the formation of the IA group genetic pool.

In addition, f4 statistics and qpAdm models revealed that the MA population could not be formed by people living earlier in the same region and approximated by Poland_EBA_Unetice, Czech_EBA_Unetice or Lithuania_BA. Putative northern ancestors of the Wielbark culture-associated people (approximated by Denmark_IA or Norway_IA) also could not explain the genetic makeup of all MA_X populations. The valid two-way models for all MA_X populations were obtained only for Poland_EBA_Unetice or Czech_EBA_Unetice and IA_Maslomecz. The latter population represents the north incomers with the highest admixture of the autochthon IA population since IA_Maslomecz was formed at the last stage of migration of the Wielbark Culture-associated peoples through the territory of contemporary Poland. It further means that the autochthonous IA genetic component was indispensable to model the whole MA group and that it is possible to explain the genetic makeup of the MA group without a need for separate migration from east in sixth CE or later. f4 statistics demonstrated also that neither the MA nor the IA group was statistically significantly more shifted towards Poland EBA Unetice or Czech EBA Unetice. This observation indicates that the latter two could be a common ancestral component for the IA and MA populations. However, qpAdm models showed that the EBA related ancestry in the IA and MA populations was minor.

The above results are consistent with the hypothesis assuming migration from north and genetic continuity in the region of contemporary Poland from the IA to the MA. Accordingly, we observed that majority of the ancestry of the MA populations could be derived from the preceding IA populations in particular IA_Maslomecz. The MA populations, compared with the IA populations, had lower genetic affinities to ancient northern Europeans and higher to ancient Eastern Europeans (see Additional file 2: Table S11). Still, the MA populations showed close relations with ancient north and north-western Europeans indicating the continuity of the northern European genetic component. That is why, to model some of the MA populations, Denmark_IA could be used as the only source. However, high genetic contribution of the IA populations to the MA populations suggests not only the continuation of the common north European ancestry but also genetic continuation of the autochthon IA population which mixed with the incomers. The possibility that the autochthon IA population could be closely related with the MA populations was additionally supported by modelling the IA populations as mixture of Hungary_Szolad_Migration_Period and the MA populations, where majority of the ancestry was derived from MA populations (~ 80%).

Our Y-chromosome analyses revealed significant differences between the two studied groups. The patrilineal genetic structure of the IA group members was dominated by I1 Y-hg lineages. Many identified I1 clades are now the most common Y-hg in present-day Nordic populations, reaching a 28–38% frequency [75, 76]. An increased I1 Y-hg frequency was previously reported in the context of Anglo-Saxon migrations to Britain [77–79]. This further supports the Northern European origin of the Wielbark culture-associated people. The Y-hg I1 frequency dropped significantly in the MA group in favour of the Y-hg R1a. The R1a-S198 marker exceeded a 40% frequency in the MA group, with R1a-M458 being the most common lineage. Today, both R1a-S198 and R1a-M458 are most frequent in the east-central Europe population [80]. We found that all IA group individuals with Y-hg R1a belonged to the R1a-M458 lineage. These results, together with the earlier report on R1a-S204 lineage detection in an individual associated with the Late Bronze Age Urnfield culture [26], strengthen the evidence that R1a-S204 Y-hg lineages, which are dominant in present-day East-Central European populations (Polish, Czech, Belarusian, Ukrainian) [80], were already present in East-Central Europe, at least since the Late Bronze period.

Matrilineal haplogroup analysis revealed another aspect of demographic processes that occurred in east-central Europe in the first millennium. The lack of significant differences between the patterns of mt-hg frequencies in the IA and MA groups shows that populations inhabiting East-Central Europe before and after the Migration Period had similar matrilineal genetic structures. This result is consistent with several earlier reports on the genetic continuity of mt-hg lineages in East-Central European populations between the IA and MA periods [72]. Although the overall pattern of mt-hg frequencies was roughly the same in both groups, in the MA group, we found several lineages very specific to present-day Eastern European populations (Ukrainian, Russian) [81] (H5a2, H5e1a, H1c, U4a2, and U5a1b1e), which were absent or present at very low frequencies in the IA group. The increases in the H5a2, H5e1a, and U5a1b1e mt-hgs suggest that after the Migration Period, there was at least some movement of peoples from East to Central Europe; however, this observation could also be explained by the smaller sample size of the IA group studied here.

Considering the above, one can speculate that the observed differences in the patterns of Y-hg frequencies at the beginning and end of the first millennium CE as well as the stability of the mt-hg frequency profiles are products of androcentric and paternalistic social structures, characteristic to this period. Usually, the men migrated and the women were local. This phenomenon may also explain the low incidence of Y-hg R1a in the IA group. There are many examples in history showing that a relatively small group of male immigrants can subjugate a local community (e.g. the history of the colonization of North and South America). Thus, a small number of individuals with Y-hg R1a in the IA group does not necessarily mean a low frequency of this haplogroup in the autochthonous IA population. It may, however, indicate a natural phenomenon of excluding local men from communities dominated by male immigrants.

Here, we provide several pieces of evidence that the ancestors of the medieval populations lived in the region of present-day Poland during the IA. There are, however, several aspects that need further elucidation. Firstly, how and when the ancestors of the MA populations with Y-hg R1a appeared. The times when Y-hg R1a-M417 dominated in this territory are associated with the spread of the Corded Ware culture (from 3000 to 2300 BC) [82]. Later, it was replaced by the Unetice culture (from 2300 to 1600 BC) [82] that was associated with the populations in which Y-hg R1a was very rare. From then until the IA, there were many archaeological cultures in this region from which no genetic data is available as cremation became the dominant burial practice. Here, we showed that the IA and MA populations inherited only a small percentage of genetic ancestry from the people associated with the Unetice culture. Therefore, the ancestors of the autochthonous IA populations with Y-hg R1a would have either had to be revived in the BA period or come from east during the BA or IA period. At this point, it should be noted that based on our results, one cannot explicitly rule out additional waves of migration after the IA. Thus, one of the reasons for the increase in the frequency of Y-hg R1a could also be migrations from Eastern Europe after the Migration Period.

Although they seem less likely one cannot exclude the alternative scenarios that do not assume the presence of the ancestors of the medieval populations in the region of contemporary Poland during the IA. One possibility is the numerous waves of migration from northern Europe in both IA and medieval times. According to this scenario, the medieval populations could have arisen as a result of the migration of individuals carrying genetic components similar to those possessed by the autochthonous IA populations. In this case, the immigrants would not be direct descendants of the autochthonous IA populations. Another scenario may assume the occurrence of a bottleneck effect that could make the IA and MA populations similar to each other under the conditions of reduced genetic diversity. There is also possibility that both the IA and medieval populations shared a common ancestral population that has not yet been studied. This common ancestry could explain the genetic similarities between the two populations without the direct presence of the ancestors of the medieval populations in the area of present-day Poland in the IA. Certainly, the basic limitation hindering the verification of the scenarios presented above is the lack of aDNA samples from people living in the period between the disappearance of the Unetice culture and the beginning of the IA and from people belonging to the Przeworsk culture.

## Methods

### Ancient DNA analysis

DNA extraction from skeletal samples and preparation of barcoded next-generation sequencing libraries were performed in a clean laboratory dedicated to ancient DNA analysis. All DNA samples were initially submitted to screening sequencing with the use of Illumina GAIIx or NextSeq 550 instruments to obtain 5–10 million 75-bp single reads per sample. The obtained sequences were mapped [83] to the human reference genome hg19 and the reconstructed mitochondrial DNA consensus sequence with the blocked seed (-l 1000) in bwa 0.7.10, and then duplicated sequences were removed using Picard Tools. DNA authenticity was evaluated by estimating the rate of mismatched sequences in the consensus mitochondrial sequence and by calculating the number of C > T and G > A changes near read ends with MapDamage 2.0 [84].

Libraries selected as the most promising as a result of screening sequencing were enriched in sequences overlapping the mitochondrial genome, Y chromosome, or whole human genome and sequenced with an Illumina HiSeq 4000 instrument and 100-bp-long paired-end reads. The data were processed as described above with additional trimming of 5 nucleotides from 5′ and 3’ ′ends. Then, the data from different libraries prepared with DNA of the same individual were merged. DNA authenticity was further evaluated by estimating X-chromosome contamination in males based on the rate of heterozygosity [85] and mtDNA-based contamination [86]. Samples with evidence of contamination were either filtered out or restricted to sequences with terminal cytosine deamination to remove sequences derived from modern contaminants. Finally, we filtered out samples with fewer than 10,000 targeted SNPs [87] and samples derived from individuals who were first-degree relatives of others present in our dataset [88] (samples with larger numbers of covered SNPs were retained) (for details, see Additional file 1: Supplementary Materials).

### Mitochondrial haplogroup determination

mtDNA haplogroups were assigned based on complete mtDNA sequences using HaploFind [89] with respect to PhyloTree build 17 (http://www.phylotree.org/) [90]. Only samples with a haplogroup score ≥ 0.8 and an average coverage ≥ 3 were used in downstream analyses.

### Y-chromosome haplogroup determination

The Y-chromosome haplogroup nomenclature of the International Society of Genetic Genealogy (ISOGG) database ver. 14.111 was applied. Haplogroup prediction was performed with Yleaf v2.1 [91] with the following parameters: -r 2, -q 30, -b 90 (Additional file 1: Supplementary Materials).

### Merging newly generated data with published data

For the comparative genome-wide analyses, several publicly available datasets were applied. The first dataset contained genomes of 983 present-day West Eurasians genotyped with the Human Origins Array (HO dataset). The second dataset, consisting of 694 human genomes, was extracted from the Population Reference Sample (POPRES) project [92] by random selection of up to 100 individuals per European population (POPRES dataset). For the third dataset, we combined genomic data of individuals from present-day Gaelic, Germanic, Slavic, Baltic, and Norse populations from the HO and POPRES datasets and 296 individuals speaking Balto-Slavic languages genotyped by Kushniarevich et al. [93] (ancestry-restricted dataset). For the f4 statistics and qpAdm models [94], we used the comprehensive dataset 1240 K AADR v54.1 [95].

For each ancient and present-day individual, each heterozygous SNP was assigned as pseudohaploid by randomly sampling one of two alleles detected at a particular position.

### Principal component analysis

PCA was performed on the HO, POPRES, and ancestry-restricted datasets using the ‘smartpca’ program in EIGENSOFT [96]. PCA was computed using present-day individuals, and then the ancient individuals were projected into a PCA plot using lsqproject:YES and shrinkmode:YES.

### ADMIXTURE analysis

Model-based clustering analysis was performed using unsupervised ADMIXTURE [97] on the HO reference dataset, which included genomic data of 968 present-day individuals from West Eurasia and Africa and ancient individuals. First, we carried out linkage disequilibrium pruning of the data using PLINK (–indep-pairwise 200 25 0.4), leaving 163,222 SNPs. ADMIXTURE was run with the cross-validation (–cv.) flag specifying from *K* = 2 to *K* = 10 clusters, with 20 replicates for each *K* value. For each *K* value, the replicate with the highest log likelihood was retained. Finally, we chose the *K* = 9 value, as it was the lowest value for which components of ancestry related to Northwestern and East-Central Europeans were maximized. Supervised ADMIXTURE was run on the dataset restricted to the present-day Northwestern and East-Central Europeans.

### f-Statistics

f-Statistics were computed using the HO dataset (present-day populations) or AADR v54.1 1240 panel (ancient populations) in ADMIXTOOLS with default parameters. We used qpDstat with f4mode:Yes for f4 statistics. Standard errors were calculated using a weighted block jackknife over 5-Mb blocks.

### qpAdm

One-way and two-way qpAdm modelling was done for each IA and MA sites separately. As left populations, we used West Eurasian ancient populations preceding or contemporary to the IA and MA groups. All models were tested under the same set of right outgroup populations consisting 17 ancient populations previously described by [70]. For details, see Additional file 1: Supplementary Materials.

## Supplementary Information

**Additional file 1.** Supplementary Figs. S1-13 and Supplementary Materials.

**Additional file 2.** Supplementary Tables.

**Additional file 3: Fig. S14.** PCA embedding of the studied ancient individuals and present-day West-Eurasians. Ancient individuals were projected onto the first two eigenvectors of a PCA based on contemporary West Eurasians.

**Additional file 4: Fig. S15.** Genetic distances between studied ancient individuals and present day West Eurasians. Colour coding reflects the genetic distance as measured by the Fst coefficient between ancient individuals and present-day ones separately for the IA and MA group.

**Additional file 5: Fig. S16.** Estimated Eastern-European admixture proportions in ancient populations. Shown is the result of unsupervised ADMIXTURE run at K=9 corresponding to the genetic components presented on Fig. 1D and fig. S17.

**Additional file 6: Fig. S17.** Estimated ancestry proportions for present-day populations from Human Origins dataset. Shown are unsupervised ADMIXTURE results for K=9.

**Additional file 7: Fig. S18.** Geographical distribution of Eastern-European admixture in present-day European populations. Shown is the result of unsupervised ADMIXTURE run at K=9 corresponding to the genetic components presented on Fig. 1D.

**Additional file 8: Fig. S19.** Geographical distribution of North-Western admixture in present-day European populations. Shown is the result of unsupervised ADMIXTURE run at K=9 corresponding to the genetic components presented on Fig. 1D.

**Additional file 9: Fig. S20.** Spatiotemporal distribution of samples and their genetic affinities to ancient populations. (a), PCA embedding of the studied samples and other ancient post-LN samples from Western Eurasia. Polygons highlight the space occupied by the samples from each group. (b), supervised ADMIXTURE (K=3) for IA and MA individuals modelled as mixtures of Neolithic Anatolian Farmers (orange), Western Hunter-Gatherers (blue) and Yamnaya (green).

**Additional file 10.** Author and affiliation list for members of the Polish Archaeogenomic Consortium.

**Additional file 11.** Peer review history.

## Data Availability

No novel software tools were developed for the purpose of this work. DNA sequence alignments from individuals sequenced in this work are available at the European Nucleotide Archive (http://www.ebi.ac.uk/ena) under accession number PRJEB48333. The used public datasets consisted the genomes of 983 present-day West Eurasians genotyped with the Human Origins Array (HO dataset) [94]. The dataset, consisting of 694 human genomes, was extracted from the Population Reference Sample (POPRES) project [92]. The genomic data for the 296 individuals speaking Balto-Slavic languages was acquired from Kushniarevich et al. [93] (ancestry-restricted dataset). The dataset consisting of ancient individuals was acquired from the AADR v54.1 database [95].
