# Supplementary material for: Genetic history of East-Central Europe in the first millennium CE
Source: Genome Biol. 2023 Jul 24;24:173. doi: 10.1186/s13059-023-03013-9 (PMC10364380; doi:10.1186/s13059-023-03013-9)
Supplement: Supplementary file 1 — Additional file 1. Supplementary Figs. S1-13 and Supplementary Materials. [file 13059_2023_3013_MOESM1_ESM.pdf]

Supplementary Materials

Genetic history of East-Central Europe in the first millennium CE

Ireneusz Stolarek[1], Michal Zenczak[1], Luiza Handschuh[1], Anna Juras[2], Malgorzata Marcinkowska-Swojak[1], Anna Spinek[3], Artur Dębski[4], Marzena Matla[5], Hanna Kóčka-Krenz[4], Janusz Piontek[2], Polish Archaeogenomics Consortium Team[#], Marek Figlerowicz[1]*

[1]Institute of Bioorganic Chemistry, Polish Academy of Sciences, Poznan, Poland

[2]Institute of Human Biology & Evolution, Faculty of Biology, Adam Mickiewicz University, Poznan, Poland

[3]Institute of Immunology and Experimental Therapy, Polish Academy of Sciences, Wroclaw, Poland

[4]Faculty of Archaeology, Collegium Historicum, Adam Mickiewicz University, Poznan, Poland

[5]Faculty of History, Collegium Historicum, Adam Mickiewicz University, Poznan, Poland

# - Members of Polish Archagenomics Consortium Team with affiliations are listed in Additional file 10

* Correspondence and requests for materials should be addressed to:
Prof. Marek Figlerowicz
Institute of Bioorganic Chemistry
Polish Academy of Sciences
Noskowskiego 12/14
61-704 Poznan, Poland
email: marek.figlerowicz@ibch.poznan.pl

SUPPLEMENTARY NOTE 1. BRIEF BIOLOGICAL HISTORY OF THE CENTRAL-EAST EUROPE

For thousands of years, the plains of Central-east Europe were a melting pot of all major genetic ancestries that make up present-day Europeans gene pool. The changes in genetic structure of Europeans were caused by several mass human migrations and catastrophic natural events. Judging from the data collected for the individuals found in Siberia and Romania, the first human settlers of Europe were Paleolithic Hunter-Gatherers, dated to live at 45-40,000 years ago. At around 39,000 years ago a catastrophic volcanic eruption almost ended their existence and led to the extinction of European Neanderthals. Europe became resettled by a surviving group of Paleolithic Hunter-Gatherers linked with the Aurignacian material culture. This population was more closely related with present-day Europeans than East Asians, however, with no particular affinities to any present-day European population. Eventually, they became replaced by a population of Paleolithic Hunter-Gatherers linked with a distinct Gravettian material culture, at 34,000 - 26,000 years ago. This genetic structure survived for 11,000 years until the Last Glacial Maximum (LGM). After the LGM, a new material culture, called Magdalenian, spread from the Iberian Peninsula. The people associated with it were not direct descendants of the Hunter-Gatherers from the Gravettian culture but also with predating them people associated with the Aurignacian culture. It is likely that the Hunter-Gatherer populations survived this period in refugial areas located in the Western Europe. At around 14,000 years ago a major heat wave reached Europe. With it a new Hunter-Gatherer population with affinities to the Near Eastern populations entered Europe. As a result of these events major groups of Hunter-Gatherers formed in Europe: Western Hunter-Gatherers, Caucasus Hunter-Gatherers, Scandinavian Hunter-Gatherers and Eastern Hunter-Gatherers. With these being distinct groups in terms of genetic ancestry, in Central-East Europe, Hunter-Gatherer populations with ancestry not composed entirely of one of these groups existed. This suggests that some populations, like Baltic and Ukrainian Hunter-Gatherers, might have been a previously unsampled component of a Hunter-Gatherer meta-population that stretched across Central-Eastern Europe during the early Holocene. Since 7,000 years ago a Neolithic revolution reached Europe. This event was not homogenous in its nature across the whole region. Changes brought about migration of people associated with Anatolian farmers ancestry happened in sub-Carpathian region, Pannonia and eastern Germany, reaching the lakeland regions of Poland. Eastern parts of the present-day Ukraine and baltic states (Lithuania, Latvia and Estonia) experienced Neolithic revolution in a way of adoption of the farming practices by the endogenous groups of Hunter-Gatherers. Northern parts of Poland remained Hunter-Gatherer-like in both ancestry and customs until the Middle Neolithic, with the Kuyavian region serving as a genetic border between the two worlds.

The Late Neolithic Period was a time of another genetic turnover in Eastern and Central Europe. During this time around 5,000 years ago a new type of ancestry reached Europe. It was linked with the Yamnaya pastoralists living in the Pontic-Caspian steppe. Yamnaya-related ancestry did not, however, reach Central Europe in a uniform manner. It was present in high amounts in people associated with the Corded Ware Culture, but absent in people associated with the Globular Amphora Culture. With the advent of the Bronze and Iron Ages, the dominant burial practice became the cremation of the dead which makes the studies of ancient DNA more challenging. This funeral rite persisted until the Roman Iron Age, when a new archaeological formation called Wielbark culture appeared in Central Europe. People associated with Wielbark culture practiced inhumation living next to, and even sharing cemeteries with people associated with the Przeworsk culture, which still mainly practiced cremation.

SUPPLEMENTARY NOTE 2. ARCHAEOLOGICAL CONTEXT OF CEMETERIES

1. Roman Iron Age

Czarnówko

Czarnówko is a small village situated in the valley of the Łeba River, a few kilometers from Lębork (Pomeranian voivodeship). Archaeological research began here in 1972 and continued in 1973-1975, 1980-1990, 1996-1997, 1998-2000, and 2008-2015. At that time, a total of 2,400 graves (skeletal, less often cremated) were identified, in the vast majority of the Wielbark Culture graves. At the same site, a cemetery of the people of the Oksywie culture was discovered, as well as much smaller necropolises dating back to the periods of the Pomeranian culture, Migration Period, and the early Middle Ages. Also, single graves from the Late Stone Age and the Early Bronze Age have been recorded. The chronology of most tombs covers the period between the A2 phase of the younger pre-Roman period and the C1b phase of the Roman period (from the end of the 2nd century BCE to the middle of the 3rd century CE). The furnishing of the graves was considerably diverse: discovered burials were both devoid of grave gifts and very rich, sometimes referred to as "princely", with numerous utility items and decorations, including those made of semi-precious stones, silver, and gold.

[There is no cemetery plan available]

Gąski

The archaeological site of Gąski 18 (Kuyavian-Pomeranian voivodeship) is located on a small hill in the Parchani River valley, next to the old watercourse (now canalized). It was discovered in 1973 during the planned inventory of settlement spots. Excavations were carried out between 1984 and 1991, during which the area of 13 ares was recognized and, inter alia, a graveyard of the community of the Kruszów group of Przeworsk culture was discovered. On the 8 ares necropolis, 122 cremation and 39 skeletal burials, and 31 other objects were uncovered, including symbolic burials and traces of rituals. The skeletal burials were oriented along the north-south axis. In the graves with the goods as weapons, tools, ornaments, and clothing were found. In the center of the cemetery, there was an approx. 3 m^2 structure made of stone and clay, located on the top of the hill. In the south-eastern part of the burial ground, an empty square adjacent to a cluster of human skulls was discovered. The chronology of the cemetery was established from the 2nd century BCE. until the 1st century CE.

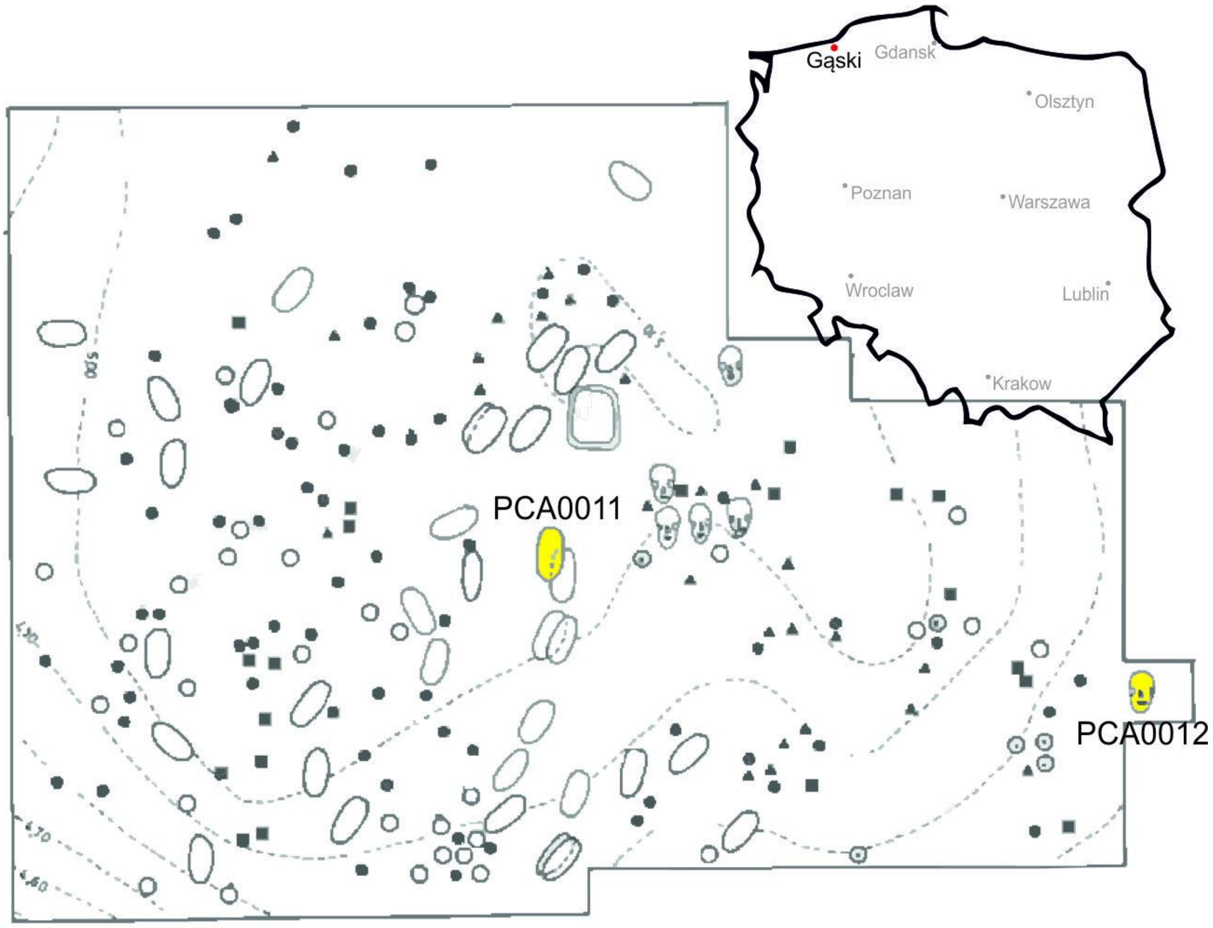

Fig. S1: Location and plan of the Gąski archaeological site. [based on Figure 2 from Andrałojć M, Stolpiak B; modified using Corel Draw X6]

Kowalewko

Kowalewko is a village in Greater Poland voivodship, near Poznan, in the middle reaches of the Samica Kierska River. Biritual Roman Age cemetery (site 12), dated from the mid-1st to the beginning of 3rd century CE, is located in the featureless arable fields at the south and west of the village. It is one of the most significant and valuable burial sites identified with the Wielbark Culture in Poland. The "untouched" nature of the cemetery and favorable burial environment enabled the survival of bones and artefacts in good condition. As a result of excavations, conducted in 1995-1998, 496 graves (including cremation burials and inhumation graves) were discovered, including 5 under burial mounds. The graves, representing the plenty of burial forms typical for the Wielbark Culture, were classified to the subphases B1b to C1a, based on the type of the burial rite, the body position, the found goods and the grave constructions. The furnishing of the graves includes dress accessories, ornaments and everyday objects such as pottery vessels, combs or spindle whorls, but specialist tools and weapons were not observed. The grave goods indicate cultural contacts of the Kowalewko community with the Elbian circle and the Danish islands.
Our earlier studies, based on mtDNA analysis of the population buried in Kowalewko were published in Scientific Reports.

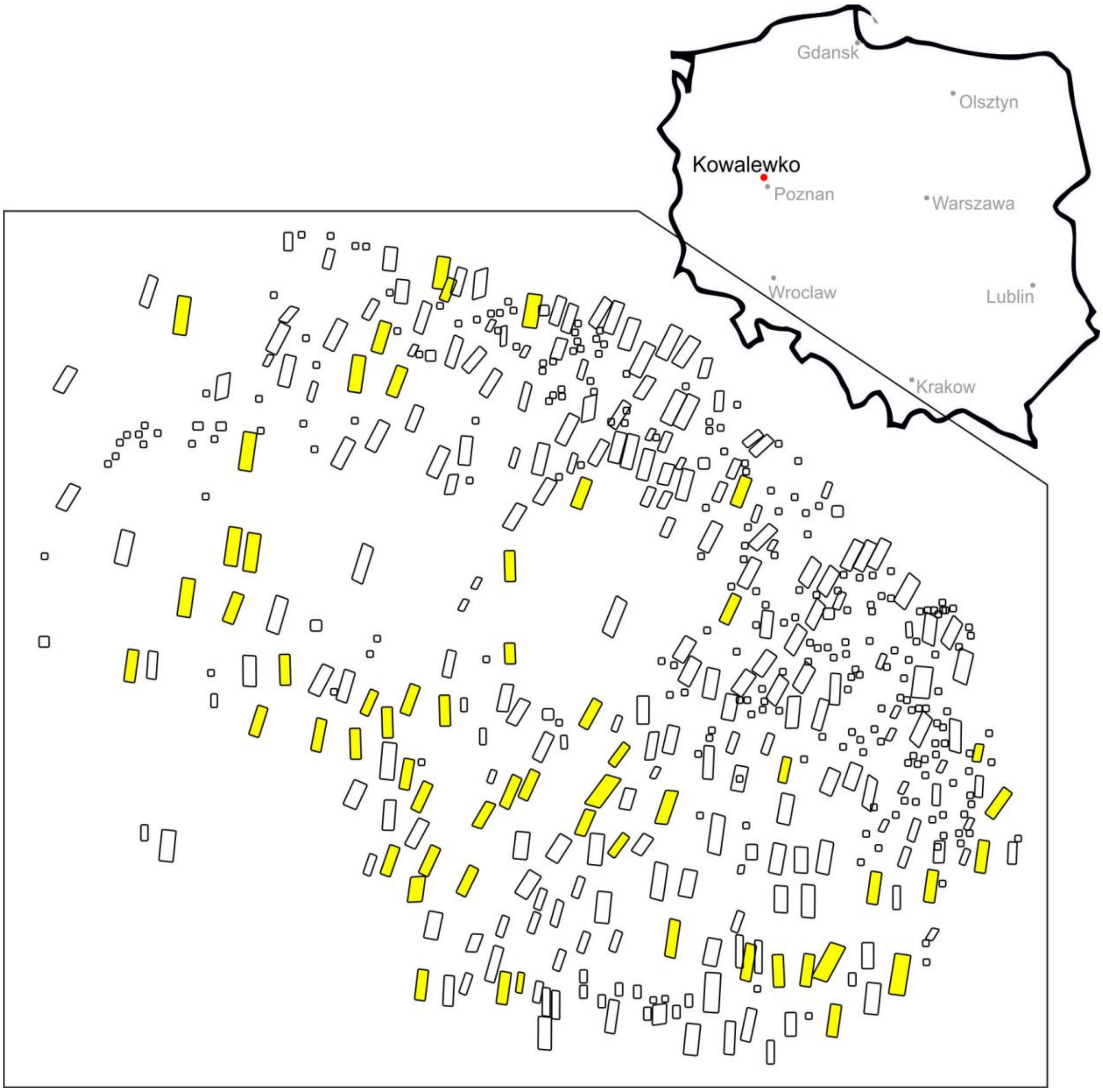

Fig. S2: Location and plan of the Kowalewko archaeological site. [based on Supplementary Figure S1 from Stolarek et al; modified using Corel Draw X6]

Masłomęcz

A complex of Goth settlements and cemeteries from the Roman period (end of the 2nd century - end of the 4th century CE) was located in Maslomecz (Lubelskie voivodeship), and the research in this town gave the basis for the distinction of the "Masłomęcka group". The discovery was made in 1977. The settlement (site 8-9) is considered to be the largest in the Hrubieszowska Valley and could have acted as the "capital" of the Masłomęcka group. The most important, however, is the completed excavations (1978-2002) in the cemetery at site 15 (Masuria Field), which was built on a diamond plan with the corners facing the directions of the world. In its center, there was a square free from graves, with a "sacred well" and "body storage". The tombs were arranged on the west side along the northwest-southeast axes, on the east side on the curves going from the west to the north-east. The facility was probably encircled in the past. At the end of Antiquity (mid-4th century CE), a cremation layered cemetery was established on a circular plan with a diameter of 13 m, with a small pillar structure standing in it. 536 graves were discovered including human skeleton and cremation, and animal graves. It

was found that the graves were opened many times for ritual purposes. The dead were buried in burial chambers, in log and box coffins, in ritual boats, and directly in the ground. As a standard, the graves contained vessels, ornaments, garments, and combs. The most famous findings are: brooches with a bow in the shape of ducks, a golden plate with the image of a man's face, Roman glass cups, an amulet made of a human femur, and a necklace with a golden clasp.
Our earlier studies, based on mtDNA analysis of the population buried in Masłomęcz were published in several reports.

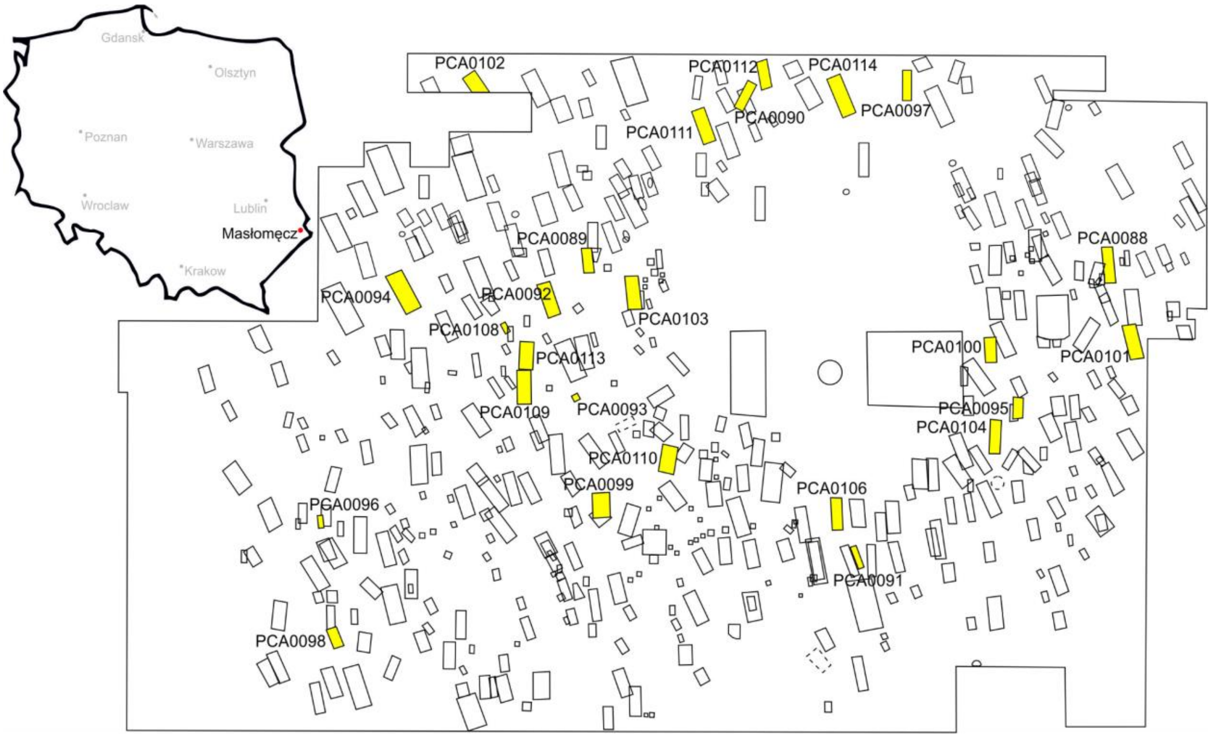

Fig. S3: Location and plan of the Masłomęcz archaeological site. [based on Supplementary Figure S1a from Stolarek et al; modified using Corel Draw X6]

Pruszcz Gdański

In the area of today's Pruszcz Gdański (Pomeranian voivodeship), 6 cemeteries of the population of the Oksywie and Wielbark cultures have been registered so far. One of them is the necropolis marked as site 5, located at today's Wit Stwosz street. The cemetery is now located between the Radunia River and the Radunia Channel, in a strongly wet area, full of small reservoirs and watercourses. The first discoveries at the site were made in the years 1926 and 1929-1930, and a total of 18 graves were registered then. Rescue excavations were carried out in 1992, 1995-1996, 2007, and 2009. In the end, at least 66 graves and an indefinite number of objects destroyed during various earthworks were registered in the cemetery. Skeletal burials dominated, less often cremation, often equipped very richly with utility items, ornaments, and even single military items, e.g. spurs. The cemetery in Pruszcz Gdański dates back to times from the late Roman period to the Migration Period, i.e. from the middle of the 2nd century until the 5th century CE.

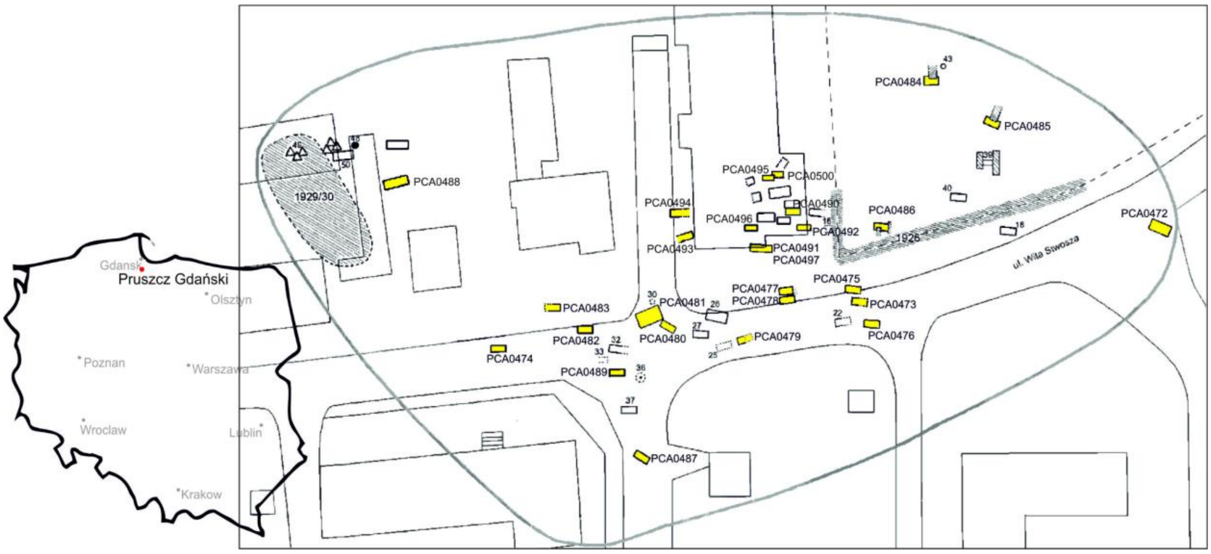

Fig. S4: Location and plan of the Pruszcz Gdański archaeological site. [based on Figure 2 from Pietrzak M, Cymek L, Rożnowski F; modified using Corel Draw X6]

2. Middle Ages

Balczewo

Mysterious necropolis in Balczewo (Kuyavian-Pomeranian voivodeship) was discovered at the turn of 1892/1893. During the extraction of sand from a small hill called the Windmill Mountain, 3 skeletons, a loose skull, and loose long bones were exposed. The dead were laid out in two tiers. In the top one, a bronze pin and 2 fragments of epaulets, suprisingly dated to the 2nd period of the Bronze Age, were discovered. The lower skeletons did not possess any goods, but traces of trepanation were noted on the skull of one of them. According to the archival documentation, about a dozen skeletons were to be discovered in the cemetery, but the collection of the Archaeological Museum in Poznań includes 3 skulls and the remains of a fourth, also bearing traces of trepanation, which are to come all from the above-mentioned site. Some of the graves had stone structures, perhaps guards. Absolute carbon 14C dating has shown the early medieval chronology of all surviving bone material.

[There is no cemetery plan available]

Dziekanowice

The cemetery known in the literature as Dziekanowice site 22 (Greater Poland voivodeship) is situated on a small hill, on the eastern shore of Lake Lednica, near the eastern bridge leading to the castle island. The extensive necropolis is dated from the end of the 10th to the end of the 13th century. It was discovered in 1964, and systematic excavations, intensified in 1991, were continued until 2013. A total of 1665 skeletal graves were examined, distinguishing the remains of 1730 individuals of various age and sex, often with pathological marks visible on the skeletons. A significant percentage of the burials contained equipment, often very rich, in the form of numerous ornaments, coins, utility items (utensils, spools) or items related to rituals. Particularly noteworthy is the group of probably the oldest graves, known as chamber graves,

distinguished by their size, construction, and the use of wooden coffins with iron fittings. In some of the other burials there were also traces of the probably wooden structures.

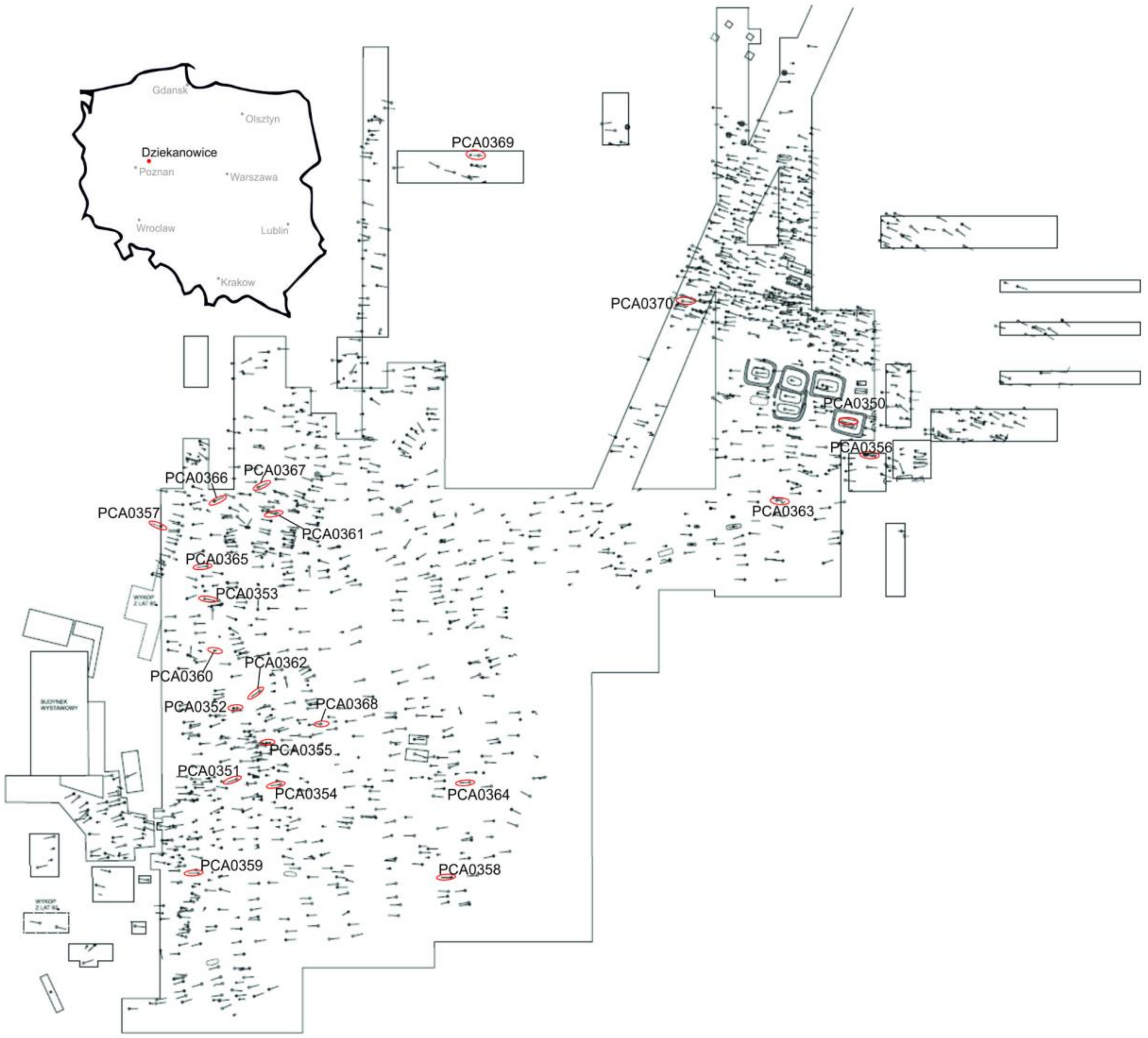

Fig. S5: Location and plan of the Dziekanowice archaeological site. [based on Plan no. 3 from Nummus bonum fragile est : graves with coins from the Early Medieval cemetery at Dziekanowice [Nummus bonum fragile est : groby z monetami wczesnośredniowiecznego cmentarzyska w Dziekanowicach] ed. J. Wrzesiński, Lednica 2016

Gniezno

Two graves from Gniezno (Kuyavian-Pomeranian voievodeship) were analyzed.
Grave no. 6/2010 (site 15b) was discovered in 2010 on the Lech Mountain, within the so-called first borough of the early medieval central stronghold, one of the most important centers of the Piast state power. The burial was discovered near the cathedral, 0.8 m north of the foundations of the Potocki Chapel, at a depth of approx. 0.9 m from the present surface of the ground, under the layer with the remains of the medieval cathedral cemetery. The well-preserved skeleton of an adult male, devoid of a skull, rested in a rectangular tomb made of split stone slabs laid without mortar. The lower chest had dimensions of 3.05 m x 1.1 m and was situated along the east-west axis. It was covered with a split sandstone structure, creating an apparent vault and

originally protruding above the ground level. The tomb, made in the pre-Romanesque technique, was dated to the first half of the 11th century.
Grave no. 1 (site 5) was discovered in 1976 in the area of the so-called second borough of the Gniezno stronghold, within the cemetery located south of the alleged sacred building. It was child's skeleton, rested in a wooden structure, probably a coffin, and dated to the first half of the 11th century. The burial was discovered in the vicinity of another, boneless, grave, approx. 2.5 meters south-east of the sacred building. Both graves were dug into the embankment of the early medieval hillfort and situated along the east-west axis.

[There is no cemetery plan available]

Gołuń

The necropolis in Gołuń (site 23/24, Greater Poland voivodeship) was discovered in 2007, during the exploitation of aggregate for the purpose of the Poznań bypass construction. At that time, bone material from five destroyed graves was recorded. In 2011, excavations were carried out, during which the entire area of the skeletal cemetery was identified, registering 44 graves, another 5 objects considered empty graves, and loose bone remains from another 10 individuals. The site is located on a small elevation to the south-east of a small watercourse. In the central part of the site, there is a complex of graves distinguished by rich furnishings. No traces of wooden structures were recorded in the burials, however, objects related to rituals, including traces of fires, were observed. Some of the graves were richly furnished, numerous ornaments (rings, beads), tools and elements of armament (ax and spurs) were noted. Based on the archaeological material and radiocarbon dating, the cemetery's chronology was determined at the end of the 10th and the first half of the 11th century.

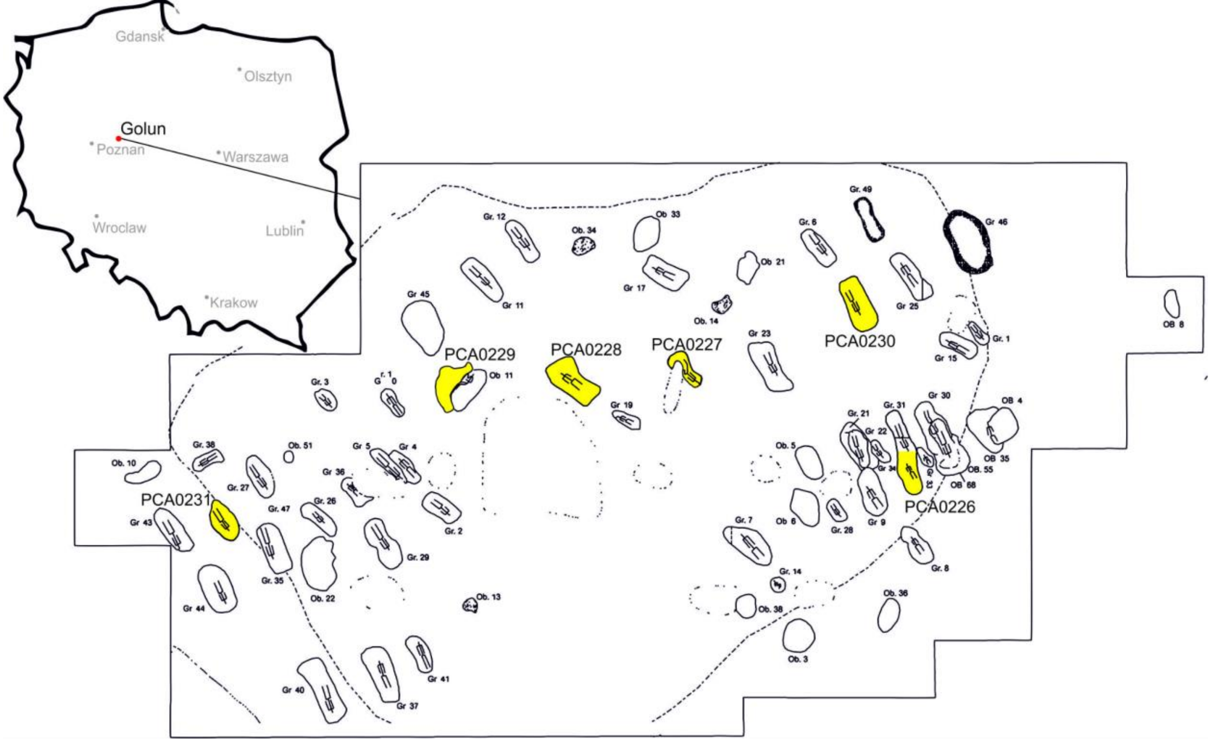

Fig. S6: Location and plan of the Gołuń archaeological site. [based on the Figure 5 from Andrałojć M; modified using Corel Draw X6]

Groszowice

The early medieval cemetery in Groszowice (now part of Opole, Opole voivodeship) was discovered in 1957 during the mining of limestone rocks. About 20 graves were destroyed then. The same year, rescue research was carried out at the site, which resulted in another 38 skeletal graves being uncovered.
The row cemetery was located on a small hill made of sand and limestone, located a short distance to the north of Groszowice. The dead were buried in oval (often) or square (seldom) pits with flat bottoms and of depth ranging from 0.1 m to 0.35 m. The skeletons rested on their back with the arms stretched out along the side of the body. All graves were situated along an east-west axis, with most of the dead facing west. Relatively rich equipment was discovered in the cemetery. In as many as 13 burials various items, rarely found individually, were recorded. These were ornaments (temple rings; ceramic, metal, amber, and glass beads, including entire necklaces; rings; kaptorga; bell) and everyday objects (knives, flints, and a wooden bucket). The chronology of the cemetery was set at the 10th and 11th centuries.

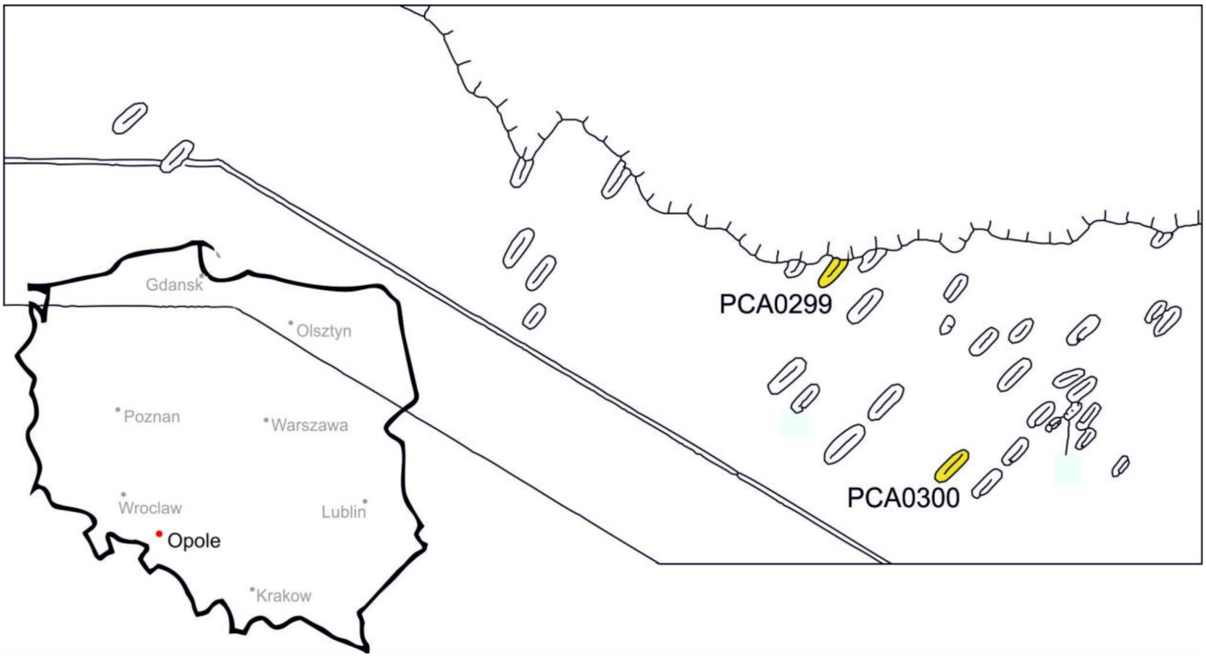

Fig. S7: Location and plan of the Groszowice archaeological site. [based on Figure 17 from Wachowski K; modified using Corel Draw X6]

Końskie

Early medieval necropolis in Końskie (Świętokrzyskie voivodeship) was discovered on a large gravelly elevation that is part of the terminal moraine formed during the Middle Polish Glaciation. In 1925, as a result of leveling the hill, 171 skeletal and –9 probably cremation burials were discovered and explored.
The dead rested on the backs with their hands along the body. In one case, the skeleton was slightly tilted to the right side. The burial pits were 1.5 m deep. The vast majority of burials (112 pieces) had stone structures in the form of enclosures covering the skeleton. In some of the pits, traces of fires were also observed, but, similarly to the cremation burials, they were only observed in the north-eastern part of the necropolis. The cemetery was richly furnished: in the graves recorded ornaments (temple rings, rings, glass and metal beads), utility items (knives, whetstones, flints, ring pins, buckets, ceramic vessels), clothing items (horseshoe-shaped clasps, buckles, including lyre) and weapons (swords, axes, spears, arrowheads, and spurs).

There were also coins in several graves. The cemetery was dated from the second half of the 11th century to the early 12th century.

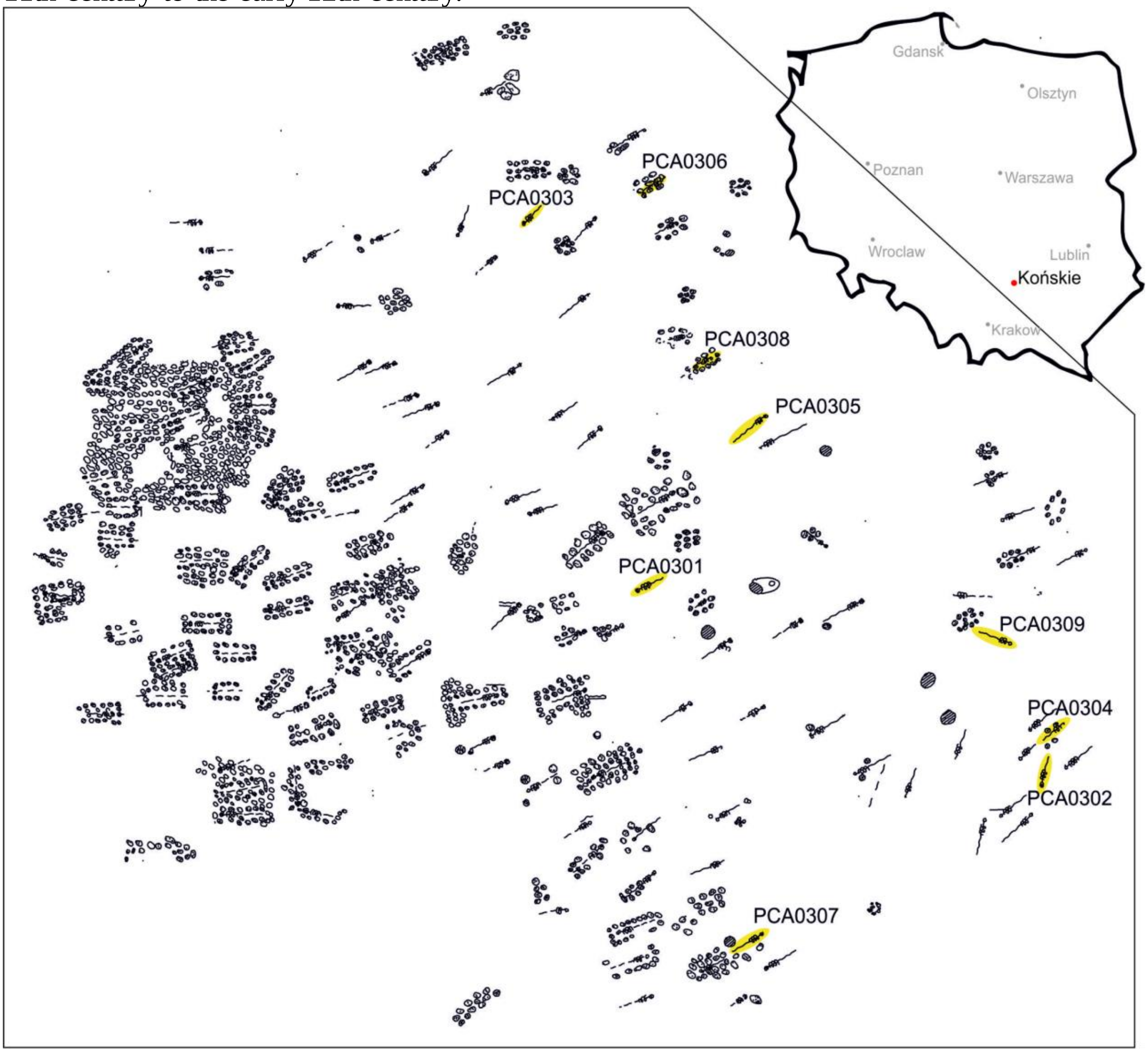

Fig. S8: Location and plan of the Końskie archaeological site. [based on Supplementary Figure 10 from Gąssowski J; modified using Corel Draw X6]

Kraków - Plac Matejki

During the excavation works in the courtyard of the Academy of Fine Arts in Krakow, an intentional burial of two individuals was found, below the remains of medieval wooden buildings. The skeletons were arranged along the east-west axis. The head of the *southern* individual was directed to the east and the *north* one to the west. The *northern* skeleton was cut with an erected wall, therefore it has only been partially preserved from the pelvis downwards. The *southern* skeleton was almost completely preserved. No archaeological artifacts helpful in dating were found within the burial site, therefore a fragment of the cortical layer of the femoral shaft was collected and radiocarbon dated (ca. 890-1010 CE). The individuals were buried on the side, which could be evidence of a non-Christian funeral rite.

The results of the research have not yet been published.

[There is no site plan available]

Ląd

Excavations in an early medieval stronghold in Ląd (Greater Poland voivodeship) began in 1960, were continued until 1964, and then with breaks in 1969-1986. The site is located in the area of the Middle Warta River Valley, among wet meadows, a short distance to the south-east of Ląd. Already in the first year of research, a burial ground was discovered in the highest part of the stronghold, and 10 years later a necropolis was discovered in the borough, which is located a little further to the west. Both were considered as church cemeteries and connected with two churches known from written sources: St. Andrew's and St. Peter's. The stronghold necropolis is dated from the end of the 11th to the middle of the 13th century, while the one in the borough is younger and was probably used between the mid-12th and mid-13th century.
In the stronghold cemetery, 196 graves were discovered, covering an area of approx. 400 m^2, i.e. half of its estimated area. The necropolis in the borough was probably 200 m^2, but only 130 m^2 was examined, revealing 131 graves. The dead were often placed on their backs, along the east-west axis with their heads to the west. The burial pits were from 0.25 m to even 1.7 m deep, and only in a few cases, exclusively in the stronghold, traces of wooden structures were observed. In the stronghold cemetery, 15.8% of the burials contained furnishings, while in the borough there were only 6.3% of such graves. The dead were usually buried with ornaments (temple rings and rings) or utility items (knives, single spindles, buckles, or pliers).

[There is no cemetery plan available]

Łęgowo

Early medieval cemetery in Łęgowo (Greater Poland voivodeship) was discovered during the renovation of the road No. 196 connecting the cities of Poznań and Wągrowiec. The necropolis (site 4) is located approx. 200 m east of the Łęgowskie Lake, along its northern part. Excavations on the site were carried out in 2013 and 2015, revealing a total of 28 skeletal graves, dated to the second half of the 10th and the first half of the 11th century. The number of goods in the graves was relatively modest and was usually limited to a few temple rings (in the case of women) and knives (in the case of men). A single grave with traces of a wooden structure and another one in which a ceramic flask was discovered were noteworthy. Both of these elements can be traces of foreign inspiration in the funeral rite. In a single case, 2 graves of children lying directly one above the other were registered. The other burials did not overlap, which indicates that the burial ground probably functioned for a short period of time.

The results of the research have not yet been published.
[There is no cemetery plan available]

Markowice

Most of the analyzed samples from Markowice (Kuyavian-Pomeranian voivodeship) were from site 12. Only one was from site 9.

An early-medieval cemetery located in Markowice (site 12), was discovered and studied in 2014-2015. At that time, 243 graves containing the remains of 248 individuals were registered. There were no skeletons in 3 cavities. The necropolis covered an area of 25 ares and was fully recognized. In more than half of the graves, the presence of equipment pieces was noted, but

no traces of any structures were observed. The only exception was a single grave near which two post-holes were captured. Jewellery, tools, weapons, and two coins were discovered in the pits. The cemetery was characterized by a row system of skeletons' arrangement with the dominant orientation along the east-west axis. The graves did not intersect with each other. Children's burials were dug into the cavities of adults. A single, multi-storey grave has also been observed. The necropolis dates back to the last decades of the 10th to the beginning of the 13th century.

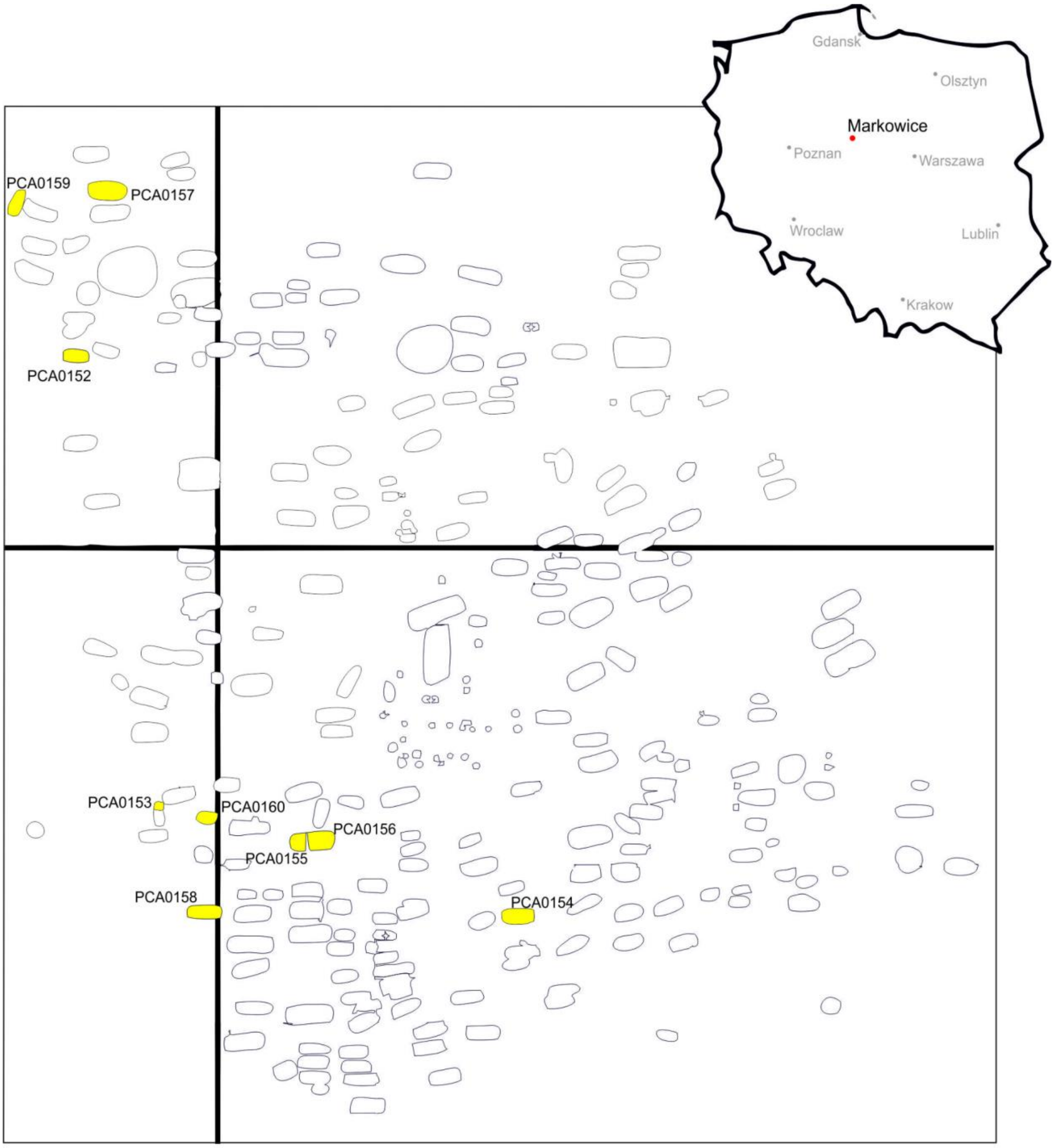

Fig. S9: Location and plan of the Markowice archaeological site. [based on Figure 1 from Błaszczyk K, Nierychlewska A, Zawilski P; modified using Corel Draw X6]

The settlement of the Lusatian culture population in Markowice (site 9) was examined in 2015, registering 18 objects containing human bones, including 2 considered to be undoubtedly graves. In both cases, the dead were placed along the north-south axis, in an upright position, on backs, with their heads directed south. One of the skeletons had the right arm bent at the

elbow with the palm folded over the stomach and the grave was deprived of furnishing. In the second one, two pottery vessels were found. The features of the burials were considered characteristic of the Częstochowa-Gliwice subgroup of the Upper Silesian-Lesser Poland Lusatian culture. The site located south-east of Markowice was dated to the 5th period of the Bronze Age and Hallstatt D (approx. 800-450 BCE).

[There is no cemetery plan available for Markowice site 9]

Milicz

An early medieval skeleton cemetery in Milicz (Lower Silesia voivodeship) was discovered on the south-western slope of a low hill located in the Barycz River valley, on its right bank. In its immediate vicinity, there is a stronghold dated from the 10th/11th century to the middle of the 13th century. At least from the mid-nineteenth century, objects indicating the existence of a necropolis here had been found on the hill, but the first excavations were carried out only in 1953, and then in 1960-1962, finally revealing about 800 burials. The estimated number of all dead buried in the cemetery could reach 1000 individuals. Most of the deceased rested upright on their backs, with arms stretched along the body and skulls facing west. The head-east-oriented skeletal system has only been reported twice. Single graves with an abnormal skeleton layout were also recorded, including burial with the skull folded under the slightly tucked legs of the deceased resting on the side of the body. In five graves, traces of stone structures were found, but only one of them can be described as the border. About 28% of the burials contained accessories in the form of ornaments (temple rings, rings, glass beads) or utility items (knives, sometimes in sheaths, horn cutters, a spindle, bone and metal needles, a whetstone with a chain, an iron chisel, and a stone grinder). There were silver coins registered in two graves. The chronology of the cemetery was established from the 2nd half of the 12th century to the 1st half 14th century.

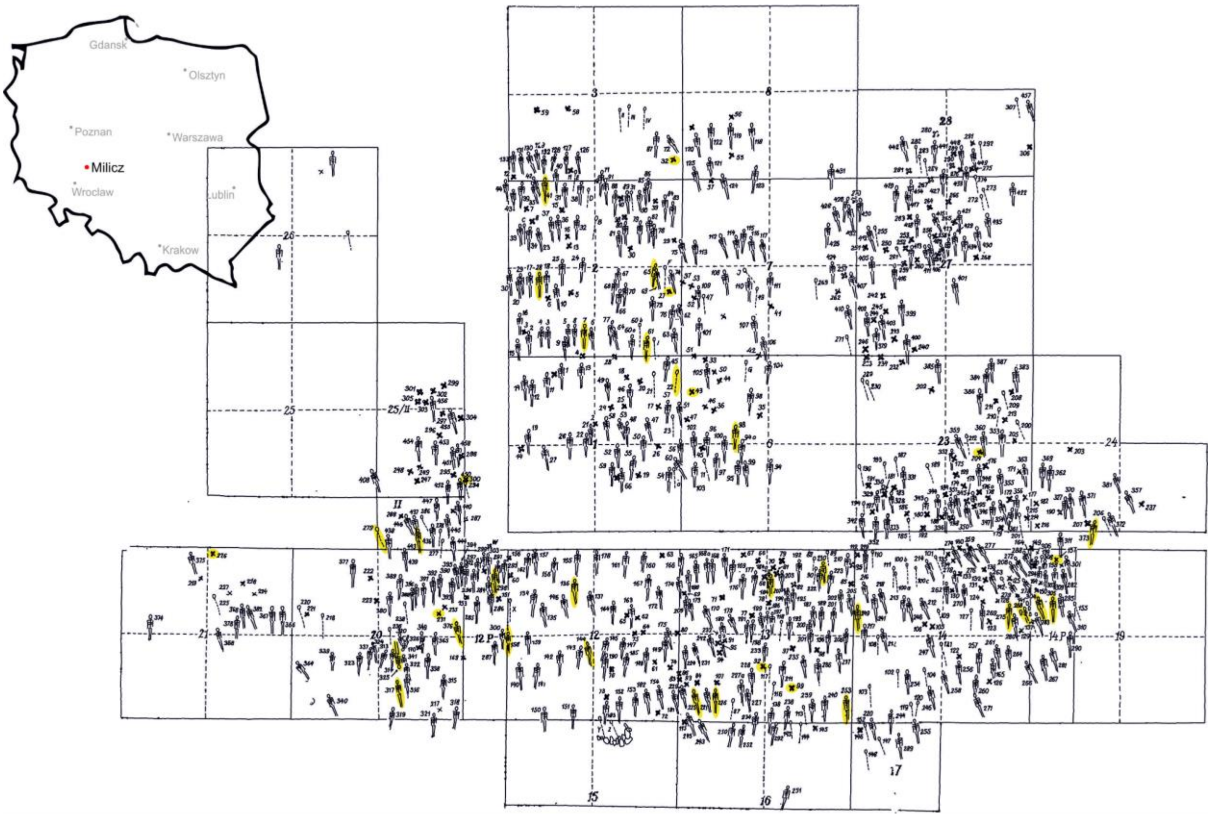

Fig. S10: Location and plan of the Milicz archaeological site. [based on Figure 2 from Wachowski K; modified using Corel Draw X6]

Niemcza

Two early medieval skeleton cemeteries were discovered in Niemcza (Lower Silesia voivodeship). The first necropolis was found in the years 1894-1895 during works on the construction of the railway line between the city and the Ślęża River, approx. 50 m from its bed (Niemcza I). At that time, several graves were discovered lying at a depth of about 1.5 m. In the graves discovered 4 ceramic vessels and, perhaps, an unspecified number of other objects today mixed with materials from a nearby settlement. The cemetery was dated between 9th and early 11th centuries.
The second necropolis was discovered on a hill in the southwestern part of the city (Niemcza II). Excavation work was carried out in 1917 and then in the years 1963-1965. In total, 90 skeletal burials and –probably one cremation burial were found here. The dead were resting at a depth of 0.7 m to 1.4 m, on the backs with their heads facing west, less often east, exceptionally south or north. The arms were usually extended along the torso, sometimes folded over the pelvis. In individual cases, traces of coffins or stone surroundings were observed. There have been several double burials of adults and women with children. The burial goods were discovered in 22% of the graves and they were ornaments (glass and semi-precious stone beads, temple rings, other copper and silver ornaments), and utility items (knives, ceramic dishes, quern). The chronology of the cemetery was established in the 10th-12th century.

[There is no cemetery plans available]

Obłaczkowo

During the archaeological work preceding the investment on site 9 in Obłaczkowo (Greater Poland Voivodeship), a previously unknown, early medieval necropolis was discovered. Excavation research was carried out in 2014, recognizing a total of 5.5 hectares, including a small cemetery with an area of less than are. In total, 24 true and 5 alleged graves were registered at the site, and no destruction of the graves of the older ones before the younger ones was found. All recorded bone remains belonged to adults. The burials were oriented along the east-west axis, with no clear preference for the orientation of the skull. The equipment of the graves was relatively modest, only temple rings and knives, sometimes shorn sheaths were recorded. Also, an iron pin, a tinder, and four wooden buckets with fittings were noted. There were probably also ceramic vessels in the graves. The necropolis was heavily damaged, and during the works, monuments and loose bones lying on the secondary deposit were observed. Only in one case traces of a wooden structure were found, while traces of burning or charcoal associated with funeral ceremonies were more often identified. The original size of the cemetery was estimated at 17-18 ares. The necropolis is dated from the end of the 10th to the end of the 11th century.

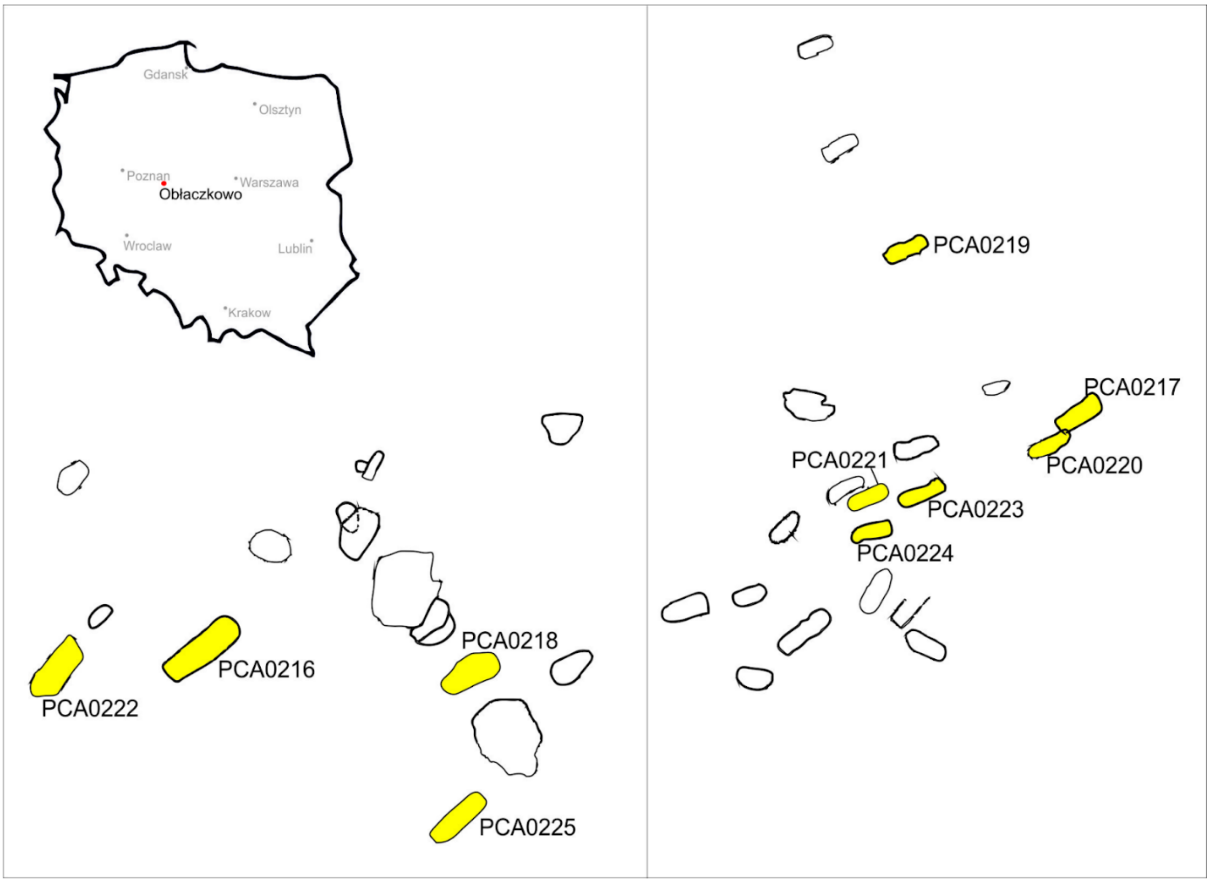

Fig. S11: Location and plan of the Obłaczkowo archaeological site. [based on the Figure 25 from Pawlak P; modified using Corel Draw X6]

Ostrów Lednicki

Research on the skeletal cemetery located within the early medieval stronghold in Ostrów Lednicki (Greater Poland voivodeship) began in 1932 and was continued for the next three years, during which the area of over 25a was examined, registering the skeletons of about 1,500 individuals. It was then that the eastern and northern surroundings of the palace chapel included in the Lednica palas complex were recognized. Numerous intersecting burials laid in 2-3 layers at a depth of 0.35 to 1 m. Unfortunately, some documentation and materials from that period were lost during the Second World War. Research resumed in 1948 is still carried out today. Taking into account the results of the interwar works, so far, approximately 2,000 burials dating from the end of 11th to the beginning of the 14th century have been examined in the castle area. Within the stronghold, two burial zones can be distinguished: i) a row cemetery around the palace chapel and ii) a necropolis dated from the second half of the 11th century next to and inside the so-called *second church*, including two burials in stone tombs. Most often, there is no information about the furnishing of the graves, some of the burials did not contain it, but in some cases the equipment was lost. In a few cases, decorations have been preserved, including a gold and a silver wedding ring.

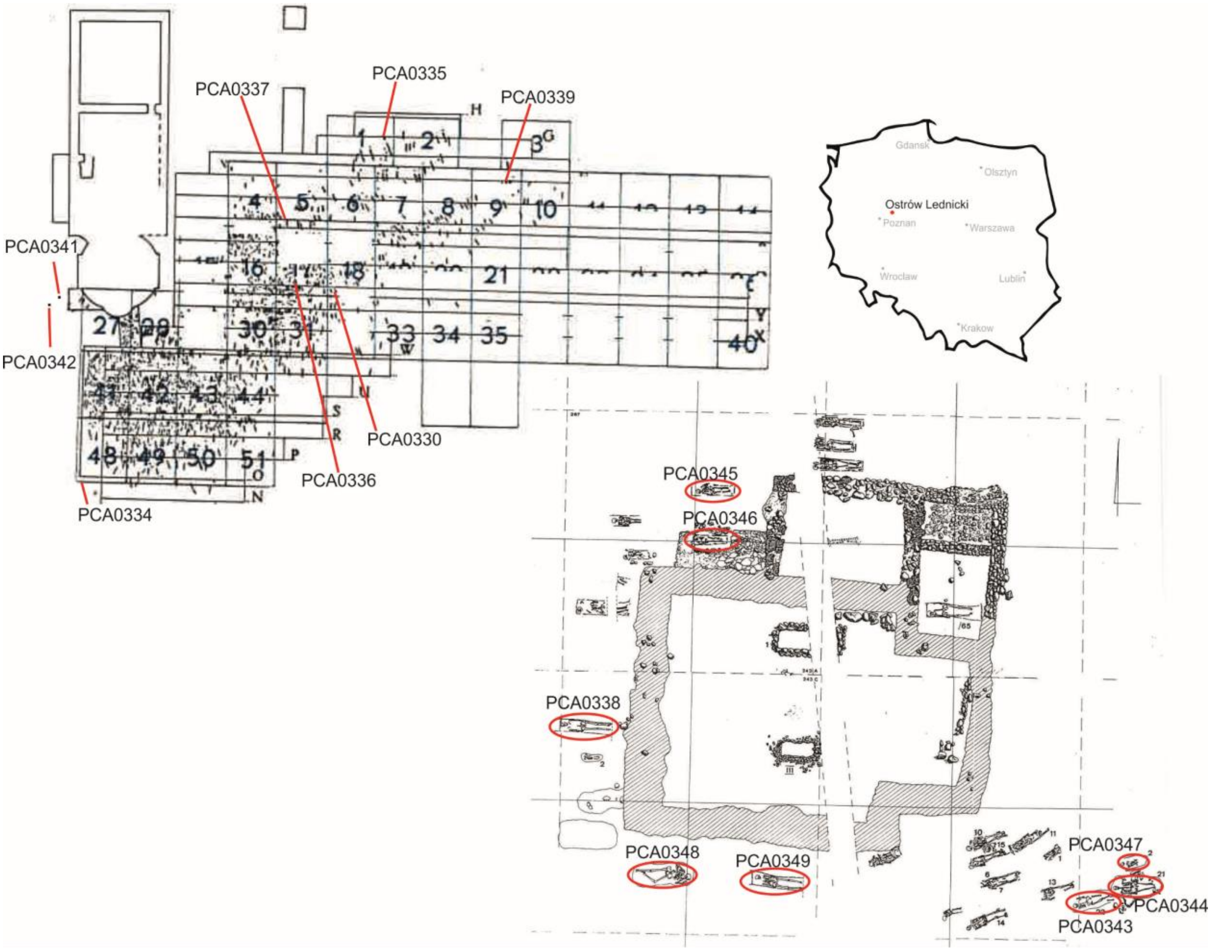

Fig. S12: Location and plan of the Ostrów Lednicki archaeological site. [based on the Sheet 1 from Ćwirko-Godycki and Figure IX.3. from Wrzesiński J, The history of research and the graves location [Historia badań i lokalizacja grobów] (in:) Ostrów Lednicki. Residential and capital center of the first Piasts [Ostrów Lednicki. Rezydencjonalno-stołeczny ośrodek pierwszych Piastów] Kurnatowska Z, Wyrwa AM, Bis M (ed.), Warszawa 2016

Płońsk

Early medieval cemetery in Płońsk (site 5, Mazovian voivodeship) is located on the floodplain terrace of the Płonka River, on its right bank. Due to the location of the catholic cemetery in this place, the necropolis had been systematically destroyed for many years as a result of subsequent funerals. In the 1970s, archaeological rescue research was carried out here in connection with the construction of apartment blocks. In 1992, archaeological supervision was carried out in connection with the construction of the heat pipeline. At that time, about 200 m^2 of the cemetery area was discovered, and several dozen destroyed graves were found. Due to the destruction, the furnishings of individual graves do not constitute assemblies, but only loose material from the necropolis. In the burials, items of armament (a spear, an ax), ornaments (temple rings, rings, and glass beads), and utility items (knives, whetstones, flints, buckets, bronze bowls) were noted. Moreover, 96 human skulls were secured from the cemetery. The necropolis is dated from the 11th to the 13th century.

[There is no cemetery plan available]

Poznań – Śródka

The latest research indicates that in the early Middle Ages, there were two different skeletal cemeteries in Poznań Śródka (Greater Poland voivodeship). The first, discovered in 1994, was examined off and on until 2019, exploring an area of approx. 700 m2. The necropolis is located on the eastern valley slope of the Cybina River, the right tributary of the Warta, in the immediate vicinity of the hillfort on the island on Warta. During the research, total of about 500 graves were recorded, dated from the end of the 10th century to the end of the 12th or the beginning of the 13th century. Three to five layers of burials were discovered in the cemetery, in which the inhabitants of the nearby stronghold were buried. In many graves, goods in the form of tools (knives, whetstones, needles and piercers), less often decorations and coins, were recorded. As much as 60% of the graves were characterized by the presence of wooden structures, from simple surrounds to coffins, and in a few cases stone surrounds were also found. The second cemetery in Śródka was discovered in 2018, approx. 100 m east of the previous one. At that time, 15 skeletal graves, also lying in several layers, were explored. Also in this necropolis, traces of wooden structures were observed in the pits. This cemetery is dated from the 10th to the12th centuries.

[There is no cemetery plan available]

Poznań – Sołacz

The early medieval cemetery in the Sołacz district in Poznań (site 14, Greater Poland voivodeship) was discovered in the 1930s, during construction works carried out on the property located at Góralska Street. At that time, five skeletal burials and anthropological material from six other graves destroyed by workers were registered. Later construction actions provided information about bone remains found on neighboring properties. The total number of tombs discovered in this area could reach approx. 30. Relatively recently, the cemetery has again attracted the attention of medievalists. Among others, there is the thesis that the cemetery was associated with the founders of the town in Ostrów Tumski, who were perceived as strangers from the circle of Great Moravian culture. The last excavation work was carried out here in 2016, but only a single tibia lying on the secondary layer was recorded then and its chronology was determined by the ^{14}C method for the 11th-12th centuries. The furnishings of the tombs were rather modest and limited mainly to ornaments, including a silver bead - a gombik with a clear southern influence. Originally, the cemetery was dated to the 10th and 11th centuries, but the discovery from 2016 also indicates a slightly younger chronology of the site.

[There is no cemetery plan available]

Rumin

Skeleton cemetery in Rumin (site 10; Greater Poland voivodeship) was discovered during surface surveys in 1953. Its existence was confirmed in 1959, but only partial destruction of the site during the earthworks carried out in 1989 made it necessary to carry out rescue excavations. In addition to the eight destroyed graves, eight more burials preserved in a slightly better condition were uncovered, indicating the row layout of the necropolises. The dead were buried along the east-west axis, but without consistent orientation, number of skeletons facing east and west were even. The burials were relatively poorly equipped, almost only knives were noted, and only one female grave was found to have a temple ring. The cemetery was located on a small promontory above the edge of the Warta valley. The discoverers adopted a fairly wide

dating of the cemetery for the period between the mid-11th century and the turn of the 13th and 14th centuries.

[There is no cemetery plan available]

Santok

The remains of the vast necropolis in Santok (Lubuskie voivodeship) were discovered in 2014 during archaeological research accompanying the construction of sanitary sewage system. The cemetery is located on the Warta River terrace, at the foot of one of the two Santok strongholds referred to as *Pomeranian*, in contrast to the fortified settlement on the other bank of the river, which was part of the Piast State. The excavations were carried out in a 1.4-2 m wide strip, recording skeletal graves in a section of about 430 m. During the rescue excavation, 197 burials were registered, lying on several levels, but the final anthropological analysis showed that as many as 328 individual remains were excavated from the graves. The cemetery dates back to the turn of the 11th and 12th centuries, up to the 13th century. Tools (whetstones, knives), ornaments (rings, temple rings) and single coins were discovered in the burials. In numerous cases, traces of wooden structures, including coffins, were also recorded.

The research results have not yet been published.
[There is no cemetery plan available]

Słaboszewo

Cemetery in Słaboszewo (Kuyavian-Pomeranian voivodeship) is situated on a small hill, next to the road leading to the neighboring village Krzekotowo. It was discovered for the first time in 1877 when a box-like grave of Pomeranian culture was registered. In the years 1878-1879, excavations were carried out there, revealing 60 early medieval skeletal burials. Due to the destruction of the site, archaeological works were carried out in 1969 and 1972-1974, registering a further 454 graves, 53 bone clusters, and a significant amount of loose bones. Also, the stone foundations of the church around which the dead were buried were discovered. Skeletons laid in layers, in some places 6 levels of graves were observed. The furnishing of the burials included coins (usually found in the mouth) rings, pins, buttons, and even pieces of clothing. Some of the skeletons were in the coffins. The chronology of the cemetery was based mainly on written sources and coins. The oldest graves date back to the 14th century, while the youngest ones come from the 17th century. However, materials from older studies indicate that some of the burials date back to the early Middle Ages (12th century).

[There is no cemetery plan available]

Sowinki

A vast necropolis in Sowinki (site 23A; Greater Poland voivodeship) was discovered as part of the research in 1982. The site is situated on a large, sandy promontory over the edge of the terrace over the Warta river valley. Excavation works covering an area of almost 1.5 hectares were carried out here in 1989-1991, in connection with the implementation of the construction investment. In addition to the early medieval skeleton cemetery, traces of settlement from the Stone Age, Bronze Age, Roman period, and the Middle Ages were also recorded. In the end, the necropolis was examined in its entirety, revealing 150 graves containing 158 skeletons. No anthropological material was found in eight pits. Most of the burials were oriented along the

east-west axis, only in a few cases the north-south orientation was observed. The dead were equipped with jewelry, coins, armaments (spear, ice ax), and numerous tools, including the merchant's utensils. Due to the form (construction) and furnishing, several graves were considered chamber-type, hiding the remains of representatives of the local elite. The cemetery chronology was set at the turn of the 10th/11th century and the first half of the 12th century, separating two phases of the use: i) the turn of the 10th/11th century to the 1st half of the 11th century, and ii) the second half of the 11th century to the first half of the 12th century.

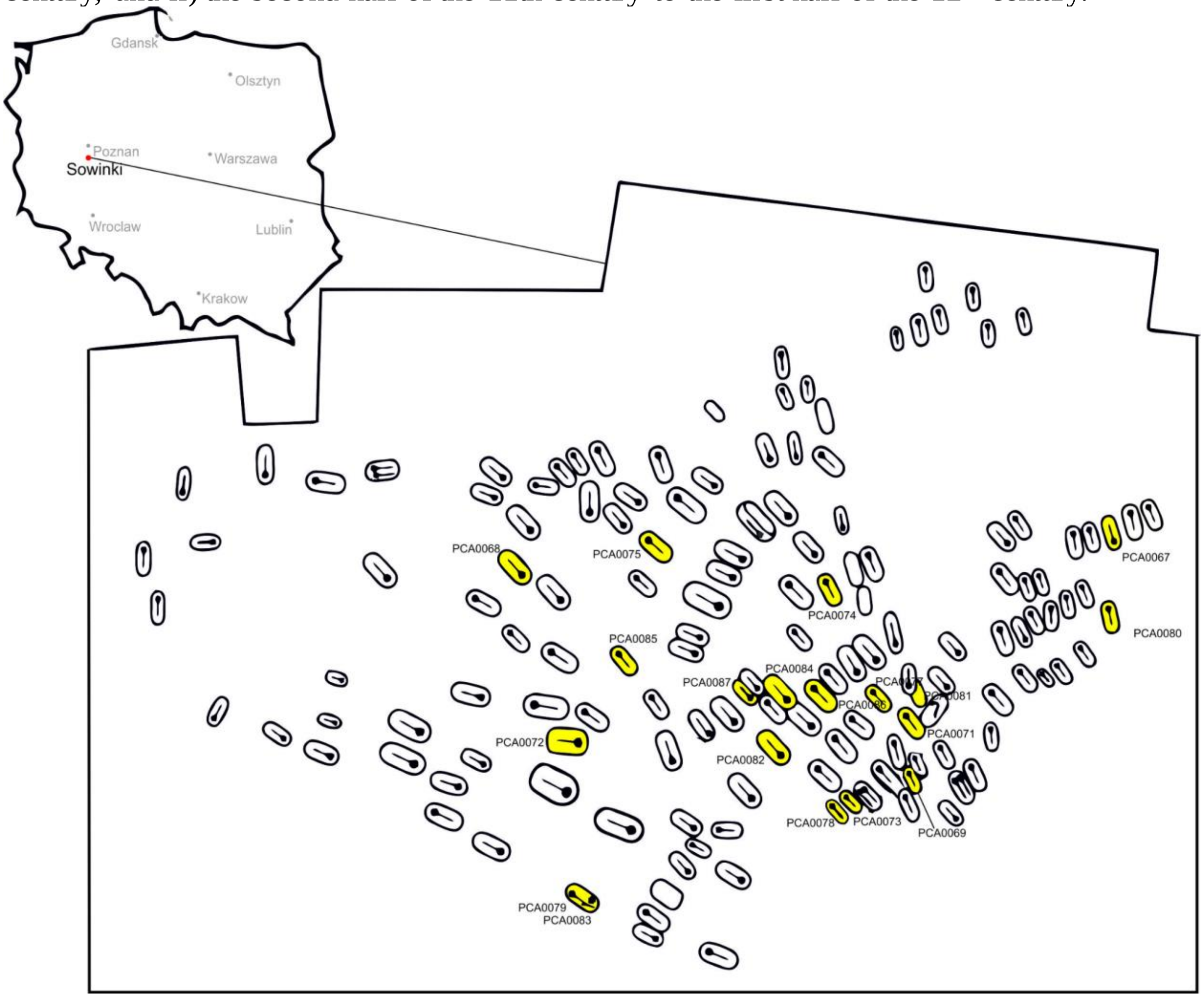

Fig. S13: Location and plan of the Sowinki archaeological site. [based on Figure 3 from Krzyszowski A; modified using Corel Draw X6]

Zielonka

Zielonka is a small village located in a large forest complex known as Puszcza Zielonka (Greater Poland voivodeship). The early medieval barrow burial located 2.5 km from the village was discovered in 1976 during forestry works (site 3, initially known as Dąbrówka Kościelna, site 1). Archaeological research on the site was carried out in 1977, 1992-1993, and 2017. As a result, there were discovered barrows partially based on hills of strongly undulating terrain. Within the embankments, four-sided stone structures were discovered with skeletal burials also covered with stone mantles. In total, the remains of nine individuals were excavated, and two objects considered symbolic graves were registered. The equipment was registered in two burials, one with a temple mount and a knife, and the other with a wooden bucket, knife, arrowhead, and a pair of spurs. The graves, based on archaeological material and radiocarbon samples, were dated at the end of the 10th to the 1st half of the 11th century. The cemetery with

a unique grave structure in Greater Poland is considered to be a material trace of the stay of newcomers, probably from the area of south-eastern Pomerania.

[There is no cemetery plan available]

SUPPLEMENTARY NOTE 3. RADIOCARBON DATING.

Radiocarbon dating of the bone samples was performed at the Poznan Radiocarbon Laboratory (Foundation of the A. Mickiewicz University), which specializes in this type of analysis, according to the protocol published by Goslar et al. 2004. The laboratory performs ^{14}C dating using the AMS technique and is equipped with the 1.5 SDH-Pelletron Model “Compact Carbon AMS” ser. no. 003.

The analysis procedure consisted of several steps:

1) Chemical pretreatment

Collagen from bone material was extracted according to the protocol by Longin with modifications. All samples underwent collagen degradation analysis on Flash EA 1112 analyser. Samples showing nitrogen content >0.6% and a C/N ratio <5 were considered suitable for collagen dating. were chosen based . Suitable bones were crushed and bone powder was treated with 2M HCl and 0,1M NaOH with spinning and collecting sediment after each treatment. Collagen extraction was carried out in HCl, and after centrifugation, the collagen-containing supernatant was ultrafiltered and purified on Vivaspin 15 MWCO 30kD filters. Collagen quality was defined based on the atomic C/N ratio with accepted values between 2.7 and 3.5.

2) Production of CO_2 and graphitisation

CO_2 was produced by combusting samples performed at 900°C in sealed quartz tubes together with CuO and Ag wool. The obtained gas was dried in a vacuum lime and reduced with H_2 using Fe powder as a catalyst. The obtained C and Fe mixture was then pressed into a special aluminium holder.

3) AMS ^{14}C measurement

Content of ^{14}C was measured as described by Goslar. Briefly it was performed by comparing intensities of ^{14}C, ^{13}C, and ^{12}C ionic beams measured for each sample and for standard sample which was Oxalic Acid II- international modern 14C standard.

4) Calculation of ^{14}C age and calibration of ^{14}C age

^{14}C age was calculated with correction for isotope fractionation on the basis of the $^{13}C/^{12}C$ ratio measured in the AMS spectrometer simultaneously with the $^{14}C/^{12}C$ ratio with 1-sigma uncertainty. ^{14}C age calibration was performed with OxCal v.4.2 program.

[description based on the Laboratory’s materials by Tomasz Goslar (2021) (https://radiocarbon.pl/wp-content/uploads/2018/07/procedure_ams_plr.doc)]

SUPPLEMENTARY NOTE 4. DNA ISOLATION, LIBRARY PREPARATION, SCREENING AND IN SOLUTION ENRICHMENT

DNA isolation

Bone samples (mainly teeth, petrous bones in some cases) were obtained for 474 individuals from 27 archaeological sites listed in Supplementary Table S1 and described in supplementary note 1. After being transported to a specialized ancient DNA laboratory (Institute of Human Biology and Evolution, Adam Mickiewicz University Poznan), the bone material was cleaned with 5% NaOCl and rinsed with sterile water, followed by UV irradiation (254 nm) for 2 hours per side/plane. The teeth and bones were drilled using Dremel® drill bits. Bone powder (approximately 250 mg) was digested with proteinase K and DNA-containing extract was purified using a silica-based method following the procedure described by Yang and Svensson.

Library preparation

Genomic libraries were prepared following the protocol described by Meyer and Kircher 2010, omitting the initial sonication step due to the natural fragmentation of aDNA. Six separate PCR reactions were set up for each library. PCR amplifications were performed in 25 µl with 3 µl of the DNA library template, 12.5 µl of 1x AmpliTaq Gold® 360 Master Mix (Life Technologies, California), 0.5 µl of indexing primer (10 µM) and 0.5 µl of PCR primer IS4 (10 µM) (Günther *et al*. 2015). The PCR profile was as follows: initial denaturation (94 °C, 12 min), 12–16 cycles of 94 °C (30 s), 60 °C (30 s), 72 °C (45 s) and final extension (72 °C, 10 min). PCR reactions for the same library were pooled and purified with AMPure® XP beads (Agencourt-Beckman Coulter) following. Quality and size distribution of the libraries were verified with High Sensitivity DNA or DNA 1000 kits, and 2100 Bioanalyzer system (Agilent). DNA concentration was determined with a Qubit fluorimeter and Qubit dsDNA HS Assay Kit (ThermoFisher Scientific), according to the manufacturers' protocols.

Library screening

To quantify human DNA content, all of the obtained barcoded libraries were first subjected to shallow (low depth) sequencing with the use of Illumina GAIIx, NextSeq 550 or HiSeq 2500/4000, and single-end (75-100 bp) or paired-end (2 x 100 bp) sequencing runs. On average, 1-5 mln reads per library was obtained. Raw sequencing data (fastq files) were processed as described previously and here in Supplementary Note 5. Where possible, genetic sex was estimated using the Ry method, by estimating the proportions of NGS reads aligned to sex chromosomes.

Deep sequencing

Based on the screening results, libraries were subjected directly to deep sequencing (when human DNA content was > 15%) or further processed before deep sequencing (enriched with human DNA). Finally, deep sequencing was performed with the use of Illumina HiSeq 4000 or HiSeq X Ten. The sequencing depth depended on the library quality and enrichment efficiency. Raw data were analyzed as described previously and below in Supplementary Note 5.

Human DNA enrichment

As a general rule, libraries with human DNA content <1% were subjected to mitochondrial DNA enrichment (MtE; in-solution hybridization capture with the whole mitochondrial genome), whereas libraries with human DNA content 1-15% were subjected to whole genome enrichment (WGE; in-solution hybridization capture with the whole human genome). Libraries with assigned male genetic sex, were separately subjected to Y-chromosome enrichment (YCE; in-solution hybridization capture with selected Y-chromosome regions covering target SNPs). When a limited amount of starting libray was available, MtE and YCE were performed together in a single reaction.

All DNA enrichment types were performed using myBaits target capture kits from MYcroarray company (now Daicel Arbor Biosciences, Michigan, USA) and an in-solution hybridization capture procedure recommended by the manufacturer (v. 3.0).

Whole human genome enrichment
WGE was performed mostly with myBaits Human WGE kit, containing reagents and hybridization capture probes targeting more than 2 million sites in the human genome known to be polymorphic in living and ancient world populations. The kit was designed and tested in collaboration with leaders in ancient human population genetics. One round of enrichment was applied, followed by 14 amplification cycles.

mtDNA enrichment
MtE was performed with myBaits Mito-Human rCRS kit (Daicel Arbor Biosciences, Michigan, USA), containing reagents and a set of 80-bp long tiling probes designed based on rCRS (revised Cambridge Reference Sequence). Two rounds of enrichment were applied, the first followed by 14, and the second by 10 amplification cycles.

Y-chromosome enrichment
Enrichment in selected Y chromosome regions was performed with myBaits Human SNPs kit (Daicel Arbor Biosciences, Michigan, USA), containing reagents and custom probes (listed in Supplemetary Table S5) dedicated to the selected by us regions of Y chromosome and some additional SNPs located on other human chromosomes. Two rounds of enrichment were applied, the first followed by 14, and the second by 10 or 11 amplification cycles.

SUPPLEMENTARY NOTE 5. Ancient DNA analysis, autosomal SNP and Genotype Calling

Data processing

Fastq data were trimmed by 3 nucleotides from 3’ and 5’ ends and aligned using BWA ver. 0.7.10 to the GRCh37 reference genome. Seeding was blocked using -l 1000 option. For paired-end data, overlapping reads were merged with AdapterRemoval. After the alignment, base quality scores were recalibrated with mapDamage 2.0. Data were deduplicated with picard-tools on the library level and reads with average quality >= 20 were selected. Libraries from the same individuals were merged. Relative coverage on the X- and Y-chromosome was used to confirm genetic sex assignment. Nuclear contamination was estimated in males with the ANGSD by calculating the heterozygosity levels on known X-chromosome polymorphic sites. Pseudo-haploid genotypes were called at SNP positions from the 1240K reference set by randomly selecting 1 read for all ancient individuals sequenced as part of this study and modern reference samples. Genotype calls were converted to the PLINK file format.

Contamination estimates and verification of authenticity

We verified authenticity of ancient DNA by determining of deamination rates as described below. For estimating contamination in the ancient samples sequenced we followed two approaches : 1) mtDNA-based and 2) X-chromosome-based in males, also described below.

Estimation of deamination rates
Untrimmed reads were analysed with mapDamage2.0 with the purpose of investigating deamination patterns. We examined: (i) λ, the fraction of nucleotides positioned in single-

stranded DNA overhangs context, (ii) δs, C → T deamination probability in the single-stranded overhangs context, and (iii) δd, C → T deamination probability in the double-stranded DNA context.

Mitochondrial DNA contamination estimates

To estimate the level of present-day human contamination in the ancient samples we used the software schmutzi. This software provides a database of Euroasian allele frequencies. By comparing each sample with the database, the software estimates the contamination rates and identifies the most likely contaminant in the database of mtDNA sequences.

X-chromosome contamination in males

We used ANGSD to estimate X-chromosome contamination based on a previously published method. In male individuals, which are haploid for the X-chromosome, mismatches at polymorphic sites can be interpreted as either contamination or sequencing errors. In order to account for the latter, polymorphic sites and also their adjacent bases are screened for mismatches, because sequencing errors should be observed at identical frequencies across the genome. The analysis was done by using default parameters and the provided mapFile ("RES/chrX.unique.gz") and hapFile ("RES/HapMapChrX.gz"). We only considered bases with base quality ≥20 and mapping quality ≥30. These restricted the analysis to unique regions of the X-chromosome and to HapMap X-chromosome polymorphisms. Two methods were applied. Briefly, in Method 1, the major and minor base are detected for each SNP position and adjacent sites, using the latter to assess background error rates for each sample. While Method 1 assumes that errors between reads and sites are independent, Method 2 randomly samples a single read at each site prior to determining the number of major and minor bases.

Genetic sex estimation

Genetic sex of each individual was estimated using the Ry method as described by Skoglund et al. The method is based on dividing the number of sequences mapped to the Y chromosome to the number of those mapped to X and Y chromosomes. Only sequences with mapping quality of minimum 30 were considered. Sex assignment was performed for samples with at least 3000 reads aligned to the sex chromosomes.

Kinship analysis

Relationship estimation from ancient DNA (READ) software was used to investigate kinship between the individuals. READ uses pseudo-haploid genotype data as input and kinship up to 2nd degree based on the proportion of nonmatching genotypes (P0) estimates. Samples from each location were analyzed separately and default parameters were applied. Only first degree relatives (i.e. sibling, parent/offspring) were considered. From each pair of related individuals, one was randomly chosen and discarded from further analysis.

Phenotypic inference

We determined the alleles carried by the ancient individuals in 41 HIrisPlex-S System SNPs. HIrisPlex-S allows the inference of pigmentation (eyes, skin and hair) phenotype in humans, but relies on diploid genotypes for each studied individual. Given this, we were limited to infer the phenotype of each ancient individual. To circumvent this problem we followed the approach described by Lazaridis et al and calculated the population wise allele frequencies for each

HIrisPlex-S System SNPs. Briefly, we generated 100 replicates from each ancient individual, assigning their genotypes randomly according to the distribution. We recorded the mean and standard deviation of the allele frequency of the phenotypic SNPs in both IA and MA groups.

SUPPLEMENTARY NOTE 6. MODERN AND ANCIENT REFERENCE DATASET CONSTRUCTION

For comparative analysis of ancient individuals sequenced in this study, we assembled SNP reference datasets for 4 modern populations and one ancient population reference dataset. Datasets were merged using PLINK and custom Python scripts. Diploid genotypes for modern and ancient samples were transformed into pseudo-haploid calls by picking one allele at random.

The following datasets were used:

Human Origins dataset:
The Affymetrix Human Origins dataset consisting of 6472 present-day individuals genotyped at 597,573 loci. We selected individuals from Western Eurasia and pruned the data for MAF >=5% and linkage disequilibrium >= 0.6.

POPRES dataset:
The European Population Reference Sample project (POPRES) dataset, consisting of 3,192 individuals genotyped at 505,586 loci, published by Novembre et al. From this dataset we selected only individuals from Europe. Given that some populations were unequally represented in the dataset, we subsampled each population up to 100 random individuals to balance the PCA. Further, we pruned the data for MAF >= 5% and linkage disequilibrium >= 0.6.

EGDP dataset:
Estonian Biocentre Human Genome Diversity dataset consisting of 402 individuals sequenced to a high coverage. We selected only individuals from Europe. The SNPs were filtered to be at >= 5% MAF and >= 0.6 linkage disequilibrium.

Ethno-linguistic dataset:
We constructed the ethno-linguistic dataset from panels representing the genetic structure of present-day Germanic, Slavic and Norse people from Europe. To this end, for each panel we imputed additional SNPs using the Michigan Imputation Server. Phasing was performed using the Eagle2 with the worldwide HRC panel consisting of 64,976 haplotypes at 39,235,157 SNPs as the reference panel. Only SNPs overlapping with the 1240K capture with r2 imputation >= 0.9 were retained for each dataset. After merging data from all modern-individual datasets, we filtered the SNPs to be at >= 5% MAF and >= 0.6 linkage disequilibrium.

Allen Ancient DNA Resource (AADR) V42.4:
Genotypes of available ancient individuals published by March 2020. The dataset consisted of curated genotypes of 3589 ancient individuals at ~1.2 mln positions.

SUPPLEMENTARY NOTE 7. POPULATION GENOMIC ANALYSES

Principal Component Analysis

We performed all PCA analyses using smartpca on pseudo-haploid calls of modern reference datasets and projected on them the pseudo-haploid genotypes of ancient individuals.

First, we performed PCAs using each modern reference dataset against ancient individuals from IA and MA groups. The PCA consisting Human Origins dataset placed our samples within the modern European populations. IA individuals showed a shift towards North-Western European populations, and MA individuals were located more closely to the Eastern European populations.

Since all of the studied samples appeared to overlap with modern European populations, to gain better resolution, next we projected our ancient individuals on the PCA of POPRES reference dataset. Since, the dataset is highly unbalanced with regard to how many individuals from each population were studied, we randomly selected from the POPRES dataset up to 100 individuals from each European population. This step ensured that the PCA is not biased towards North-Western European populations. We observed clear separation of the IA and MA individuals. As in PCA with the Human Origins dataset, IA individuals overlapped with the North-Western European populations, and MA individuals with the Eastern European populations.

To study in greater detail the affinities of ancient individuals from the IA and MA groups with respect to the modern populations most similar to them, we have performed a PCA on the constructed ethno-linguistic dataset. Here, to avoid the problem of PCA shrinkage we have calculated the PCs from the modern individuals and projected on them both the ancient individuals as well as modern individuals, for which we randomly sampled ~50% of the SNP positions.

Estimation of Fst-coefficients

Pairwise F-st were calculated between modern European populations and IA and MA groups using PLINK ver. 1.9, utilizing Weir and Cockerham’s formula.

SUPPLEMENTARY NOTE 8. MODEL-BASED CLUSTERING ANALYSIS ADMIXTURE

We performed the model-based clustering analyses using ADMIXTURE software. Firstly, we wanted to understand how IA and MA individuals studied here were related to contemporary populations. For this we merged individually each of the ancient individuals with the Human Origins dataset and run an unsupervised ADMIXTURE analysis. We chose K=9, as it matches the number of distinct population groups from PCA. As the result we obtained clustering with genetic components identified by us as: African (green), Caucasian (yellow), Basque (dark orange), Sardinian (red), Near Eastern (blue), Bedouin (teal), North-Western European (dark blue), Druze (navy blue), and Eastern European (light orange).

Following our observations from the PCA and ADMIXTURE, where IA and MA individuals expressed high similarities with present day North-Western and Easter-European populations we performed a supervised admixture analysis with K=2 for each IA and MA individual with respect to genetic components representing North-Western Europeans and Central-Eastern Europeans.

Previous works have shown that European genetic variation stems from the admixture of the three ancestral groups: Paleolithic Hunter-Gatherers, Neolithic farmers from Anatolia and Bronze Age Steppe Herders. To show that the separation of IA and MA individuals on the PCA plots, and differences in the ADMIXTURE results are not the result of pre-Iron Age migration we have performed a supervised ADMIXTURE for each IA and MA individual with respect to the ancient populations representing 4 major genetic components in present-day Europe.

Ancient populations f4 statistics

To better understand genetic affinities of studied IA and MA populations with earlier and contemporary ancient populations we calculated a comprehensive set of f4 statistics of the form f4(Test population, MA, Ancient population, Yoruba) and f4(Test population, IA, Ancient population, Yoruba), where Test population is (i) one of the studied IA populations and (ii) one of the MA populations, and Ancient population is one of the following 459 populations:

Albania_BA_IA, Albania_MBA, Albania_Me, Armenia_C, Armenia_EaMe, Armenia_EBA_KuraAraxes, Armenia_EIA, Armenia_IA, Armenia_LBA, Armenia_LBA_EIA, Armenia_Lchas_LBA, Armenia_LIA, Armenia_MBA, Bulgaria_C, Bulgaria_EBA, Bulgaria_EIA, Bulgaria_Me, Croatia_BA, Croatia_C_Lasinja, Croatia_EIA, Croatia_Jagodnjak_MBAs, Croatia_LBA_EIA, Croatia_MBA, Croatia_MBA_Cetina, Croatia_Me, Croatia_Metz_GalloRomans, Croatia_MirineFulfinum_Romans, Croatia_MLBA, Croatia_Mursa_Romans, Croatia_Sipar_Romans, Croatia_SisakPogorelec_Romans, Croatia_Tilurium_Romans, Croatia_Zadar_Romans, Czech_BellBeaker, Czech_C, Czech_CordedWare, Czech_C_Baalberge, Czech_EaSlavs, Czech_EBA, Czech_EBA_Protounetice, Czech_EBA_Starounetice, Czech_EBA_Unetice, Czech_IA_Hallstatt, Czech_IA_Lne, Czech_LBA_IA_Knoviz_Hallstatt, Czech_LBA_Knoviz, Czech_MBA_Tumulus, Czech_Me, Czech_N_GlobularAmphora, Czech_N_Jordanow, Czech_N_Rivnac, Czech_N_TRB, Denmark_BAs, Denmark_SinGrCul, Denmark_EaVikings, Denmark_IAs, Denmark_LBAs, Denmark_LNs, Denmark_LN_BAs, Denmark_Vikings, Denmark_Zealand_Sx_Me, England_BellBeaker, England_BellBeaker_highEEF, England_BellBeaker_lowEEF, England_C_EBA, England_C_EBA_highEEF, England_EaMe, England_EaMe_Sx, England_EaMe_Sxs, England_EastYorkshire_Anglian, England_EastYorkshire_EIA, England_EastYorkshire_LIA, England_EastYorkshire_MIA, England_EastYorkshire_MIA_LIA, England_EBA, England_EIA, England_IA, England_IAs, England_IA_EaMe, England_IA_ERomans, England_IA_Romans, England_LBA, England_LIA, England_MBA, England_MIA, England_MIA_LIA, England_Norfolk_AngloSx_EMe, England_NorthYorkshire_EBAs, England_Sx, England_Sxs, England_Scorton_Anglian, England_Vikings, Estonia_BAs, Estonia_CordedWares, Estonia_EaVikings, Estonia_IAs, Estonia_Mes, France_BellBeaker, France_EBA, France_EBA_BellBeaker, France_GrandEst_EBA, France_GrandEst_EBAs, France_GrandEst_IA1s, France_GrandEst_IA2, France_GrandEst_IA2s, France_LaClape_LN_EMBAs, France_Occitanie_EBAs, France_Occitanie_EMBAs, France_Occitanie_IA2s, France_LAntiquitys, France_SouthEast_IA2, Germany_AltInden_Sx_EMe, Germany_Anderten_Sx_Me, Germany_BAs, Germany_BellBeaker, Germany_CordedWare, Germany_CordedWares, Germany_Drantum_Sx_Me, Germany_Dunum_Sx_Me, Germany_EaMes, Germany_EBA_Unetice, Germany_EMe_Alemanic, Germany_EMe_Alemanic_SEurope, Germany_Hassleben_Germanics, Germany_Germanic_elite_1s, Germany_Germanic_elite_2s, Germany_Hiddestorf_Sx_EMe, Germany_Issendorf_Sx_EMe, Germany_Lech_BellBeaker, Germany_Lech_CordedWare, Germany_Lech_EBA, Germany_Lech_MBA, Germany_Liebenau_Sx_EMe, Germany_Schleswig_Sx_LMe, Germany_Schortens_Sx_EMe, Germany_SouthGermany_EBA, Germany_Tollense_BAs, Greece_BA_Mycenaean, Greece_Crete_Armenoi, Greece_Delphi_BA_Mycenaean, Greece_Delphi_IA, Greece_Koufonisi_Cycladic_EBAs, Greece_LBA, Greece_Logkas_MBAs, Greece_Minoan_Lassithi, Greece_Minoan_Odigitria, Greenland_EaNorses, Greenland_LNorses, Greenland_Saqqaqs, Hungary_BAs,

Hungary_Conqueror_Commoner, Hungary_Conqueror_Elite, Hungary_C_Balaton_Lasinja, Hungary_DanubeTisza_EAvar, Hungary_DanubeTisza_LAvar, Hungary_LSarmation_EHun, Hungary_DanubeTisza_MAvar, Hungary_DanubeTisza_MLAvar, Hungary_EaArpadian, Hungary_EaAvar, Hungary_EaAvar_Elite, Hungary_EaC_Tiszapolgar, Hungary_EaMidAvar, Hungary_EBA_BellBeaker, Hungary_EIA, Hungary_EIA_Prescythian_Mezocsat, Hungary_Hun, Hungary_Hun_oEastAsian, Hungary_IA_Lne, Hungary_IA_Scythian, Hungary_IA_Scythians, Hungary_Szolad_MigrationPeriod, Hungary_LAvar, Hungary_LAvar_Elite, Hungary_LC_Baden, Hungary_LC_Baden_contam, Hungary_LC_EBA_Baden_Yamnaya, Hungary_LC_Protoboleraz, Hungary_Lne, Hungary_LBA, Hungary_LN_Lengyel, Hungary_LN_Sopot, Hungary_LN_Tisza, Hungary_Maros_EBAs, Hungary_MidAvar, Hungary_MidAvar_Elite, rmrm, Hungary_MiddleAvar, Hungary_MidLAvar, Hungary_MidLAvar_Elite, Hungary_NorthHunMon_LAvar, Hungary_Tiszaregion_EAvar, Hungary_Tisza_LN, Hungary_Transtisza_EAvar, Hungary_Transtisza_LAvar, Hungary_Trans_LSarmation_EHun, Iceland_Ea_Christians, Iceland_Pre_Christians, Iceland_Vikings, Iran_C_SehGabi, Iran_C_TepeHissar, Iran_Hasanlu_IA, Iran_Hasanlu_IAn, Iran_ShahrISokhta_BA1, Iran_ShahrISokhta_BA2, Ireland_EBAs, Ireland_AngloSx_EMe_Norman, Ireland_LNs, Ireland_Vikings, Israel_Ashkelon_IA1, Israel_Ashkelon_IA2, Israel_Ashkelon_LBA, Israel_C, Israel_MLBA, Italy_Basilicata_Venosa, Italy_CasalBertone_RomanImperial, Italy_IA_Republics, Italy_Imperials, Italy_IsolaSacra_RomanImperials, Italy_LAs, Italy_Lazio_Viterbo_EaMe, Italy_Lazio_Viterbo_Etruscan, Italy_Mes, Italy_Me_EaModerns, Italy_North_EaMe_Langobards_1, Italy_North_EaMe_Langobards_2, Italy_North_EaMe_Langobards_3, Italy_North_Remedello_Cs, Italy_North_Remedello_EBAs, Italy_PianSultano_BAs, Italy_Sardinia_BA_Nuragic, Italy_Sardinia_C, Italy_Sardinia_C_BAs, Italy_Sardinia_EBA, Italy_Sardinia_IA_Punic_1, Italy_Sardinia_IA_Punic_2, Italy_Sardinia_LC, Italy_Sardinia_LBA, Italy_Sardinia_MBA, Italy_Sardinia_Me, Italy_Sicily_EBA, Italy_Sicily_LBA, Italy_TarquiniaMonterozzi_IAs, Italy_Tuscany_Etruscan, Italy_Tuscany_Siena_EaMe, Italy_Tuscany_Siena_Etruscan, Jordan_EBA, Jordan_LBA, Jordan_LBA_IA, Kazakhstan_MLBA_Kairan, Latvia_BA, Lebanon_ERomans, Lebanon_Hellenistics, Lebanon_IA3s, Lebanon_MBAs, Lebanon_Mes, Lebanon_Romans, Lithuania_BA, Lithuania_LBAs, Lithuania_LN, Lithuania_Marvele_Romans, Mbuti.DG, Moldova_BA, Moldova_Glinoe_Scythians, Moldova_IA, Moldova_LBA, Moldova_MBA_Catacomb, Moldova_MBA_MultiCordonedWare, Montenegro_Doclea_Romans, Montenegro_IA, Montenegro_LBAs, Montenegro_MLBA, Netherlands_BellBeaker, Netherlands_EBA, Netherlands_EIA, Netherlands_Friesland_Sx_Me, Netherlands_Groningen_Sx_Me, Netherlands_LBA, Netherlands_LIA, Netherlands_LNB_EBA_BellBeaker, Netherlands_MBA, Netherlands_MBA_LBA, Netherlands_MIA_LIA, Norway_IAs, Norway_LN_BAs, Norway_Mes, Norway_Vikings, Poland_BellBeaker, Poland_BKGs, Poland_ChopiceVeseleCulture, Poland_CordedWares, Poland_CordedWare_1s, Poland_CordedWare_3s, Poland_CW_ProtoUnetices, Poland_EBAs, Poland_EBA_Unetices, Poland_GlobularAmphora, Poland_Koszyce_GAs, Poland_Ksiaznice_GAs, Poland_Me_1s, Poland_Me_2s, Poland_Mierzanowice_GAs, Poland_Sandomierz_GAs, Poland_Southeast_CW_MCs, Poland_TRBs, Poland_Vikings, Poland_Weklice_Wielbarks, Poland_Wilczyce_GAs, Romania_BA_1, Romania_BA_Arman, Romania_Brailita_Me, Romania_C_Bodrogkeresztur, Romania_MBA, Romania_Me, Romania_MLBA, Russia_Afanasievo, Russia_Afanasievo.DG, Russia_Afanasievos, Russia_Alans, Russia_Andronovos, Russia_AngaraRiver_BAs, Russia_BA_Okunevos, Russia_Blagovechensk_IAs, Russia_Caucasus_EBA_Yamnaya, Russia_Caucasus_Maikop,

Russia_Caucasus_Me, Russia_EaSarmatians, Russia_EaSarmatian_SouthUrals, Russia_EIA_Sargatka_IA, Russia_Ekven_IAs, Russia_IA_Ingrias, Russia_Kalmykia_EBA_Yamnayas, Russia_Khvalynsk_Eneolithic, Russia_Kurma_EBAs, Russia_LSarmatians, Russia_LenaRiver_BAs, Russia_MBA_Poltavka, Russia_MLBA_Krasnoyarsk, Russia_MLBA_Sintashta, Russia_MLBA_Sintashtas, Russia_Petrovka, Russia_Potapovka, Russia_SaltovoMayakis, Russia_Sarmatians, Russia_Shamanka_EBAs, Russia_Siberia_Irkutsk_EBA, Russia_Siberia_Lena_EBA, Russia_Srubnaya, Russia_Srubnaya_Alakuls, Russia_Steppe_Catacomb, Russia_Steppe_Eneolithic, Russia_Steppe_Maikop, Russia_Tagars, Russia_Tuva_IA_AldyBel, Russia_Tver_Fatyanovo_BAs, Russia_Uelen_IAs, Russia_UstBelaya_Angara, Russia_UstBelaya_Angara_Me, Russia_UstIda_EBAs, Russia_Vikings, Russia_Fatyanovo_BAs, Scotland_BellBeaker, Scotland_C_EBA, Scotland_EIA, Scotland_IA, Scotland_LBA, Scotland_LIA, Scotland_MBA, Scotland_MIA, Scotland_MIA_LIA, Scotland_Orkney_IAs, Scotland_Orkney_MBAs, Scotland_Vikings, Serbia_BA_Maros, Serbia_C_BA_Tiszapolgar, Serbia_EBA_Yamnaya, Serbia_IronGates_MBA, Serbia_LBA, Serbia_Me_Gepidians, Serbia_Mokrin_EBA_Maros, Serbia_Mokrin_EBA_Maross, Serbia_Sirmium_Ottomans, Serbia_SvilosKrussevlje_Romans, Serbia_Viminacium_Roman_elite_1s, Serbia_Viminacium_Roman_elite_2s, Serbia_Viminacium_Roman_elite_3s, Slovakia_Puchov_Lne_Romans, Slovakia_IA_Vekerzug, Slovakia_LIA_Lne, Slovakia_MigrationPeriods, Slovakia_Zohor_Germanic_Romans, Slovenia_EIA, Slovenia_Emona_Romans, Slovenia_LBA, Slovenia_MBA, Spain_Almoloya_Argar, Spain_Almoloya_Argar_Ea, Spain_Almoloya_Argar_L, Spain_Aritgues_LBA, Spain_BAs, Spain_Bastida_Argar, Spain_Bastida_Argar_L, Spain_C, Spain_Carolingian, Spain_EBA, Spain_IA, Spain_IA_Celt, Spain_LBA, Spain_LIA, Spain_MBA, Spain_Me, Spain_Visigoth, Spain_Visigoth_Barcelona, Spain_Visigoth_Granada, Sweden_BAs, Sweden_BACs, Sweden_Baxs, Sweden_EaVikings, Sweden_FBCs, Sweden_Gotland_PW_Baxs, Sweden_Gotland_Hemmor_PW_Baxs, Sweden_Gotland_Hemmor_PW_Bax_m, Sweden_Gotland_Vast_PW_Baxs, Sweden_Gotland_Vast_PW_Bax_m, Sweden_IAs, Sweden_IA_1s, Sweden_IA_2s, Sweden_L_Ns, Sweden_LNs, Sweden_LNBA, Sweden_Vikings, Switzerland_BellBeaker, Switzerland_EBA_1, Switzerland_EBA_2, Turkey_Alalakh_MLBA, Turkey_Arslantepe_EBA, Turkey_Arslantepe_LC, Turkey_C, Turkey_CamlibelTarlasi_LC, Turkey_EBA, Turkey_Hellenistic, Turkey_IA, Turkey_MBA, Turkey_TellAtchana_MLBA, Ukraine_Alexandria_MBA, Ukraine_BA_Catacombs, Ukraine_Chernyakhivs, Ukraine_Chernyakhiv_os, Ukraine_Cimmerianss, Ukraine_Cimmerians_o1s, Ukraine_EBA_GlobularAmphora, Ukraine_EBA_Yamnaya, Ukraine_Eneolithic_Trypillia, Ukraine_GlobularAmphora, Ukraine_IA_WesternScythian_o1s, Ukraine_Mes, Ukraine_Scythians, Ukraine_VertebaC_MLTrypillia.

Both tests aim to test which ancient populations are significantly closer to the Test population.

Detection of genetic outliers by *f4* statistics

Given implied by the unsupervised admixture results substantial genetic diversity of the studied individuals from IA and MA periods we attempted the identification of genetic outliers for each time period relative to the overall ancestry of the corresponding population. To achieve this following Antonio et al. 2019, we took a recursive procedure to identify the most significant outlier sequentially. First in each round, we calculated the f4 statistics for each sample in the form f4(Test individual, contemporaries; ancient source, Mbuti), where "contemporaries represent all other (n-1) samples from the same period as the test individual (here IA and MA

populations), and “ancient source” is (i) one of five distinct ancient populations that represent typical ancestries: WHG, Anatolia N, Morocco Iberomaurusian, Russia Yamnaya Samara, Iran Ganj Dareh N, Natufian, and (ii) one of ancient population identified by previously presented f4-statistics with which IA and MA populations share more alleles than with the rest of their contemporary populations. We tested the following ancient sources: Denmark_IA, Norway_IA, Lithuania_BA, Latvia_BA, and respectively IA or MA populations. No outliers with significant zscore > 3 were identified.

Admixture modelling with qpAdm

We carried out qpAdm analyses on pseudo-haploid genotypes using qpAdm in the ADMIXTOOLS software with the option “allsnps: YES”. We considered only models with pvalues>0.05, which indicates a small deviation of data from the model expectation, to be consistent with the data. In case of two-way models we considered only the models with feasible admixture proportions. We adhered to the recommendation that the p-values are not comparable across models, because different source populations differ in sample size and coverage (i.e., the number of SNP sites covered by at least one read).

We performed admixture modelling of the studied ancient individuals for each time period separately. For each time period, we performed two rounds of admixture modelling. First we tested one-way models with only one source population, where the source is an ancient population earlier than or of the same age as the target population. We then tested two-way admixture models with the studied samples in the preceding period as one source and another ancient population as the other source.
Our modelling strategy assumes continuity in the genetic makeup, in our case of people living in the south Baltic region since the Bronze Age and greatly reduces the search space.

Although other two-way models which do not involve the preceding local population may also provide good fits, we consider them to be biologically unlikely and thus did not test those models.
As the qpAdm analysis results depend on the selection of the outgroup populations we selected the well tested and described ANC17 right outgroup set from Antonio et al. Science 2019.

The ANC17 right ourgroup panel consisted: Anatolia_N (25), CHG (2), EHG (4), ElMiron (1), GoyetQ116-1 (1), Iran_Ganj_Dareh_N (3), Jordan_PPNB (1), Kostenki14 (1), MA1 (1), Morocco_Iberomaurusian (6), Mota (1), Natufian (6), Ust_Ishim (1), Vestonice16 (1), Italy_Villabruna (1), WHG (6), Russia_Yamnaya_Samara (9).

Numbers in parentheses following each population indicate the number of individuals included in the group. To ensure robustness of the results to pulations with fewer than 50,000 SNPs available for analysis and those with less than 3 individuals were not considered for qpAdm modelling, due to lack of power to reject the admixture models.

To avoid spurious results due to low number of individuals or SNPs for one-way models we have excluded from modelling target populations with less than 4 individuals, leaving for MA group 12 populations, and for the two-way models we have excluded target populations with less than 5 individuals limiting the MA group to 8 populations.

qpAdm model competition

When more than one one-way admixture model produced a valid fit, we adopted a model competition approach, by adding the each source population to the right outgroup set. If after placing the competing source population the statistical significance dropped below p=0.05 this could suggest that the target population shares more alleles with this source than the one placed in the left group.

Also in the case of two-way models of IA_X populations of Hungary_Szolad_MigrationPeriod + X, when a IA_X population could be modelled as X= MA_X population or preceding earlier BA population we performed analogous model competition tests.

SUPPLEMENTARY NOTE 9. Y-CHROMOSOME HAPLOGROUP INFERENCE

Haplogroup determination

Data preprocessing was carried out as described in SUPPLEMENTARY NOTE 5. BAM files were used as input to Yleaf v2.1 software to determine Y-chromosome haplogroups. Yleaf v2.1 uses the haplogroup nomenclature of the International Society of Genetic Genealogy (ISOGG) v. 14.111. Prediction was done with the following parameters: the minimum number of reads for each base to be called was set to 2 (-r 2), the minimum quality for each read was set to 30 (-q 30) and the percentage of base calls at each position should be at least 90% (-b 90). Each sample output file was manually analyzed to recover the most informative positions. Due to potential sequencing errors and residual deamination lesions we filtered out C->T and G->A sites that were below 6x coverage. We also removed sites that did not agree with the phylogenetic context of the haplogroup classification which could be in part private mutations or contamination. We didn’t take into account markers and haplogroups highlighted with special characters i.e. “^”, ”^^” ,”~” as they don’t yet meet ISOGG quality guidelines and need more evidence to be confirmed.
Haplogroups were assigned as the most derived mutation observed in a particular sample. The lack of base calls at specific positions within clades was resolved by imputation of an allele either as ancestral or derived to all upstream alleles in the haplogroup based on the phylogenetic context.
For more detailed information about found Y-chromosome SNPs refer to Table S3.

Results

Here we want to comment on the haplogroup assignment of sample PCA0499. Based on the derived SNPs found for this individual there were 3 possible haplogroups that could be assigned. Namely J2a1a1b2a1b, J2a1a1b3 or J2a1a4b. As we couldn’t distinguish the correct haplogroup we decided to assign it to J2a1a as it is the most recent common ancestor of all three possible haplogroups.
Our results highlight the prevalence of haplogroups I1/I1a during the Iron Age in the territory of contemporary Poland. These haplogroups were found to be the most frequent haplogroups in each studied location ranging from 35.3% in Pruszcz Gdański to 57.1% in Masłomęcz and totalling to 41.3% in the whole IA group. Six individuals could be assigned to a more detailed Y-chromosome haplogroup representing three different I1a clades. Two males, one from Masłomęcz (PCA0102) and one from Pruszcz Gdański (PCA0480), belonged to the I1a1 clade and were assigned as I1a1-L22 and I1a1-P109 respectively. Next, a male from Masłomęcz (PCA0100) belonged to the I1a2 clade and was assigned as I1a2-Z2040. Finally, three males all from Kowalewko belonged to the I1a3 clade and shared the same marker I1a3-L1237. The remaining lineages had the following frequencies R1b:13.0%, G2a:11.0%, R1a:8.7%, Other:6.5%, E1b:4.3%, I2a:4.3%, N1a:4.3%, J2a:2.2%, J2b:2.2%, Unk:2.2% as shown in Figure 3a. Due to insufficient coverage and haplogroup assignment, individuals PCA0524,

PCA0037, PCA0494 were grouped as "Other" for clarity in presenting the results. We were unable to assign haplogroup to one male (PCA0455) and thus classified him as unknown (Unk). The MA period was dominated by the presence of R1a haplogroup. 65 individuals belonged to the R1a lineage which comprises 57.5% of the MA group. R1a-S198 was the most frequent marker reaching 70.8% within the R1a individuals. Two downstream clades of R1a-S198 stood out with very high frequencies, namely R1a-M458 (44.6%) and R1a-S204 (23.1%). Two subclades of R1a-M458 were observed. First, R1a-L260, was found in 12 individuals of which three were further derived for the R1a-YP256 mutation. Second, R1a-L1029, was detected in nine individuals that belonged to this haplogroup. Within the R1a-S204 clade, 13 individuals were positive for the CTS1211 marker. Downstream of the CTS1211 we detected eight males derived for the CTS3402 marker and two males were further derived for the L1280 marker. Remarkably, the R1a-S204 clade was not observed in the IA individuals.

The remaining haplogroups in MA group had the following frequencies: Other:11.5%, R1b:7.1%, I2a:5.3%, E1b:4.4%, R1:4.4%, Unk:2.6%, I1:1.8%, I1a:1.8%, J2a:1.8%, N1a:1.8% as shown in Figure 3a. Due to insufficient coverage and less precise haplogroup assignment, individuals PCA0230, PCA0236, PCA0240, PCA0241, PCA0298, PCA0318, PCA0338, PCA0358, PCA0369, PCA0385, PCA0392, PCA0421, PCA0519 were grouped as "Other" for clarity in presenting the results. We were unable to assign haplogroup to three males (PCA0337, PCA0360, PCA0402) and thus classified them as Unk.

SUPPLEMENTARY NOTE 10. MITOCHONDRIAL DNA HAPLOGROUP INFERENCE

MtDNA genome assembly and haplogroup prediction

Data preprocessing was carried out as described in SUPPLEMENTARY NOTE 5. Consensus fasta sequences for haplogroup prediction and sequence analyses were generated with FreeBayes ver. 1.0.2–33-gdbb6160. To assemble complete consensus mtDNA sequences, we applied the following quality requirements: minimum coverage per base >=3, missing nucleotide count < 5%, no missing nucleotides in the HVS-I sequence, base call supported by at least the 3/5 majority of reads.

Haplogroups were assigned based on complete mtDNA sequences using HaploFind with respect to Phylotree build 17 (http://www.phylotree.org/). Only samples with a haplogroup score ≥0.8 were used in downstream analyses.
