# Supplementary figures and images for: Genetic history of East-Central Europe in the first millennium CE
Source: Genome Biol. 2023 Jul 24;24:173. doi: 10.1186/s13059-023-03013-9 (PMC10364380; doi:10.1186/s13059-023-03013-9)

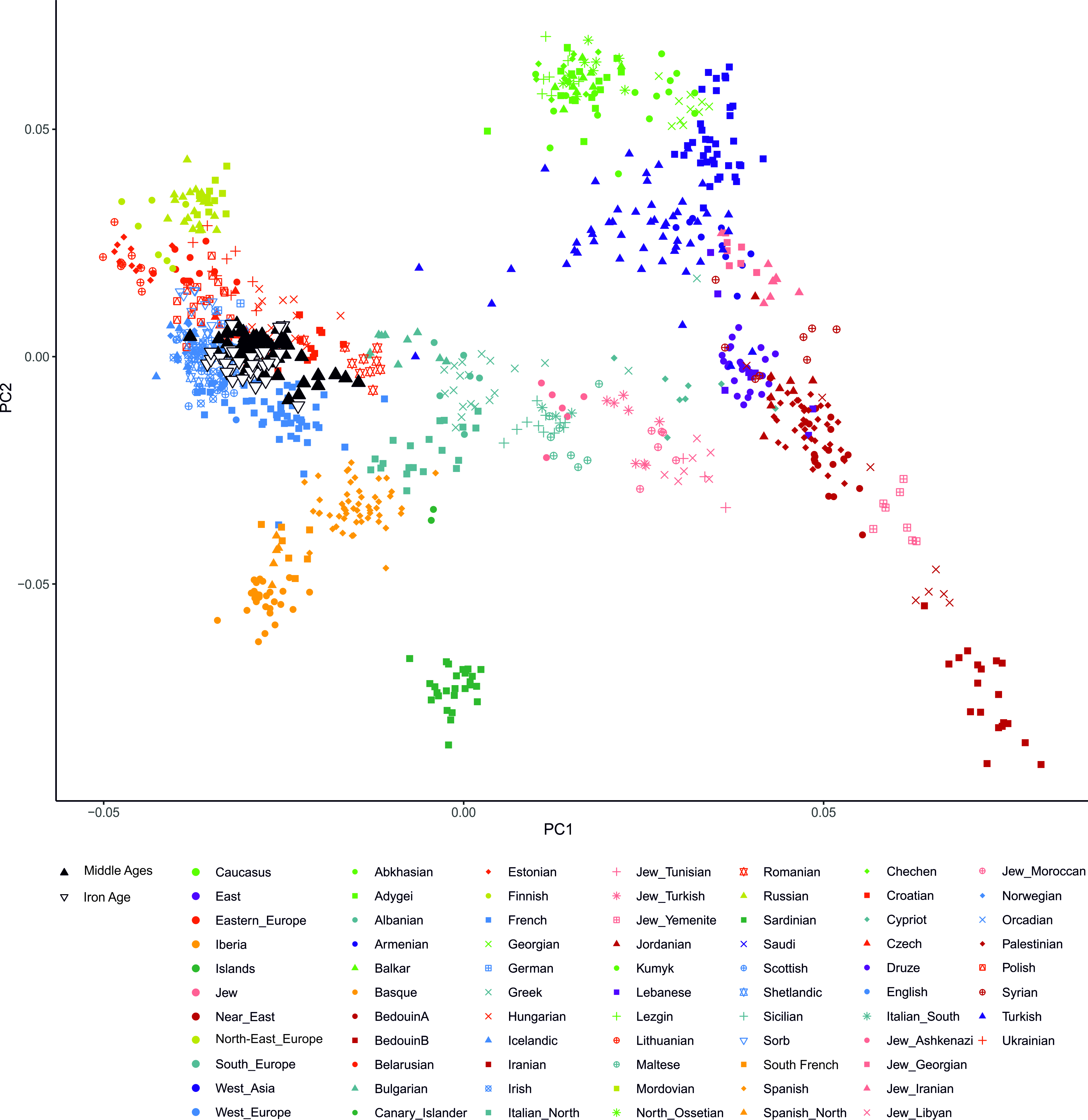

Supplement: Supplementary file 3 — Additional file 3: Fig. S14. PCA embedding of the studied ancient individuals and present-day West-Eurasians. Ancient individuals were projected onto the first two eigenvectors of a PCA based on contemporary West Eurasians. [file 13059_2023_3013_MOESM3_ESM.tif]

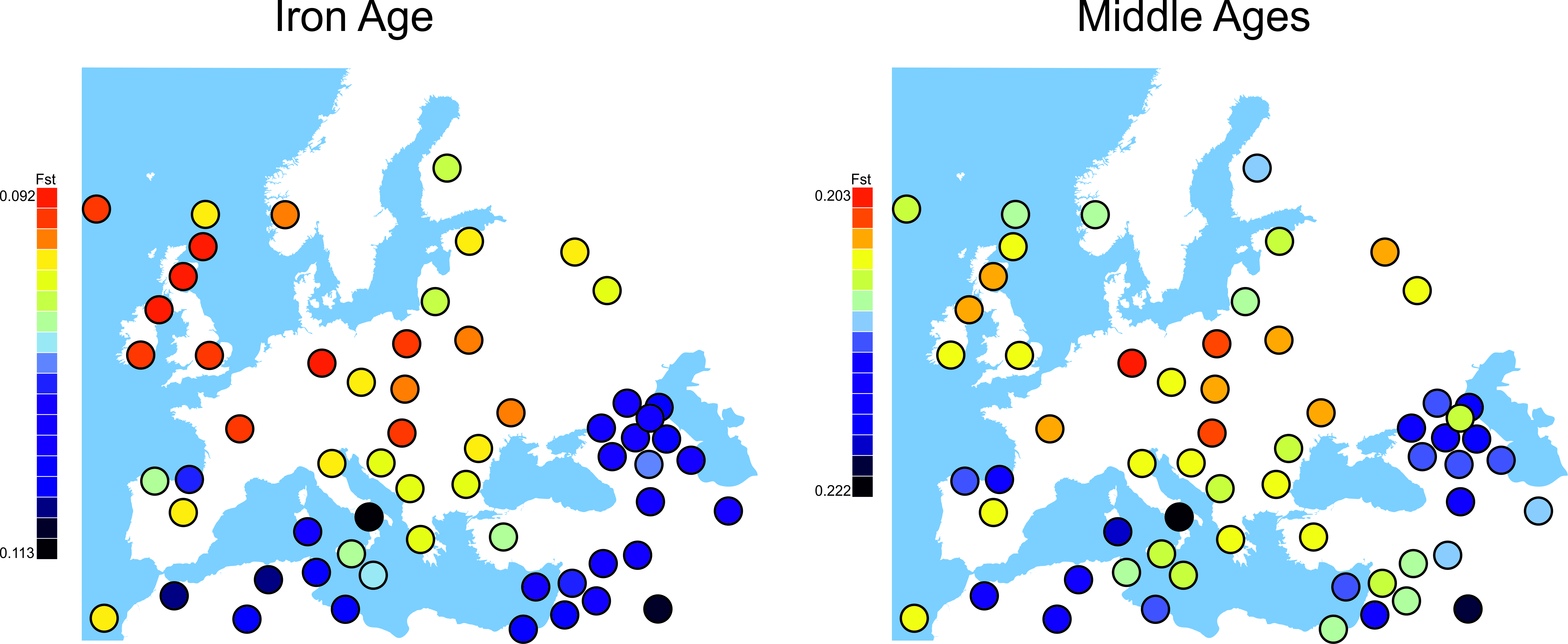

Supplement: Supplementary file 4 — Additional file 4: Fig. S15. Genetic distances between studied ancient individuals and present day West Eurasians. Colour coding reflects the genetic distance as measured by the Fst coefficient between ancient individuals and present-day ones separately for the IA and MA group. [file 13059_2023_3013_MOESM4_ESM.tif]

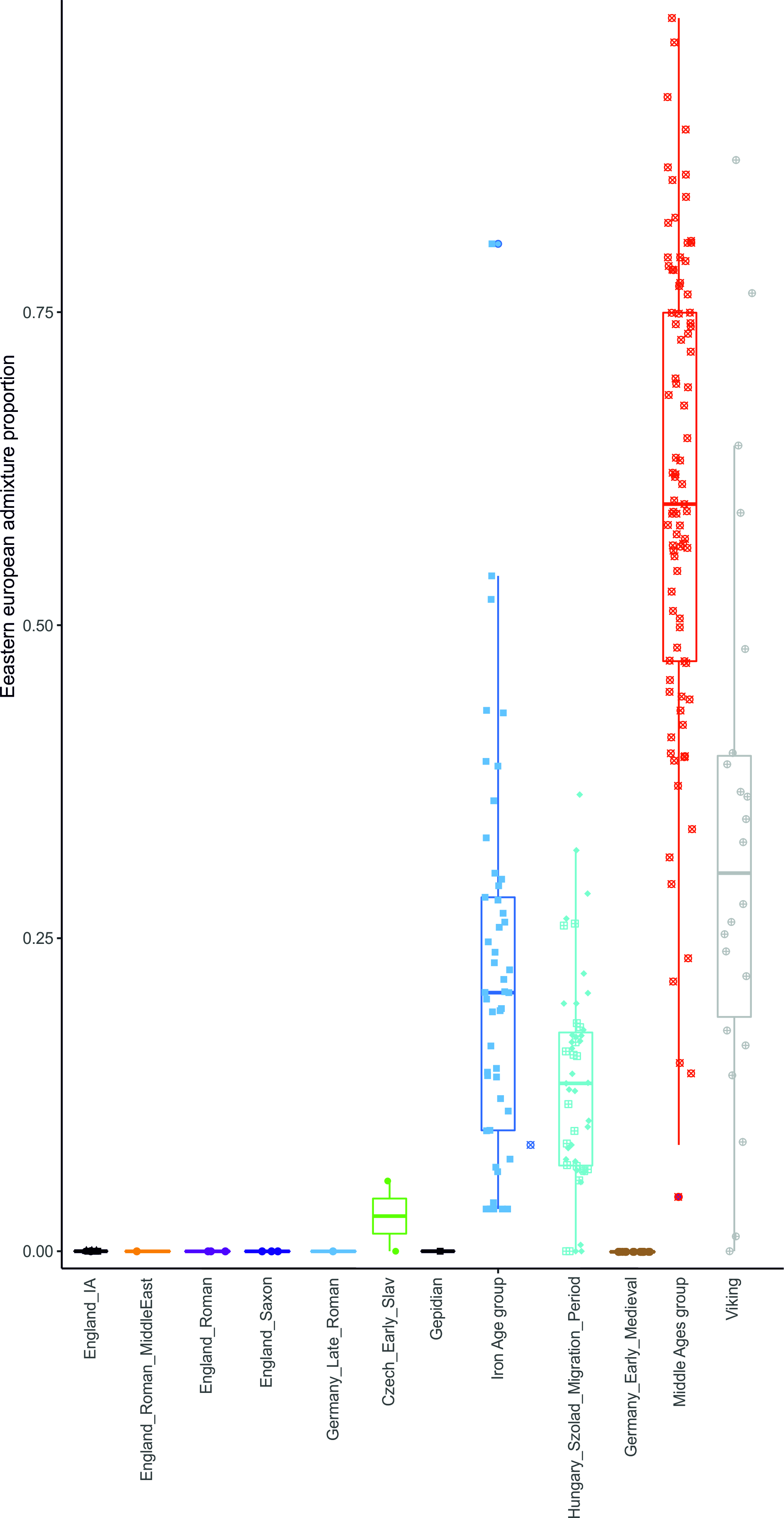

Supplement: Supplementary file 5 — Additional file 5: Fig. S16. Estimated Eastern-European admixture proportions in ancient populations. Shown is the result of unsupervised ADMIXTURE run at K=9 corresponding to the genetic components presented on Fig. 1D and fig. S17. [file 13059_2023_3013_MOESM5_ESM.tif]

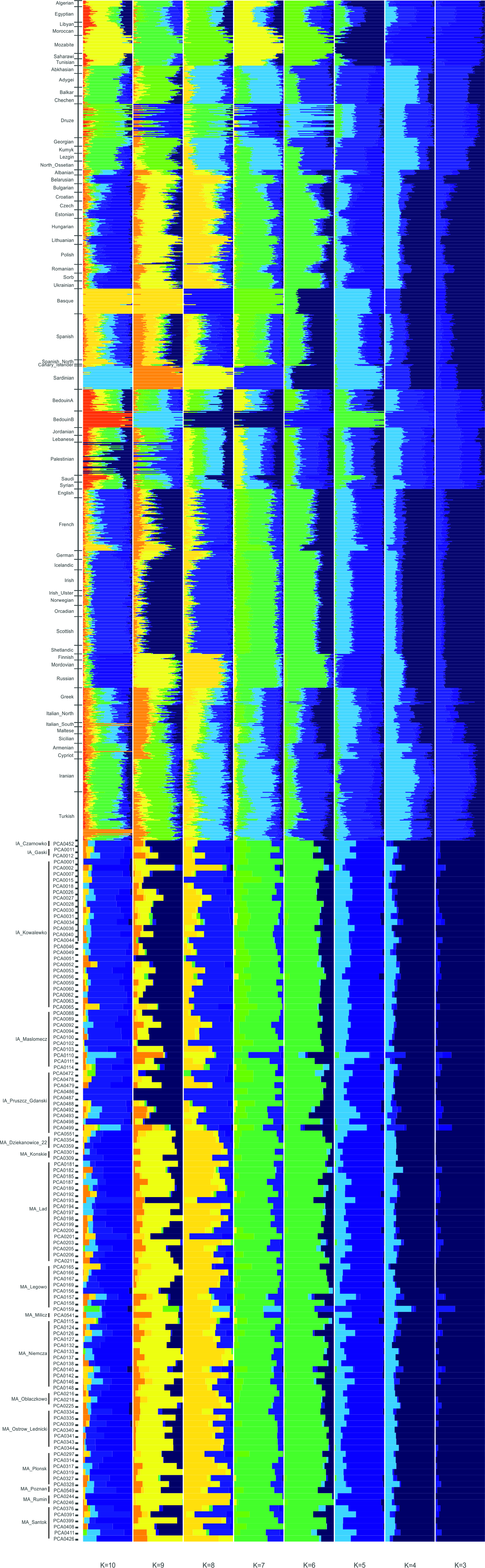

Supplement: Supplementary file 6 — Additional file 6: Fig. S17. Estimated ancestry proportions for present-day populations from Human Origins dataset. Shown are unsupervised ADMIXTURE results for K=9. [file 13059_2023_3013_MOESM6_ESM.tif]

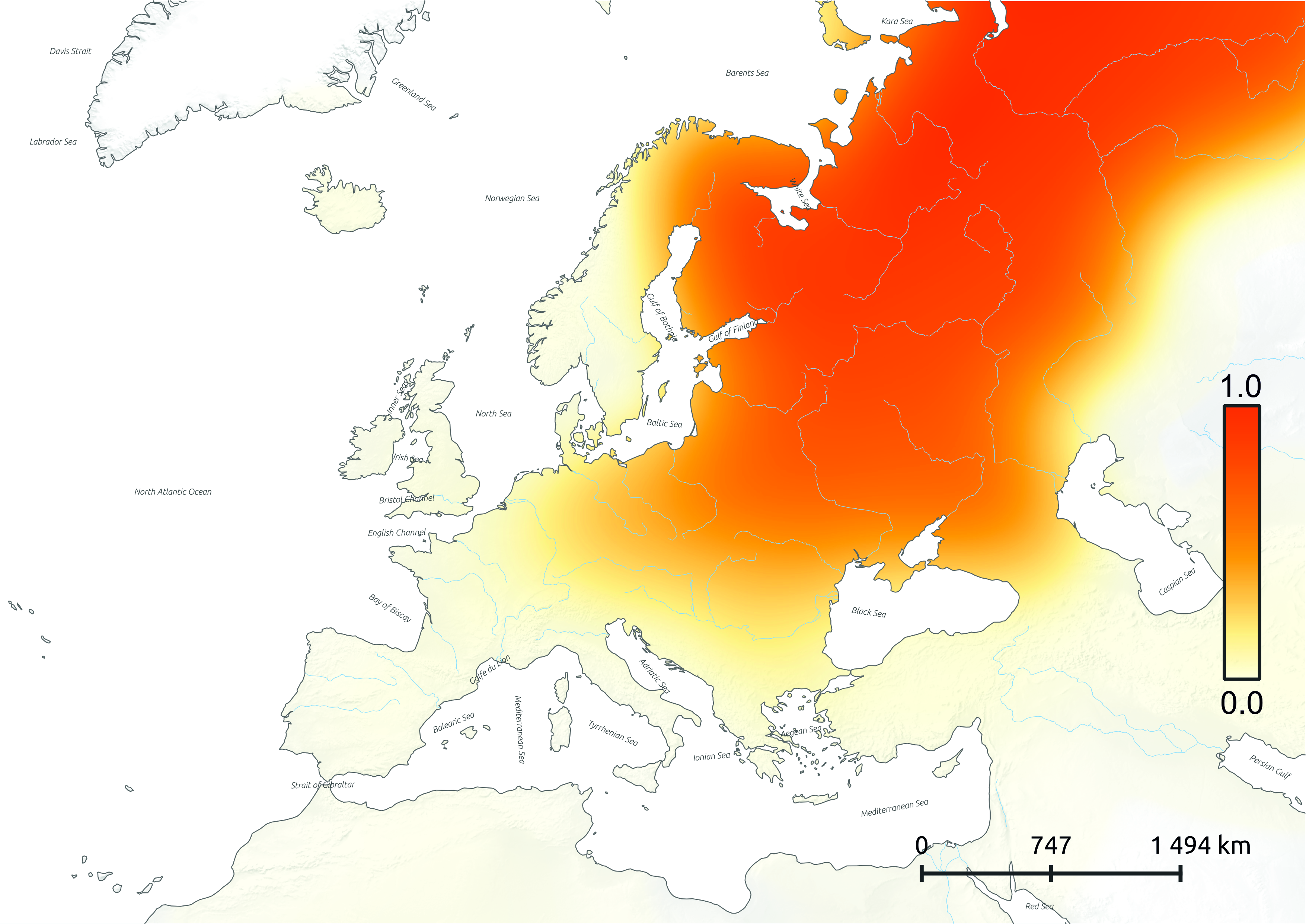

Supplement: Supplementary file 7 — Additional file 7: Fig. S18. Geographical distribution of Eastern-European admixture in present-day European populations. Shown is the result of unsupervised ADMIXTURE run at K=9 corresponding to the genetic components presented on Fig. 1D. [file 13059_2023_3013_MOESM7_ESM.tif]

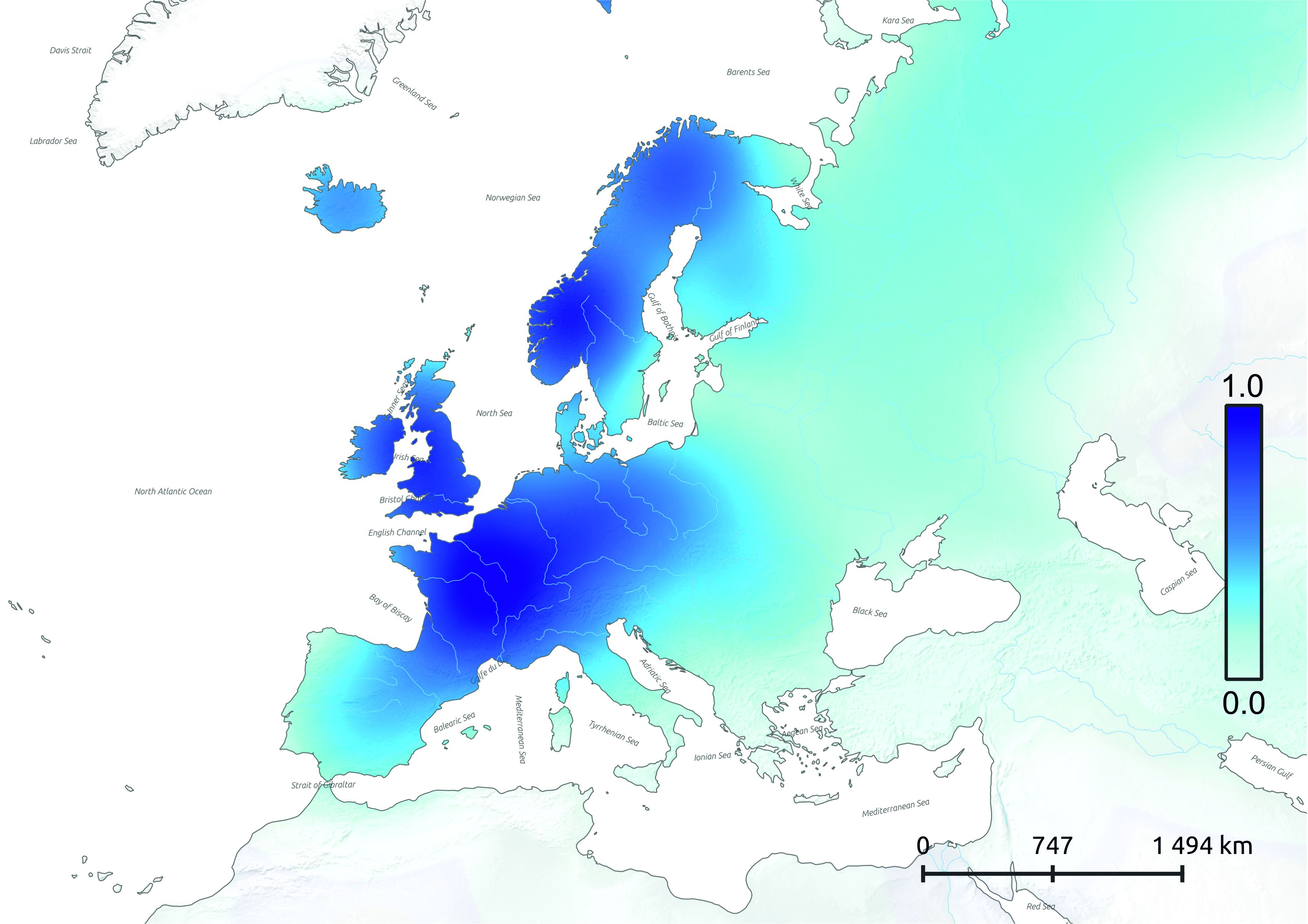

Supplement: Supplementary file 8 — Additional file 8: Fig. S19. Geographical distribution of North-Western admixture in present-day European populations. Shown is the result of unsupervised ADMIXTURE run at K=9 corresponding to the genetic components presented on Fig. 1D. [file 13059_2023_3013_MOESM8_ESM.tif]

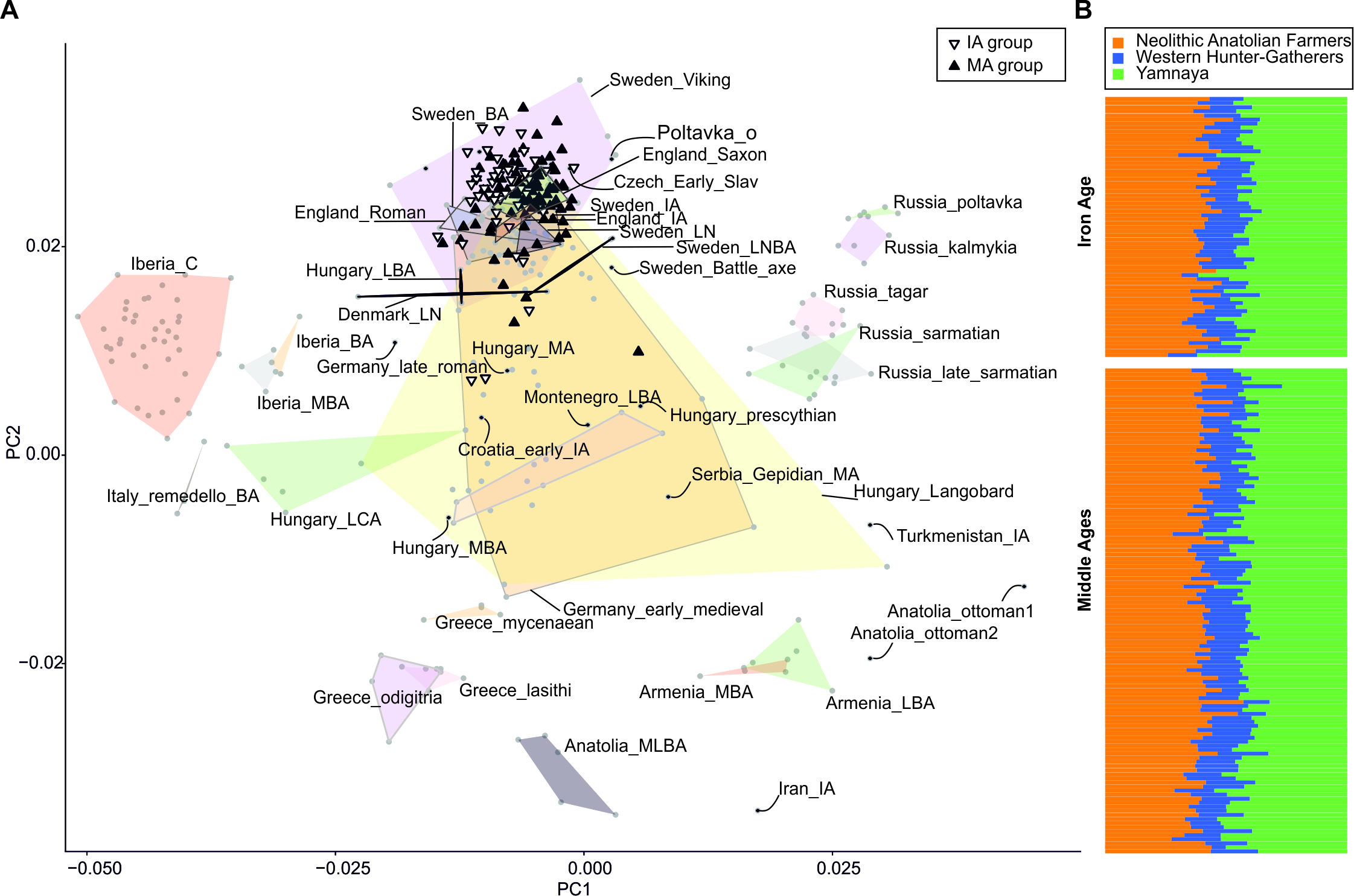

Supplement: Supplementary file 9 — Additional file 9: Fig. S20. Spatiotemporal distribution of samples and their genetic affinities to ancient populations. (a), PCA embedding of the studied samples and other ancient post-LN samples from Western Eurasia. Polygons highlight the space occupied by the samples from each group. (b), supervised ADMIXTURE (K=3) for IA and MA individuals modelled as mixtures of Neolithic Anatolian Farmers (orange), Western Hunter-Gatherers (blue) and Yamnaya (green). [file 13059_2023_3013_MOESM9_ESM.tif]
