# Supplementary material for: Genetic history of East-Central Europe in the first millennium CE
Source: Genome Biol. 2023 Jul 24;24:173. doi: 10.1186/s13059-023-03013-9 (PMC10364380; doi:10.1186/s13059-023-03013-9)
Supplement: Supplementary file 10 — Additional file 10. Author and affiliation list for members of the Polish Archaeogenomic Consortium. [file 13059_2023_3013_MOESM10_ESM.docx]

**Author and affiliation list for members of the Polish Archaeogenomic Consortium**

Mirosław Andrałojć

REFUGIUM Archaeological Laboratory, Poznan, Poland

Krzysztof Błaszczyk

ARTEFAKT Archaeological Company, Radomsko, Poland

Jan Chochorowski

Institute of Archeology, Faculty of History, Jagiellonian University, Krakow, Poland

Maciej Chyleński

Institute of Human Biology & Evolution, Faculty of Biology, Adam Mickiewicz University, Poznan, Poland

Lukasz Ciecierski

Institute of Bioorganic Chemistry Polish Academy of Sciences, Poznan, Poland

Lidia R. Cymek

Pomeranian University in Slupsk, Poland

Józef Dobosz

Faculty of History, Collegium Historicum, Adam Mickiewicz University, Poznan, Poland

Krzysztof Gorczyca

District Museum in Konin, Poland

Izabela Ignatowicz

The Jan Dekert Lubusz Museum, Gorzow Wielkopolski, Poland

Tomasz Janiak

Museum of the Origins of the Polish State in Gniezno, Poland

Tomasz Jasiński

Faculty of History, Collegium Historicum, Adam Mickiewicz University, Poznan, Poland

Sławomir Kozieł

Department of Anthropology, Institute of Immunology and Experimental Therapy Polish Academy of Sciences, Wroclaw, Poznan

Marcin Krzepkowski

Regional Museum in Wagrowiec, Poland

Andrzej Krzyszowski

Archaeological Museum in Poznan, Poland

Andrzej Legocki

Institute of Bioorganic Chemistry Polish Academy of Sciences, Poznan, Poland

Anna Myszka

Center for Ecology and Ecophilosophy, Cardinal Stefan Wyszyński University, Warsaw, Poland

Wioletta Nowaczewska

Department of Human Biology, University of Wrocław, Poland

Paweł Pawlak

No affiliation

Mirosław Pietrzak

Archaeological Museum in Gdansk, Poland

Franciszek Rożnowski

Pomeranian University in Slupsk, Poland

Katarzyna Schellner

District Museum in Konin, Poland

Barbara Stolpiak

No affiliation

Marzena Szmyt

Archaeological Museum in Poznan, Poland

Krzysztof Szostek

Institute of Biological Sciences, Cardinal Stefan Wyszyński University, Warsaw, Poland

Agnieszka Szyca

Institute of Human Biology & Evolution, Faculty of Biology, Adam Mickiewicz University, Poznan, Poland

Jakub Śliwiński

ALTAMIRA Archaeological Company Jakub Śliwiński, Sieradz, Poland

Małgorzata Talarczyk-Andrałojć

REFUGIUM Archaeological Laboratory, Poznan, Poland

Iwona Teul

Pomeranian Medical University in Szczecin, Poland

Dawid Trzciński

Institute of Human Biology & Evolution, Faculty of Biology, Adam Mickiewicz University, Poznan, Poland

Krzysztof Wachowski

No affiliation

Anna Wrzesińska

Museum of the First Piasts at Lednica, Dziekanowice, Poland

Jacek Wrzesiński

Museum of the First Piasts at Lednica, Dziekanowice, Poland

Daniel Żychliński

THOR Archaeological Research Laboratory, Gniezno, Poland
