# Supplementary material for: Genetic history of East-Central Europe in the first millennium CE
Source: Genome Biol. 2023 Jul 24;24:173. doi: 10.1186/s13059-023-03013-9 (PMC10364380; doi:10.1186/s13059-023-03013-9)
Supplement: Supplementary file 11 — Additional file 11. Peer review history. [file 13059_2023_3013_MOESM11_ESM.docx]

**Review history**

**First round of review**

**Reviewer 1**

Stolarek et al. present an extremely impressive dataset of ancient individuals from present-day Poland, dated to the Iron and Middle Ages, in order to study the genetic origin of the Slavs. They use an interesting indirect approach of ancestry determination by comparing the populations who lived in the region not directly before the appearance of the Slavs, but instead ~500 years before that, with Middle Ages Slavs. By analysing their genomes, they suggest that the Middle Ages Slavs were a "genetic continuation of the autochthon IA population". Unfortunately, I do not believe that the presented analyses support such conclusion of local continuity from the possibly admixed genomes of the IA individuals, and I am sadly disappointed that with such substantial amounts of data, no further analyses past PCA, ADMIXTURE, some few (poorly presented) f4-statistics, and uniparental haplogroups were done. In 2022 with such amounts of data, in my opinion, this is not to be expected for a journal such as Genome Biology, especially because all of the methods used require low amounts of data, and none provide statistically-supported quantitative results (e.g. ADMIXTURE vs qpAdm). The origin of the Slavs, like the authors say, is such an important and interesting subject, and so I really want to see this project succeed and the research on this population published. I hope the authors can critically accept my comments and improve them in a more in-depth, and more clear, study on these samples.

Specifically, my major concern is that I did not find any presented convincing evidence that the IA and MA populations are related in any way, so any claim of continuity has no foundation in the data. Using similar ancestry proportions to infer continuity of populations is incorrect. The MA population could as easily correspond to a non-local population with similar ancestry profiles who migrated there. I think the authors should not rely on the heavy use of ADMIXTURE to support any definite claims of origin, especially when only modern populations are used as indirect proxies. This software is usually only used in ancient DNA research as a guideline for downstream analysis, because it can only produce qualitative results and does not consider population histories. Furthermore, the use of modern haplogroup data to infer past events cannot overcome conclusions based on existing ancient haplogroup data.

More detailed concerns are in regards to the poor presentation of f4-statistics results (no f4 or Z value is present in the MS or SM or STables), which cannot be confirmed as to whether they produced any data supporting the authors' claims or not, and which from a purely visual interpretation of the figures, seem to suggest too small f4 values that might in fact not clearly support them.

One of my main suggestions is to re-analyse the data by looking at it into more detail, such as possibly identifying outlier individuals in IA and MA that could represent the local IA populations, and then using qpAdm modeling to see if a local model of continuity and admixture is the only valid result, or if the MA population could be modelled as other ancient populations (including as 1-way models).

My detailed comments are below. Note that line numbers mentioned are approximate, as they don't properly align with the text in the manuscript.

Abstract, Line 10: To help the reader, I think it would help to specify that these immigrant IA populations belonged to the Wielbark culture.

Results, Studied group description: There is no information regarding terminal deamination in Table S1. Also, are the numbers of nuDNA reads total reads of unique aligned reads to the human genome? And although the authors mention that they "performed a whole-genome analysis of representatives of the IA Wielbark and MA Slav cultures.", in Table S1 there are plenty of IA individuals that are not attributed to the Wielbark culture, even though the manuscript says all sites are Wielbark. This needs to be clarified. Lastly, the authors mention that 197 samples produced genome-wide data, but on Table S1 only 135 seem to show that.

Could it be that the IA individuals studied are not only from the Wielbark culture, but also from others (likely local)? If this is the case, it would really help in the interpretation and presentation of the results to always specify which IA population is being discussed. Or, is it even possible to separate the IA and MA individuals into different groups (for example using qpWave?), that could then be used independently for further analyses? Based on the ADMIXTURE plot in 1D it seems like the populations are fairly variable (although the very-low coverage of some individuals could also play an important role there), and maybe some outliers are not Wielbark, but instead locals (maybe mostly females?).

Results, Studied group description page, line 25: Please specify the conditions to qualify data as "suitable for further analyses". Is it just the 10.000 SNPs threshold mentioned in the Methods section?

Results, Studied group description page, line 32: Table S1 seems to be missing this information. Also, please clarify what the purple colored (at least in my downloaded version of the document) columns labeled as "mt / Whole Genome Enrichment Method / Y" correspond to. Are they all about enrichment?

Results, IA- and MA-group affinities page 1, lines 36-40: As there are more than one analysis dataset, please mention which one was used (HO, POPRES or ancestry-restricted).

Results, IA- and MA-group affinities page 1, line 49: Please present the other ADMIXTURE plots for the remaining K values in the SM.

Results, IA- and MA-group affinities page 2, lines 1-3: If these results are based on ADMIXTURE, the authors need to reword these sentences. None of the 9 admixture components presented represents the MA group, therefore it cannot be said that another group has a fraction of another who is not one of the defined ancestries. They might share similar structure or ancestry profiles, which does not mean that they are closely related. Also, the word "significant" must be restricted to statistically-backed results, which is not the case for ADMIXTURE and PCA analyses.

Results, IA- and MA-group affinities page 2, line 12: Please mention which dataset was used (HO, POPRES or ancestry-restricted).

Results, IA- and MA-group affinities page 2, line 33: The mentioned f4 statistic is not the same shown in Fig. 2c, please correct the required one. Also, please mention f4 values and Z-scores, and whether the results being discussed were statistically significant. I was not able to find the value results of these f4-statistics (plus Z-scores and SNP numbers), and nowhere in the manuscript or SM is there a mention of the used threshold of significance for drawing conclusions. By looking at Fig S21, Fig. 2c, and the Methods, there is no mention of significance, and there is no mention of what the bars represent. Even if 2SE, many tests overlap with the 0 line, many others have the error bars close to it, and overall the presented range of values of f4 (-0.00075 to 0.00050) suggests that only a handful of the tests might be statistically significant, if any at all.

Results, IA- and MA-group affinities page 2, line 39: Again, the mentioned f4 statistic is not the same shown in the mentioned figure [ f4(X, Western Europeans; Norwegians, Mbuti) versus f4(Mbuti, X; Western Europeans, Norwegians) ].

Results, IA- and MA-group affinities page 2, lines 44-48: Not completely necessary, but it would be great to have the SNPs in Table S2 organised and identified by the phenotypical characteristic they relate to.

Results, IA- and MA-group affinities page 2, last paragraph: I am unfortunately not convinced of the "locality" of these claims due to 2 points, and that is my main critique of this manuscript:

1)the ancestries of the IA and MA populations based on PCA and ADMIXTURE do seem to be different but it is not proved with any analyses that there is any kind of IA-MA continuity or relationship. Of course there is no local IA data to more directly test that, but the authors could have at least tried to use local Bronze Age individuals. Also, continuity is a hard concept to prove in paleogenomics, but analyses such as qpWave and competition-based qpAdm with other ancient populations might help understand these processes.

2)The MA affinities to modern East-Central populations could equally represent a MA group arriving from a different region, admixing, and eventually establishing themselves in the region so that modern populations share similarities with them. No reasoning is presented to support the belief that they represent the local IA population instead of another eastern group.

One suggestion I have is that the authors could use qpAdm to test whether Wielbark can be modelled as MA plus for example Netherlands BA or IA (Patterson et al. 2022), or also Hungary Longobards who they seem to suggest are similar to the population who migrated North-to-South through Poland, but for which admixture was not thoroughly tested. That seems to be the most straightforward test to see if MA would represent the local population who mixed with the incoming Longobards to represent the Wielbark.

As I mention in another comment, it could be interesting to try to identify different clusters of individuals within the IA (and MA), as some of those might be more direct representatives of the local IA population. Perhaps some of those local individuals moved over to live with the Wielbark groups, and was therefore inhumated instead of cremated, giving you direct access to local IA individuals. This would only be an hypothetical situation, but if indeed outliers are detected, it can be argued (based on the MT and Y data you have) that they could be locals.

Uniparental marker analysis, overall: It would useful to compare the IA data with existing ancient Polish individuals before the Iron Age.

Uniparental marker analysis, page 1, lines 17-25: It seems to be a substantial leap to use as evidence for continuity, locality, and admixture, the presence of Y-hg R1a in large frequencies in MA but with some small cases in the IA. Haplogroup Y-hg R1a was already present in the Poland in the Late Neolithic and BA as well (see Olalde et al. 2018; Fernandes et al. 2018; Malmstrom et al. 2019), although at small frequencies (3/17 Chalcolithic and Bronze males). How do the authors explain such an explosion to eventually represent 57.5% of all Y-hg in the MA? Some local pressure is one possibility, but a migration of external populations is another, and perhaps more likely. R1a is also found in IA Czechia, Slovakia, Estonia, Mongolia, and it is the major Y-hg found in the Srubnaya (12/14 males) and Sintashta (23/31 males) cultures. Sample bias is also a possibility, but in the end I don't believe this can be used to inform locality and continuity of the MA group.

Uniparental marker analysis, page 1, lines 26-30: Y-hg R1b has been present in Poland before the IA and MA. During the Beaker period and BA it is the overwhelming Y-hg (Linderholm et al. 2020), so it cannot be associated specifically with Northwestern European populations and a migration from the West. If the authors are referring to a very specific sub-clade, they should mention it like they did for R1a.

Discussion, page 1, line 25: I don't think the author presented any evidence of admixture with local populations using the genome-wide data. ADMIXTURE plots with similar ancestries to other populations cannot be understood as evidence of admixture with those populations. Those ancestry profiles could, for example, be present in some of the individuals who migrated from the Northwest with the Wielbark. They can also represent admixture with other populations outside of the territory analysed. Above I suggested possible analyses to look into this.

Discussion, page 1, lines 27-30: Please clarify this sentence. As it currently is, it suggests that the admixture seen in the Wielbark genomes is the same that existed in the MA group and in the Western Slavs. One of your main findings was that IA and MA had substantially different admixture profiles, so here it sounds like you are saying that instead they were the same. Also, Western Slav populations are mentioned in here for the first time, without any context during the presentation of the results, so it is not clear who they refer to.

Discussion, page 1, line 33: What do you mean by "contemporary"? Which other Medieval East-Central European ancient populations does this refere to? From Fig. S16, no other analysed ancient population has a proportion distribution of Eastern ancestry even close to that of the MA group.

Discussion, page 1, lines 48-59: Again, the idea that this high R1a frequency appeared in the MA cannot be discarded as having had an origin outside of this region. The authors are comparing their ancient MA data with modern populations, but the other way around does not provide in any way evidence of origin in the past. If anything, it is evidence of local continuity of haplogroup distributions to modern times. One can more easily go forward in time with these speculations, but not back.

Methods: Were reads trimmed at the terminal ends, to exclude deaminated positions from downstream analysis (especially for hg determination)? If not, were the libraries selected for deeper sequencing UDG treated?

**Reviewer 2**

This paper by Stolarek et al. attempts to use ancient DNA data to investigate when historical Slavs first entered the territory of present-day Poland. It uses autosomal SNPs, mtDNA and Y chromosomes as genetic markers, comparing a large sample of Iron Age (IA) and Middle Age (MA) individuals to modern European populations using PCA and model-based clustering analysis. Most notably their results reveal that IA individuals show more similarity to modern northwestern European populations while MA individuals more closely resemble modern eastern European populations.

The primary conclusion of this paper is 1) there is genetic continuity between IA and MA groups in present-day Poland and 2) there were two migrations from Northern Europe and Western Europe to present-day Poland in the first millennium.

The paper provides a large number of ancient genomes (almost 500 genomes total, almost 200 of which have sufficient endogenous DNA and sequencing data for downstream analysis) from early and later Medieval eastern Europe, a region and period for which there is a paucity of existing published data. Generally the population genetic methods applied in the paper are done in an appropriate way (PCA, model-based clustering, FST and drift analysis [F4]); however, how the authors interpret the results of these analyses to form their conclusions is problematic. The conclusions the authors draw cannot be robustly inferred based on current evidence, and some important issues, particularly with regard to a) describing the generation of the data and b) nomenclature used to describe the affiliation of individual genomes, need to be resolved for this paper to be suitable for publication.

Major issues

1) The results of this paper do not support the two main conclusions.

1a) (Page 11, line 26-28, "(i) the population inhabiting present-day Poland in the MA was genetic continuation of the autochthon population living at the same territory in the IA". This appears to be a misunderstanding of what the Admixture results represent. Sharing same genetic components given by Admixture analysis using external reference populations doesn't necessarily mean the two groups actually share same genetic ancestry indicative of population continuity (Page 8, line 1-3, "numerous individuals from the IA group shared some fraction of their genetic ancestry with individuals from the MA group and contemporary East- Central European Slavic populations".) since clustering analysis is highly dependent on what references are used. It is necessary to provide a formal test (e.g. qpAdm, qpWave, Some kind of explicit model-testing framework like Approximate Bayesian Computation etc) that can clearly suggest there is sharing of genetic ancestry from one period to the next, as well as determine the degree of continuity.

1b) (Page 11, line 28-32, "(ii)during the first millennium the genetic makeup of East-Central European autochthones was affected by two major demographic events, the migrations of Northern Europeans in the 1st - 4th century and Western Europeans in 10th - 12th century"). There are a number of issues with this claim:
(i) This study only uses modern reference populations to orientate the past populations of interest and it is unknown if modern populations can reflect the contemporary genetic makeups of ancient individuals in the same geographic regions. For instance, the similarities between IA groups and modern northwestern European populations does not mean the IA groups have genetic similarities with ancient northwest populations. Comparison between contemporary groups are necessary to address the migration hypothesis. To obtain contemporary northern and western individuals, the authors can refer to (Margaryan et al. 2020; Gretzinger et al. 2022).
(ii) Even if the paper demonstrates there are genetic similarities between IA groups and contemporary northwest populations, this does not mean there was necessarily a migration into modern-day Poland during this period. The paper didn't not analyze any ancient individuals excavated in Poland that date prior to IA groups so there is no way to determine if there was actually a regional genetic change (which might have been contributed by the immigrants) within the IA periods. It is quite possible that the previous population looked much like the IA population, even if they both looked somewhat northern European in ancestry (due to a potentially much earlier migration, in either direction). Similarly, there is a large time gap between the IA and MA individuals (around 600 years), which is certainly long enough for many different demographic changes, and thus difference in genetic makeup between IA and MA individuals cannot be used as evidence to support any particular migration event (the shift in ancestry could have involved multiple pulses, undetected ancestry shifts or simply a long-term gradual change). In order to test the two hypothese, it is necessary to provide genetic data from Poland prior to the IA as well as between IA and MA. If it is impossible to get aDNA prior to the IA, strontium isotope analysis from the IA individuals might help to uncover if there was migration.

2) Specific criteria are needed to illustrate why some ancient individuals were called Slavs (Page 6, line 9) or Longobards (Page 10, line 30). Whether this determination was based on historical records or archaeological artifacts should be mentioned. Even if the criteria is provided, it is also highly problematic to assign ancient individuals to specific ethnic groups since we never actually know how people recognize themselves in the past. Identity is complex and it is insufficient to illustrate it using a single word. We have no idea (and will never know) if the people in this paper actually identified as "Slavs" or "Longobards". In this case, using time and space (geography) to describe ancient groups would be a much less problematic approach.

3) For the third PCA ancestry-restricted dataset (Page 12, line 29), it is hard to understand why specific language groups (Gaelic, Germanic, Slavic, Baltic and Norse) were used and who were assigned to these groups (are the assignments based on country)? The purpose of this PCA analysis should be addressed. Meanwhile, the terms are not really comparable since Gaelic and Baltic are much smaller groups compared with the Germanic and Slavic groups. Similarly, in the figure 2a, it is not reasonable to put language groups (Baltic, Finnish and Norse) and geographical groups (North-western Europeans and Eastern Europeans) together as references. These groups are not comparable.


Minor issues

4) (Page 11, "Ancient DNA analysis"). A clearer and comprehensive description of how samples were processed (e.g. How the aDNA were enriched for different autosomal and sex-specific markers) and analyzed is needed. It would be very hard to replicate the methods for data generation as described.

5) The labeling of this paper is not consistent. For instance, "East-Central" populations are mentioned (e.g. Page 8, line 2-3, Page 8, 14-15) several times, but in the legend of admixture plots there are only "Eastern populations". The annotations of Central populations are missing. For every population used in this study, a clearer explanation of what individuals were actually included in the data group is needed and the labeling should be consistent between the text and figures.

6) The paper fails to provide any actual statistical test to support the following statement. (Page 8, line 1-3, "Our analysis also revealed that numerous individuals from the IA group shared some fraction of their genetic ancestry with individuals from the MA group and contemporary East- Central European Slavic populations. In several cases, this fraction was significant"). An actual statistical test for significance is needed to support this.

7) It should also be noted that the admixture analysis which used 9 modern populations (Page 7, line 49-52) is a supervised one and a more specific description of why K=9 for reference populations is chosen is necessary.

**Authors’ response to reviewers**

Answer to the Reviewers
Reviewer #1
Below please find our answer to the criticism provided. The manuscript was changed according to the Reviewers suggestions. The modified or newly introduced fragments of the manuscript are highlighted in yellow.
Stolarek et al. present an extremely impressive dataset of ancient individuals from present-day Poland, dated to the Iron and Middle Ages, in order to study the genetic origin of the Slavs. They use an interesting indirect approach of ancestry determination by comparing the populations who lived in the region not directly before the appearance of the Slavs, but instead ~500 years before that, with Middle Ages Slavs. By analysing their genomes, they suggest that the Middle Ages Slavs were a "genetic continuation of the autochthon IA population". Unfortunately, I do not believe that the presented analyses support such conclusion of local continuity from the possibly admixed genomes of the IA individuals, and I am sadly disappointed that with such substantial amounts of data, no further analyses past PCA, ADMIXTURE, some few (poorly presented) f4-statistics, and uniparental haplogroups were done. In 2022 with such amounts of data, in my opinion, this is not to be expected for a journal such as Genome Biology, especially because all of the methods used require low amounts of data, and none provide statistically-supported quantitative results (e.g. ADMIXTURE vs qpAdm). The origin of the Slavs, like the authors say, is such an important and interesting subject, and so I really want to see this project succeed and the research on this population published. I hope the authors can critically accept my comments and improve them in a more in-depth, and more clear, study on these samples.
********************************************************************
Yes, the Reviewer has correctly recognized the intention of our work. We wanted to show, that the MA populations are genetic continuation of the autochthonous IA population. We also agree that f4 statistics measuring allele sharing with other ancient populations and qpAdm analyses are necessary to support our conclusions. We are very grateful to the Reviewer for his/her comments and ideas.
********************************************************************
Specifically, my major concern is that I did not find any presented convincing evidence that the IA and MA populations are related in any way, so any claim of continuity has no foundation in the data. Using similar ancestry proportions to infer continuity of populations is incorrect. The MA population could as easily correspond to a non-local population with similar ancestry profiles who migrated there. I think the authors should not rely on the heavy use of ADMIXTURE to support any definite claims of origin, especially when only modern populations are used as indirect proxies. This software is usually only used in ancient DNA research as a guideline for downstream analysis, because it can only produce qualitative results and does not consider population histories. Furthermore, the use of modern haplogroup data to infer past events cannot overcome conclusions based on existing ancient haplogroup data.
********************************************************************
We acknowledge the Reviewer requests and provided criticism. In accordance with the Reviewer suggestion, we extended the manuscript with the additional results section dedicated to f4 statistics of the studied IA and MA populations with respect to other ancient populations, and addressed the claims of genetic continuity of the local population since the Iron Age to medieval times through one-way and two-way modelling of the IA and MA populations. In this manner we specifically tested to which ancient populations the studied IA and MA populations are most closely related (f4 statistics). Regarding the question if the IA and MA populations are related we performed additionally one-way qpAdm modelling of the MA populations using the IA populations (separately for each site) as sources. We did show that each MA population can be modelled by at least one IA population, and often two or three of them produce viable qpAdm models (p>0.05).
Regarding the haplogroup data, yes we have reworded our manuscript so that it is clearly stated that we claim continuation of the uniparental lineages.
********************************************************************
More detailed concerns are in regards to the poor presentation of f4-statistics results (no f4 or Z value is present in the MS or SM or STables), which cannot be confirmed as to whether they produced any data supporting the authors' claims or not, and which from a purely visual interpretation of the figures, seem to suggest too small f4 values that might in fact not clearly support them.
********************************************************************

Yes, the figure might be misleading. We have provided the f4 statistics calculations in the Table S6 and redrawn the figure to represent on the X-axis the z-score values instead of f4-statistics values.
********************************************************************
One of my main suggestions is to re-analyse the data by looking at it into more detail, such as possibly identifying outlier individuals in IA and MA that could represent the local IA populations, and then using qpAdm modeling to see if a local model of continuity and admixture is the only valid result, or if the MA population could be modelled as other ancient populations (including as 1-way models).
********************************************************************
We agree that more in-depth analyses are needed to support our claims. We have devoted two new results sections for f4-statistics and qpAdm modelling. We attempted the genetic outliers identification according to the method described by Antonio et al. Science 2019 and Sirak et al. Nature Communications 2021, and described the procedure in the Supplementary Information. We found only one individual from the MA Markowice site to be a statistically significant outlier in the f4 statistics.
Provided below are specific f4 and qpAdm results answering the Reviewer question. Overall, we saw that the IA populations shared significantly more alleles with ancient northern Europeans (Denmark_IA and Norway_IA) than MA individuals. At the same time the IA populations shared less alleles with ancient Eastern Europeans (Latvia_BA). In one-way qpAdm the IA populations could be modelled as Denmark_IA (all three IA_Pruszcz_Gdanski, IA_Kowalewko and IA_Maslomecz) as well as by Ukraine_Scythians. Model competition showed, however, that Denmark_IA is better source of ancestry to IA populations than Ukraine_Scythians. In two-way models we showed that IA populations can be modelled as preceding Poland_EBA_Unetice + Denmark_IA. Majority of the IA populations ancestry was assigned to the Denmark_IA. We also observed that Hungary_Longobard + one of multiple MA populations can explain all three IA populations, where majority of IA population ancestries are assigned to a given MA population.
Importantly, we found the two-way models of IA populations that included MA as a source of ancestry outcompeted the two-way models of IA populations with Poland_EBA_Unetice as a source of the local ancestry.
Further to test for genetic continuity between IA and MA periods we show that MA populations can be modelled in one-way qpAdm by at least two IA populations, and often by all three.
Finally, we did not find any biologically plausible qpAdm two-way model which would explain MA populations and not IA populations.
Two-way MA population models using IA populations as one source of ancestry and as a second local population proxies: Poland_EBA_Unetice, Czech_EBA_Unetice or Lithuania_BA, showed that the majority of the ancestry of the MA populations was assigned to the IA populations. The model which fitted all MA populations was: Poland_EBA_Unetice + IA_Maslomecz.
********************************************************************
My detailed comments are below. Note that line numbers mentioned are approximate, as they don't properly align with the text in the manuscript.
Abstract, Line 10: To help the reader, I think it would help to specify that these immigrant IA populations belonged to the Wielbark culture.
********************************************************************
We have revised the manuscript accordingly.
********************************************************************
Results, Studied group description: There is no information regarding terminal deamination in Table S1. Also, are the numbers of nuDNA reads total reads of unique aligned reads to the human genome? And although the authors mention that they "performed a whole-genome analysis of representatives of the IA Wielbark and MA Slav cultures.", in Table S1 there are plenty of IA individuals that are not attributed to the Wielbark culture, even though the manuscript says all sites are Wielbark. This needs to be clarified. Lastly, the authors mention that 197 samples produced genome-wide data, but on Table S1 only 135 seem to show that.
********************************************************************
We have revised the Table S1. We added a column with terminal deamination rates for each individual. The nuDNA reads are total uniquely aligned reads with MAPQ of at least 20 to the human reference genome version GRCh37. With regards to the archaeological culture assignment this was an oversight on our part, which unfortunately turned out to be very confusing. Only two individuals from the IA Gaski site (PCA0011 and PCA0012) were associated with the Przeworsk culture. All other IA individuals presented in this work were associated with the Wielbark culture. We revised the table accordingly. We have revised the sample numbering. It should be, however, mentioned that not all analyses involved the same number of samples.
********************************************************************
Could it be that the IA individuals studied are not only from the Wielbark culture, but also from others (likely local)? If this is the case, it would really help in the interpretation and presentation of the results to always specify which IA population is being discussed. Or, is it even possible to separate the IA and MA individuals into different groups (for example using qpWave?), that could then be used independently for further analyses? Based on the ADMIXTURE plot in 1D it seems like the populations are fairly variable (although the very-low coverage of some individuals could also play an important role there), and maybe some outliers are not Wielbark, but instead locals (maybe mostly females?).
********************************************************************
As we have already mentioned, only two individuals from the IA Gaski site (PCA0011 and PCA0012) were associated with the Przeworsk Culture. All other IA individuals presented in this work were associated with the Wielbark culture.
We attempted the subdivision of the IA populations with qpWave into male and female subgroups. No statistical significance was reached when subdividing into male and female subgroups.
Further we attempted to subdivide IA group into distinct genetic subpopulations. We haven’t identified any statistically significant genetic outliers against several plausible sources of local ancestry (including Lithuania_BA and MA individuals). Given this result we performed one-way qpAdm models of each IA site by using as ancestry source other IA site. We found that subdivision down to individual sites is justified as all three Wielbark sites (IA_Pruszcz_Gdanski, IA_Kowalewko and IA_Maslomecz ) are not one-way mixtures of each other, specifically IA_Kowalewko is not a valid one-way model from IA_Pruszcz_Gdanski.
********************************************************************
Results, Studied group description page, line 25: Please specify the conditions to qualify data as "suitable for further analyses". Is it just the 10.000 SNPs threshold mentioned in the Methods section?
********************************************************************
Yes, at this point after removing related and contaminated individuals we followed through with the PCA/ADMIXTURE/f4 and qpAdm with samples that had at least 10.000 SNPs. Nonetheless specific preparations to additional analysis (like LD pruning) further changed number of samples in each analysis. Where feasible we always provided the number of individuals used in the analysis (eg. qpAdm).
********************************************************************
Results, Studied group description page, line 32: Table S1 seems to be missing this information. Also, please clarify what the purple colored (at least in my downloaded version of the document) columns labeled as "mt / Whole Genome Enrichment Method / Y" correspond to. Are they all about enrichment?
********************************************************************
We have corrected the manuscript. The information is presented in the Tables S3 and S4. We modified the Table S1 to clearly state that the markings in these columns relate only the information if a given sample was enriched with myBaits probes for mt/WGE or Y SNPs.
********************************************************************
Results, IA- and MA-group affinities page 1, lines 36-40: As there are more than one analysis dataset, please mention which one was used (HO, POPRES or ancestry-restricted).
********************************************************************
We have corrected the manuscript accordingly.
********************************************************************
Results, IA- and MA-group affinities page 1, line 49: Please present the other ADMIXTURE plots for the remaining K values in the SM.
********************************************************************
We reproduced the Figure S17 as requested.
********************************************************************
Results, IA- and MA-group affinities page 2, lines 1-3: If these results are based on ADMIXTURE, the authors need to reword these sentences. None of the 9 admixture components presented represents the MA group, therefore it cannot be said that another group has a fraction of another who is not one of the defined ancestries. They might share similar structure or ancestry profiles, which does not mean that they are closely related. Also, the word "significant" must be restricted to statistically-backed results, which is not the case for ADMIXTURE and PCA analyses.
********************************************************************
Yes, the unsupervised ADMIXTURE is not a direct test of ‘genetic relationships’ between populations. We have corrected the manuscript accordingly.
********************************************************************
Results, IA- and MA-group affinities page 2, line 12: Please mention which dataset was used (HO, POPRES or ancestry-restricted).
********************************************************************
We have corrected the manuscript accordingly.
********************************************************************
Results, IA- and MA-group affinities page 2, line 33: The mentioned f4 statistic is not the same shown in Fig. 2c, please correct the required one. Also, please mention f4 values and Z-scores, and whether the results being discussed were statistically significant. I was not able to find the value results of these f4-statistics (plus Z-scores and SNP numbers), and nowhere in the manuscript or SM is there a mention of the used threshold of significance for drawing conclusions. By looking at Fig S21, Fig. 2c, and the Methods, there is no mention of significance, and there is no mention of what the bars represent. Even if 2SE, many tests overlap with the 0 line, many others have the error bars close to it, and overall the presented range of values of f4 (-0.00075 to 0.00050) suggests that only a handful of the tests might be statistically significant, if any at all.
********************************************************************
This is true the Figure 2c might be misleading, we have reproduced it with Z-scores on X axis.
As we have observed mainly trends and not statistically significant f4-statistics results for the f4(Mbuti, X; Western Europeans, Norwegians) we have removed this analysis from the manuscript.
********************************************************************
Results, IA- and MA-group affinities page 2, lines 44-48: Not completely necessary, but it would be great to have the SNPs in Table S2 organised and identified by the phenotypical characteristic they relate to.
********************************************************************
We have revised Table S2 as requested.
********************************************************************
Results, IA- and MA-group affinities page 2, last paragraph: I am unfortunately not convinced of the "locality" of these claims due to 2 points, and that is my main critique of this manuscript:
1)the ancestries of the IA and MA populations based on PCA and ADMIXTURE do seem to be different but it is not proved with any analyses that there is any kind of IA-MA continuity or relationship. Of course there is no local IA data to more directly test that, but the authors could have at least tried to use local Bronze Age individuals. Also, continuity is a hard concept to prove in paleogenomics, but analyses such as qpWave and competition-based qpAdm with other ancient populations might help understand these processes.
********************************************************************
For a more direct test of genetic continuity we have performed one-way qpAdm modelling of the MA populations (divided into separate sites) from the IA populations (divided into separate sites). We found that all MA populations formed viable one-way models with at least one IA population, and most usually with two or all three of them (IA_Pruszcz_Gdanski, IA_Kowalewko or IA_Maslomecz).
To further show genetic continuity in this region we calculated two-way models of IA_populations and MA_populations under the same set of right outgroup populations. As proxies for local autochtones we used Bronze Age populations including: Poland_EBA_Unetice, Czech_EBA_Unetice and Lithuania_BA. In case of the IA_populations we tested if two-way models involving local autochtone proxies and ancient northern populations (Denmark_IA, Norway_IA) do model our IA populations. We found that indeed all three IA populations (IA_Pruszcz_Gdanski, IA_Kowalewko or IA_Maslomecz) formed viable two-way models.
As more direct test for autochthon continuity we modelled IA populations as the Reviewer suggested by testing two-way qpAdm models of Hungary_Longobards and MA populations. All IA populations could be modelled in this way with several MA populations fitting the two-way models with Hungary_Longobards for all three IA populations (IA_Pruszcz_Gdanski, IA_Kowalewko, IA_Maslomecz). Importantly, the IA populations could not be modelled as Hungary_Longobards + Poland_EBA_Unetice or Czech_EBA_Unetice.
Two-way MA population models using the IA populations as one source of ancestry and as a second local population proxies: Poland_EBA_Unetice, Czech_EBA_Unetice or Lithuania_BA, showed that the majority of the ancestry of the MA populations was assigned to the IA populations. The model which fitted all MA populations was: Poland_EBA_Unetice + IA_Maslomecz.
********************************************************************
2)The MA affinities to modern East-Central populations could equally represent a MA group arriving from a different region, admixing, and eventually establishing themselves in the region so that modern populations share similarities with them. No reasoning is presented to support the belief that they represent the local IA population instead of another eastern group.
********************************************************************
As provided in the explanations above, we observed that the MA populations share more alleles with ancient east European population than the IA populations. At the same time we found multiple two-way qpAdm models showing, that the MA populations derive majority of their ancestry from the IA populations and only a small fraction from the preceding BA populations.
We also showed that all IA populations can be modelled as mixture of Hungary_Langobard and multiple MA populations, where majority of the ancestry is assigned to come from the MA populations. In these analyses when the MA populations are swapped for the preceding BA populations the models are no longer valid.
Further we showed that each MA populations can be modelled with one-way qpAdm by using several of the IA populations as ancestry sources. In these analyses IA_Maslomecz formed valid one-way models with all MA populations.
********************************************************************
One suggestion I have is that the authors could use qpAdm to test whether Wielbark can be modelled as MA plus for example Netherlands BA or IA (Patterson et al. 2022), or also Hungary Longobards who they seem to suggest are similar to the population who migrated North-to-South through Poland, but for which admixture was not thoroughly tested. That seems to be the most straightforward test to see if MA would represent the local population who mixed with the incoming Longobards to represent the Wielbark.
********************************************************************
Yes, we did model the IA populations as the reviewer suggested by testing two-way qpAdm models of Hungary_Longobards and MA populations. All IA populations could be modelled in this way with several MA populations fitting the way-models with Hungary_Longobards for all three IA populations (IA_Pruszcz_Gdanski, IA_Kowalewko, IA_Maslomecz). Importantly the IA populations could not be modelled as Hungary_Longobards + Poland_EBA_Unetice or Czech_EBA_Unetice.
********************************************************************
As I mention in another comment, it could be interesting to try to identify different clusters of individuals within the IA (and MA), as some of those might be more direct representatives of the local IA population. Perhaps some of those local individuals moved over to live with the Wielbark groups, and was therefore inhumated instead of cremated, giving you direct access to local IA individuals. This would only be an hypothetical situation, but if indeed outliers are detected, it can be argued (based on the MT and Y data you have) that they could be locals.
********************************************************************
We tested each IA individual for being an outlier. f4 statistics testing if given individual is more closely related to MA, Poland_EBA_Unetice, Czech_EBA_unetice or Lithuania_BA individuals than to the rest of the individuals from the IA group or selected site. We were not able to find any outlying individuals with statistical significance even at z-score >3 (which is already a pretty low bar to pass for being counted as outlier).
********************************************************************
Uniparental marker analysis, overall: It would useful to compare the IA data with existing ancient Polish individuals before the Iron Age.
********************************************************************
In Discussion we have extended the text with appropriate comparisons to previous populations from the region.
********************************************************************
Uniparental marker analysis, page 1, lines 17-25: It seems to be a substantial leap to use as evidence for continuity, locality, and admixture, the presence of Y-hg R1a in large frequencies in MA but with some small cases in the IA. Haplogroup Y-hg R1a was already present in the Poland in the Late Neolithic and BA as well (see Olalde et al. 2018; Fernandes et al. 2018; Malmstrom et al. 2019), although at small frequencies (3/17 Chalcolithic and Bronze males). How do the authors explain such an explosion to eventually represent 57.5% of all Y-hg in the MA? Some local pressure is one possibility, but a migration of external populations is another, and perhaps more likely. R1a is also found in IA Czechia, Slovakia, Estonia, Mongolia, and it is the major Y-hg found in the Srubnaya (12/14 males) and Sintashta (23/31 males) cultures. Sample bias is also a possibility, but in the end I don't believe this can be used to inform locality and continuity of the MA group.
********************************************************************
Yes, the increase in frequency of R1a ‘out of nowhere’ is more compliant with the migration scenario. However, we do note that the dominant burial practice in this part of Europe since 1300 BC was cremation rather than inhumation. After Unetice culture (closest in time to our IA populations) throughout of the Bronze Age central-east Europe witnessed a number of archaeological cultures including Lusatian, Pomeranian and Przeworsk cultures in all of which cremation was a dominant burial practice.
Regarding the frequencies of Y-hgs. Please note the work of (Dynamic changes in genomic and social structures in third millennium BCE central Europe | Science Advances) and Y-hg frequencies of Globular Amaphora associated people. Corded Ware people were mainly R1a-M417, and Globular Amphora was characterized by high frequency of I2a. Bell Beaker were primarily R1b, whereas Unetice Culture was a pretty even mix of R1b/R1a-Z645 and I. We do see a very consistent continuation of I2a lineages in IA and MA populations. When we look at the Y-hg frequencies as what goes away first we see that when highly prevalent I/I1 leaves it also makes a lot of space for already existing in high frequencies R1a-M417 from Corded Ware people. Resurgence of local populations in Central Europe was described on several occasions.
Considering the observed differences in the patterns of Y-hg frequencies at the beginning and end of the first millennium CE as well as the stability of the mt-hg frequency profiles are products of androcentric and paternalistic social structures, characteristic to this period. Usually the men migrated and the women were local. This phenomenon may also explain the low incidence of Y-hg R1a in the IA group. There are many examples in history showing that a relatively small group of male immigrants can subjugate a local community (e.g. the history of the colonization of North and South America). Thus, a small number of individuals with Y-hg R1a in the IA group does not necessarily mean a low frequency of this haplogroup in the autochthonous IA population. It may, however, indicate a natural phenomenon of excluding local men from communities dominated by male immigrants.
In our manuscript we provide several pieces of evidence that the ancestors of the medieval populations lived in the region of present-day Poland during the IA. There are, however several aspects that need further elucidation. Firstly, how and when the ancestors of the MA populations with Y-hg R1a appeared. The times when Y-hg R1a-M417 dominated in this territory are associated with the spread of the Corded Ware culture (from 3000 to 2300 BC). Later it was replaced by the Unetice culture (from 2300 to 1600 BC) that was associated with the populations in which Y-hg R1a was very rare. From then until the IA, there were many archaeological cultures in this region from which no genetic data is available as cremation became the dominant burial practice. Here, we showed that the IA and MA populations inherited only a small percentage of genetic ancestry from the people associated with the Unetice culture. Therefore, the ancestors of the autochthonous IA populations with Y-hg R1a would have either had to be revived in the BA period or come from east during the BA or IA period. At this point, it should be noted that based on our results, one cannot explicitly rule out additional waves of migration after the IA. Thus, one of the reasons for the increase in the frequency of Y-hg R1a could also be migrations from Eastern Europe after the Migration Period. In this case, the MA populations would not necessarily be direct descendants of the autochthonous IA population.
********************************************************************
Uniparental marker analysis, page 1, lines 26-30: Y-hg R1b has been present in Poland before the IA and MA. During the Beaker period and BA it is the overwhelming Y-hg (Linderholm et al. 2020), so it cannot be associated specifically with Northwestern European populations and a migration from the West. If the authors are referring to a very specific sub-clade, they should mention it like they did for R1a.
********************************************************************
We have modified the manuscript accordingly and do not name R1b as ‘Northwestern European’.
********************************************************************
Discussion, page 1, line 25: I don't think the author presented any evidence of admixture with local populations using the genome-wide data. ADMIXTURE plots with similar ancestries to other populations cannot be understood as evidence of admixture with those populations. Those ancestry profiles could, for example, be present in some of the individuals who migrated from the Northwest with the Wielbark. They can also represent admixture with other populations outside of the territory analysed. Above I suggested possible analyses to look into this.
********************************************************************
Accordingly, we have performed the suggested analyses and found close genetic relationships between the IA populations and the MA populations as well we were able to model the IA populations as two-way mixtures between ancient IA northern populations and local proxies (Poland_EBA_Unetice, Czech_EBA_Unetice, Lithuania_BA as well as local the MA populations). Also, models involving Hungary_Langobards and local MA populations produced viable mixes forming the IA populations.
********************************************************************
Discussion, page 1, lines 27-30: Please clarify this sentence. As it currently is, it suggests that the admixture seen in the Wielbark genomes is the same that existed in the MA group and in the Western Slavs. One of your main findings was that IA and MA had substantially different admixture profiles, so here it sounds like you are saying that instead they were the same. Also, Western Slav populations are mentioned in here for the first time, without any context during the presentation of the results, so it is not clear who they refer to.
********************************************************************
We have clarified our wording in the manuscript. We were pointing out to the local population which persisted through the IA migrations and resurged during the MA.
********************************************************************
Discussion, page 1, line 33: What do you mean by "contemporary"? Which other Medieval East-Central European ancient populations does this refere to? From Fig. S16, no other analysed ancient population has a proportion distribution of Eastern ancestry even close to that of the MA group.
********************************************************************
We were referring to present-day East-Central Europeans. We have corrected the manuscript accordingly.
********************************************************************
Discussion, page 1, lines 48-59: Again, the idea that this high R1a frequency appeared in the MA cannot be discarded as having had an origin outside of this region. The authors are comparing their ancient MA data with modern populations, but the other way around does not provide in any way evidence of origin in the past. If anything, it is evidence of local continuity of haplogroup distributions to modern times. One can more easily go forward in time with these speculations, but not back.
********************************************************************
Yes, we have reworded this part. Indeed moving backwards in time we are finding a point where R1a frequencies where high in Central-East Europe. Please note the interpretation we provided above.
********************************************************************
Methods: Were reads trimmed at the terminal ends, to exclude deaminated positions from downstream analysis (especially for hg determination)? If not, were the libraries selected for deeper sequencing UDG treated?
********************************************************************
Yes, after establishing the terminal deamination measures with mapDamage 2.0, the reads were realigned to the genome with trimming last 5 bases.
********************************************************************


 
Reviewer #2:
Below please find our answer to the criticism provided. The manuscript was changed according to the Reviewers suggestions. The modified or newly introduced fragments of the manuscript are highlighted in yellow.
This paper by Stolarek et al. attempts to use ancient DNA data to investigate when historical Slavs first entered the territory of present-day Poland. It uses autosomal SNPs, mtDNA and Y chromosomes as genetic markers, comparing a large sample of Iron Age (IA) and Middle Age (MA) individuals to modern European populations using PCA and model-based clustering analysis. Most notably their results reveal that IA individuals show more similarity to modern northwestern European populations while MA individuals more closely resemble modern eastern European populations.

The primary conclusion of this paper is 1) there is genetic continuity between IA and MA groups in present-day Poland and 2) there were two migrations from Northern Europe and Western Europe to present-day Poland in the first millennium.
********************************************************************
We have reworded the manuscript. We maintained the claim that there was a migration from Northern Europe in the early first millennium AD. These migrants admixed with the locals and show genetic continuity with the later MA populations. We showed that 6th CE East to West migration was not necessary for forming MA gene pool. Nonetheless it cannot be excluded given the significant change in Y-hg frequencies. However we see a stable pool of matrilineal lineages from IA to MA which indicated mostly male migrations.
For the claims about second migration from Western Europe to the region of present-day Poland we have observed mainly trends and not statistically significant f4-statistics results for the f4(Mbuti, X; Western Europeans, Norwegians) therefore we have removed this analysis from the manuscript.
********************************************************************
The paper provides a large number of ancient genomes (almost 500 genomes total, almost 200 of which have sufficient endogenous DNA and sequencing data for downstream analysis) from early and later Medieval eastern Europe, a region and period for which there is a paucity of existing published data. Generally the population genetic methods applied in the paper are done in an appropriate way (PCA, model-based clustering, FST and drift analysis [F4]); however, how the authors interpret the results of these analyses to form their conclusions is problematic. The conclusions the authors draw cannot be robustly inferred based on current evidence, and some important issues, particularly with regard to a) describing the generation of the data and b) nomenclature used to describe the affiliation of individual genomes, need to be resolved for this paper to be suitable for publication.


Major issues
1) The results of this paper do not support the two main conclusions.

1a) (Page 11, line 26-28, "(i) the population inhabiting present-day Poland in the MA was genetic continuation of the autochthon population living at the same territory in the IA". This appears to be a misunderstanding of what the Admixture results represent. Sharing same genetic components given by Admixture analysis using external reference populations doesn't necessarily mean the two groups actually share same genetic ancestry indicative of population continuity (Page 8, line 1-3, "numerous individuals from the IA group shared some fraction of their genetic ancestry with individuals from the MA group and contemporary East- Central European Slavic populations".) since clustering analysis is highly dependent on what references are used. It is necessary to provide a formal test (e.g. qpAdm, qpWave, Some kind of explicit model-testing framework like Approximate Bayesian Computation etc) that can clearly suggest there is sharing of genetic ancestry from one period to the next, as well as determine the degree of continuity.
********************************************************************
We have devoted two new results sections for f4-statistics and qpAdm modelling.
For a more direct test of genetic continuity we have performed one-way qpAdm modelling of the MA populations (divided into separate sites) from IA populations (divided into separate sites). We found that all MA populations formed viable one-way models with at least one IA population, and most usually with two or all three of them (IA_Pruszcz_Gdanski, IA_Kowalewko or IA_Maslomecz).
To further show genetic continuity in this region we calculated two-way models of IA_populations and MA_populations under the same set of right outgroup populations. As proxies for local autochthones we used Bronze Age populations including: Poland_EBA_Unetice, Czech_EBA_Unetice and Lithuania_BA. In case of the IA populations we tested if two-way models involving local autochthone proxies and ancient northern populations (Denmark_IA, Norway_IA) do model our IA populations. We found that indeed all three IA populations (IA_Pruszcz_Gdanski, IA_Kowalewko or IA_Maslomecz) formed viable two-way models.
As more direct test for autochthon continuity we modelled IA populations as one of the Reviewers suggested by testing two-way qpAdm models of Hungary_Longobards and MA populations. All IA populations could be modelled in this way with several MA populations fitting the two-way models with Hungary_Longobards for all three IA populations (IA_Pruszcz_Gdanski, IA_Kowalewko, IA_Maslomecz). Importantly IA populations could not be modelled as Hungary_Longobards + Poland_EBA_Unetice or Czech_EBA_Unetice.
Two-way MA population models using IA populations as one source of ancestry and as a second local population proxies: Poland_EBA_Unetice, Czech_EBA_Unetice or Lithuania_BA, showed that the majority of the ancestry of the MA populations was assigned to the IA populations. The model which fitted all MA populations was: Poland_EBA_Unetice + IA_Maslomecz.
********************************************************************
1b) (Page 11, line 28-32, "(ii)during the first millennium the genetic makeup of East-Central European autochthones was affected by two major demographic events, the migrations of Northern Europeans in the 1st - 4th century and Western Europeans in 10th - 12th century"). There are a number of issues with this claim:
(i)This study only uses modern reference populations to orientate the past populations of interest and it is unknown if modern populations can reflect the contemporary genetic makeups of ancient individuals in the same geographic regions. For instance, the similarities between IA groups and modern northwestern European populations does not mean the IA groups have genetic similarities with ancient northwest populations. Comparison between contemporary groups are necessary to address the migration hypothesis. To obtain contemporary northern and western individuals, the authors can refer to (Margaryan et al. 2020; Gretzinger et al. 2022).
********************************************************************
Yes, we have reanalysed all data with respect to preceding and contemporary ancient populations with several f4 statistics and qpAdm one-way and two-way models.
Overall, we saw that the IA populations shared significantly more alleles with ancient northern Europeans (Denmark_IA and Norway_IA) than MA individuals. At the same time the IA populations shared less alleles with ancient Eastern Europeans (Latvia_BA). In one-way qpAdm the IA populations could be modelled as Denmark_IA (all three IA_Pruszcz_Gdanski, IA_Kowalewko and IA_Maslomecz) as well as by Ukraine_Scythians. Model competition showed, however, that Denmark_IA is better source of ancestry to the IA populations than Ukraine_Scythians. In two-way models we showed that the IA populations can be modelled as preceding Poland_EBA_Unetice + Denmark_IA. Majority of the IA populations ancestry was assigned to the Denmark_IA. We also observed that Hungary_Longobard + MA populations can explain all three IA populations, where majority of the IA populations ancestries are assigned to the MA populations. At the same time we we tried to model IA populations as Hungary_Longobards + preceding putative local populations as ancestry sources (eg. Poland_EBA_Unetice) these models were no longer valid.
Further to test for genetic continuity between the IA and MA periods we show that the MA populations can be modelled in one-way qpAdm by at least two IA populations, and often by all three.
Finally, we did not find any biologically plausible qpAdm two-way model which would explain the MA populations and not the IA populations.
Two-way MA population models using the IA populations as one source of ancestry and as a second local population proxies: Poland_EBA_Unetice, Czech_EBA_Unetice or Lithuania_BA, showed that the majority of the ancestry of the MA populations was assigned to the IA populations. The model which fitted all MA populations was: Poland_EBA_Unetice + IA_Maslomecz.
********************************************************************
(ii) Even if the paper demonstrates there are genetic similarities between IA groups and contemporary northwest populations, this does not mean there was necessarily a migration into modern-day Poland during this period. The paper didn't not analyze any ancient individuals excavated in Poland that date prior to IA groups so there is no way to determine if there was actually a regional genetic change (which might have been contributed by the immigrants) within the IA periods. It is quite possible that the previous population looked much like the IA population, even if they both looked somewhat northern European in ancestry (due to a potentially much earlier migration, in either direction). Similarly, there is a large time gap between the IA and MA individuals (around 600 years), which is certainly long enough for many different demographic changes, and thus difference in genetic makeup between IA and MA individuals cannot be used as evidence to support any particular migration event (the shift in ancestry could have involved multiple pulses, undetected ancestry shifts or simply a long-term gradual change). In order to test the two hypothese, it is necessary to provide genetic data from Poland prior to the IA as well as between IA and MA. If it is impossible to get aDNA prior to the IA, strontium isotope analysis from the IA individuals might help to uncover if there was migration.
********************************************************************
We have extended the analyses by incorporating the necessary ancient populations for f4 statistics and qpAdm modelling. Specifically to test the IA migration hypothesis we have shown with f4(IA_X, MA, Test population, Yoruba), that significantly more alleles were shared with ancient northern European populations (Denmark_IA, Norway_IA) by the IA_X populations compared with the MA group. Further we showed that one-way qpAdm model of the IA populations (Pruszcz_Gdanski, Kowalewko, Maslomecz) is always achieved when Denmark_IA is a source population, whereas preceding ancient populations do not produce valid one-way models for any IA population.
We found multiple two-way qpAdm models showing, that MA populations derive majority of their ancestry from the IA populations and only a small fraction from the preceding BA populations.
We also showed that all IA populations can be modelled as mixture of Hungary_Langobard and multiple MA populations, where majority of the ancestry is assigned to come from the MA populations. In these analyses when the MA populations are swapped for the preceding BA populations the models are no longer valid.
Further we showed that each MA populations can be modelled with one-way qpAdm by using several of the IA populations as ancestry sources. In these analyses IA_Maslomecz formed valid one-way models with all MA populations.
********************************************************************
2) Specific criteria are needed to illustrate why some ancient individuals were called Slavs (Page 6, line 9) or Longobards (Page 10, line 30). Whether this determination was based on historical records or archaeological artifacts should be mentioned. Even if the criteria is provided, it is also highly problematic to assign ancient individuals to specific ethnic groups since we never actually know how people recognize themselves in the past. Identity is complex and it is insufficient to illustrate it using a single word. We have no idea (and will never know) if the people in this paper actually identified as "Slavs" or "Longobards". In this case, using time and space (geography) to describe ancient groups would be a much less problematic approach.
********************************************************************
We referred to these populations when there is clear archaeological and/or historical nomenclature for the archaeological sites from which these individuals were excavated. We have reworded the manuscript accordingly not to name the studied populations by their potential (but unknown) ethnicity. We note however that when we use name Hungary_Langobard it is difficult to change this name as it is directly provided by the AADR resource and is used throughout the aDNA publications.
********************************************************************
3) For the third PCA ancestry-restricted dataset (Page 12, line 29), it is hard to understand why specific language groups (Gaelic, Germanic, Slavic, Baltic and Norse) were used and who were assigned to these groups (are the assignments based on country)? The purpose of this PCA analysis should be addressed. Meanwhile, the terms are not really comparable since Gaelic and Baltic are much smaller groups compared with the Germanic and Slavic groups. Similarly, in the figure 2a, it is not reasonable to put language groups (Baltic, Finnish and Norse) and geographical groups (North-western Europeans and Eastern Europeans) together as references. These groups are not comparable.
********************************************************************
We changed the names of the population. We believe that now they will not be confused with language groups. The intention from the start was to name the populations by geography. We also described in the figure caption which populations are under each label.
********************************************************************
Minor issues

4) (Page 11, "Ancient DNA analysis"). A clearer and comprehensive description of how samples were processed (e.g. How the aDNA were enriched for different autosomal and sex-specific markers) and analyzed is needed. It would be very hard to replicate the methods for data generation as described.
********************************************************************
We have added a more specific description where this information is placed in Supplementary Information (Supplementary Note 4).
********************************************************************
5) The labeling of this paper is not consistent. For instance, "East-Central" populations are mentioned (e.g. Page 8, line 2-3, Page 8, 14-15) several times, but in the legend of admixture plots there are only "Eastern populations". The annotations of Central populations are missing. For every population used in this study, a clearer explanation of what individuals were actually included in the data group is needed and the labeling should be consistent between the text and figures.
********************************************************************
We have corrected the manuscript and figures as requested.
********************************************************************
6) The paper fails to provide any actual statistical test to support the following statement. (Page 8, line 1-3, "Our analysis also revealed that numerous individuals from the IA group shared some fraction of their genetic ancestry with individuals from the MA group and contemporary East- Central European Slavic populations. In several cases, this fraction was significant"). An actual statistical test for significance is needed to support this.
********************************************************************
Yes, this was an overinterpretation of the ADMIXTURE results. The two populations have similar ancestry profiles. To directly test for the genetic relationships between these two groups we performed one-way and two-way qpAdm modelling.
As described above we found multiple two-way qpAdm models showing, that the MA populations derive majority of their ancestry from the IA populations and only a small fraction from the preceding BA populations.
We also showed that all IA populations can be modelled as mixture of Hungary_Langobard and multiple MA populations, where majority of the ancestry is assigned to come from the MA populations. In these analyses when MA populations are swapped for the preceding BA populations the models are no longer valid.
Further we showed that each MA populations can be modelled with one-way qpAdm bu using several of the IA populations as ancestry sources. In these analyses IA_Maslomecz formed valid one-way models with all MA populations.
********************************************************************
7) It should also be noted that the admixture analysis which used 9 modern populations (Page 7, line 49-52) is a supervised one and a more specific description of why K=9 for reference populations is chosen is necessary.
********************************************************************
This is an unsupervised admixture. The K=9 was chosen based on lowest cross-validation error, where we tested the K’s 2 through 10. We corrected the methods and supplementary information accordingly.

**Second round of review**

**Reviewer 1**

-

**Reviewer 2**

The revised manuscript incorporates a substantial amount of new analysis, primarily using f4 statistics and qpAdm. It successfully addresses many of the previously raised questions, including the major concerns regarding the genetic continuity between the IA and MA groups, the nomenclature of past populations, as well as minor issues. However, regarding major issue 1b, it remains unclear which evidence can substantiate the conclusion that "there was a migration from Northern Europe in the early first millennium AD."

Regarding the section on the "Genetic history of the IA group," the qpAdm analysis suggests that the northern IA group could serve as a source of ancestry (in either one-way or two-way models) for the IA_X groups. However, it does not necessarily imply that this migration occurred specifically during the first millennium AD. Based on the available evidence, it is also plausible that the migration took place between the BA and the first millennium AD. Therefore, while proposing the migration from northern Europe as a hypothesis is appropriate, the current evidence falls short of supporting it as a conclusive statement. Therefore the authors should temper down their emphasis on this migration, and raise it more speculatively.

There are also some minor issues raised in the revised manuscript:


Results, page 4, line 27-29: The criteria used by the author to select suitable individuals for further analyses remain unclear.

Results, page 4, line 52: The ADMIXTURE analysis employed in this study was supervised.

Results, page 4, line 60: Figure S21 was not found in the manuscript.

Results, page 5, line 3: In Figure S15, the ranges of Fst values differ between the two plots, causing confusion. It is recommended that the author utilize consistent standards for both figures.

Results, page 5, line 3-7: The statement that IA is similar to MA appears contradictory, as IA is actually more ancient than MA. The paragraph should exemplify the aspects in which they were similar, providing specific examples of individuals that exhibited similarity. Additionally, the author mentioned that the similarity was substantial but failed to provide any quantitative analysis.

Results, page 5, line 9: The resolutions of Figures S16-19 are too low and should be improved.

Results, page 5, line 48: It is difficult to extract specific phenotypic predictions from Table S2. It would be helpful if the author could include additional columns displaying the probabilities of specific phenotypes, such as blond hair.

Results, page 5, line 53: Figure S19 does not correspond to the description provided by the authors. Should it be Figure S20A instead?

Results, page 8, line 7-18: It is unclear why the author modeled the ancestry of an earlier group (IA_X) using a later group (MA_X). Further clarification is needed.

Discussion, page 9, line 47: There is no evidence indicating that the northern immigrants mixed with the autochthonous IA populations. Although the paper listed five populations that could approximate the local IA group, the use of qpAdm analysis to model the genetic ancestry of IA_X using northern IA populations and these five "local" IA populations may not be supported, as there might not have been a genetically differentiated local IA group in this region prior to the IA individuals sampled in this study.
Regarding the response to reviewer comment 2) on nomenclature, the author’s respond: “We note however that when we use name Hungary_Langobard it is difficult to change this name as it is directly provided by the AADR resource and is used throughout the aDNA publications.”. This is insufficient as an explanation The original Amorim manuscript never refer to the individuals as Lang/Longobards, but instead emphasizes: “whether the association between genetic ancestry and material culture reflects specific peoples mentioned in historical texts (i.e., Longobards) or stemmed from a deeper/long-term descent (of mixed barbarian ancestries) is as yet unclear.” We would refer the authors to Eisenmann et al. Scientific Reports volume 8, Article number: 13003 (2018) for more discussion on this matter.

**Authors’ response to reviewers**

Answer to Reviewer 2

Reviewer reports:

Reviewer #2: The revised manuscript incorporates a substantial amount of new analysis, primarily using f4 statistics and qpAdm. It successfully addresses many of the previously raised questions, including the major concerns regarding the genetic continuity between the IA and MA groups, the nomenclature of past populations, as well as minor issues. However, regarding major issue 1b, it remains unclear which evidence can substantiate the conclusion that "there was a migration from Northern Europe in the early first millennium AD."

Regarding the section on the "Genetic history of the IA group," the qpAdm analysis suggests that the northern IA group could serve as a source of ancestry (in either one-way or two-way models) for the IA_X groups. However, it does not necessarily imply that this migration occurred specifically during the first millennium AD. Based on the available evidence, it is also plausible that the migration took place between the BA and the first millennium AD. Therefore, while proposing the migration from northern Europe as a hypothesis is appropriate, the current evidence falls short of supporting it as a conclusive statement. Therefore the authors should temper down their emphasis on this migration, and raise it more speculatively.

Yes, in essence we do agree with the Reviewer that the results presented in our manuscript do not allow to determine precisely when did the migration from northern Europe happen. However, our data in combination with the former archaeological findings point at the beginning of the first millennium as the most likely moment when the migration could have started. Considering the above fact, we have tempered our conclusions.
##############################################################

There are also some minor issues raised in the revised manuscript:


Results, page 4, line 27-29: The criteria used by the author to select suitable individuals for further analyses remain unclear.
We have specified more clearly the criteria used to select particular individuals for further analyses: PCA, ADMIXTURE and qpADM.

##############################################################

Results, page 4, line 52: The ADMIXTURE analysis employed in this study was supervised.
In general we agree with the Reviewer, however, in this case it is not a standard supervised/unsupervised clustering but an option in the ADMIXTURE software. Running this analysis in the supervised mode will not reproduce the result presented in Fig. 1d.

##############################################################
Results, page 4, line 60: Figure S21 was not found in the manuscript.
We have corrected the manuscript accordingly. The missing figure is numbered as S19.

##############################################################
Results, page 5, line 3: In Figure S15, the ranges of Fst values differ between the two plots, causing confusion. It is recommended that the author utilize consistent standards for both figures.
Yes we agree that it is usually a good practice to have a common scale on plots. However, please note that in our case the left hand side of the plot has a Fst scale from 0.092 to 0.113, whereas right hand site scale goes from 0.213 to 0.222. Both scales don’t overlap, which when making a common scale will cause that the left hand site will be in shades of blue, whereas the right hand side in shades of red. Therefore we propose to leave the plot as it is, where it serves as a relative visualization of Fst values for each studied group. An alternative would also be to split the figure into two separate ones.

##############################################################
Results, page 5, line 3-7: The statement that IA is similar to MA appears contradictory, as IA is actually more ancient than MA. The paragraph should exemplify the aspects in which they were similar, providing specific examples of individuals that exhibited similarity. Additionally, the author mentioned that the similarity was substantial but failed to provide any quantitative analysis.
We have corrected the manuscript accordingly.

##############################################################
Results, page 5, line 9: The resolutions of Figures S16-19 are too low and should be improved.
We have moved the supplementary figures from the supplementary materials to additional files and submitted them in PDF format with appropriate resolution.

##############################################################
Results, page 5, line 48: It is difficult to extract specific phenotypic predictions from Table S2. It would be helpful if the author could include additional columns displaying the probabilities of specific phenotypes, such as blond hair.
We have corrected the Table S2 accordingly.

##############################################################
Results, page 5, line 53: Figure S19 does not correspond to the description provided by the authors. Should it be Figure S20A instead?
Yes, we have corrected the manuscript accordingly.

##############################################################
Results, page 8, line 7-18: It is unclear why the author modeled the ancestry of an earlier group (IA_X) using a later group (MA_X). Further clarification is needed.
We were asked to perform this analysis by Reviewer 1. Its purpose was to test if the MA_X would represent the local population who mixed with the incoming populations to form the Wielbark Culture associated people. Revewer 1 suggested that positive result of this test indirectly support the hypothesis on genetic continuation from the IA to MA.

##############################################################
Discussion, page 9, line 47: There is no evidence indicating that the northern immigrants mixed with the autochthonous IA populations. Although the paper listed five populations that could approximate the local IA group, the use of qpAdm analysis to model the genetic ancestry of IA_X using northern IA populations and these five "local" IA populations may not be supported, as there might not have been a genetically differentiated local IA group in this region prior to the IA individuals sampled in this study.
Regarding the response to reviewer comment 2) on nomenclature, the author's respond: "We note however that when we use name Hungary_Langobard it is difficult to change this name as it is directly provided by the AADR resource and is used throughout the aDNA publications.". This is insufficient as an explanation The original Amorim manuscript never refer to the individuals as Lang/Longobards, but instead emphasizes: "whether the association between genetic ancestry and material culture reflects specific peoples mentioned in historical texts (i.e., Longobards) or stemmed from a deeper/long-term descent (of mixed barbarian ancestries) is as yet unclear." We would refer the authors to Eisenmann et al. Scientific Reports volume 8, Article number: 13003 (2018) for more discussion on this matter.
We agree with the Reviewer that the genetic data presented in the manuscript alone are not enough to verify all aspects of such complex hypothesis. However, our genetic data in combination with earlier observations and archaeological findings provides several lines of evidence supporting our statements. For example, in our previous paper we reported that in the early stage of the Wielbark culture formation (in Kowalewko population I-II century CE) the matrilineal genetic ancestries of males and females were different. The same phenomenon was not observed when autosomal DNA was analysed. This result indicates that immigrants were mainly males who mixed with the local females. This difference between females and males was not observed in the final stage of the Wielbark culture development. Moreover, we would like to emphasize that we have evaluated an extensive set of qpAdm models and none of them has contradicted the hypothesis assuming the migration of north population and its admixture with the local autochthons. Considering the above we modified the manuscript according Reviewer 2 suggestion.
Regarding the unfortunate “Hungary_Langobard” name, we have corrected the manuscript accordingly. We acknowledge that previously used shortcut could be misleading, even though many publications follow it. However, the latter does not mandate to spread this name induce the obviously improper connotation. Accordingly, we changed the shortname for this population of the form: Hungary_Szolad_Migration_Period
